# Snitches Get Stitches and End Up in Ditches: A Systematic Review of the Factors Associated With Whistleblowing Intentions

**DOI:** 10.3389/fpsyg.2021.631538

**Published:** 2021-10-05

**Authors:** Adam R. Nicholls, Lucas R. W. Fairs, John Toner, Luke Jones, Constantine Mantis, Vassilis Barkoukis, John L. Perry, Andrei V. Micle, Nikolaos C. Theodorou, Sabina Shakhverdieva, Marius Stoicescu, Milica V. Vesic, Nenad Dikic, Marija Andjelkovic, Elena García Grimau, Javier A. Amigo, Anne Schomöller

**Affiliations:** ^1^Department of Sport, Health, and Exercise Science, University of Hull, Hull, United Kingdom; ^2^Department of Physical Education and Sport Science, Aristotle University of Thessaloniki, Thessaloniki, Greece; ^3^Department of Psychology, Mary Immaculate College, Limerick, Ireland; ^4^Romanian National Anti-Doping Agency, Bucharest, Romania; ^5^KEA Fair Play Code Hallas, Athens, Greece; ^6^Faculty of Physical Education and Sport, National University of Physical Education and Sports, Bucharest, Romania; ^7^Anti-Doping Agency of Serbia, Belgrade, Serbia; ^8^Agencia Española de Protección de la Saluden el Deporte, Madrid, Spain; ^9^International Council of Sport Science and Physical Education, Berlin, Germany

**Keywords:** intentions, organizational, reporting, whistleblower, wrongdoing

## Abstract

Blowing the whistle on corruption or wrongdoing can facilitate the detection, investigation, and then prosecution of a violation that may have otherwise gone undetected. The purpose of this systematic review was to identify the factors that are associated with intentions to blow the whistle on wrongdoing. We searched Academic Search Premier, CINAHL Complete, Education Research Complete, ERIC, Medline, PsycARTICLES, PsycINFO, Regional Business News, and SPORTDiscus in January 2020. The quality of evidence was assessed using the Cochrane risk of bias tool. Of the 9,136 records identified, 217 studies were included in this systematic review. We identified 8 dimensions, 26 higher-order themes, and 119 lower-order themes. The whistleblowing dimensions were personal factors, organizational factors, cost and benefits, outcome expectancies, the offense, reporting, the wrongdoer, and social factors. Based on the findings, it is apparent that organizations should empower, educate, protect, support, and reward those who blow the whistle, in order to increase the likelihood on individuals blowing the whistle on corruption and wrongdoing. A combined approach may increase whistleblowing intentions, although research is required to test this assertion. From a policy perspective, more consistent protection is required across different countries.

## Introduction

Whistleblowing has brought several scandals to light in healthcare (Blenkinsopp et al., 2019), finance (Mehrotra et al., 2020), and sport (Whitaker et al., 2014). A widely used and accepted definition of whistleblowing is the “disclosure by organization members…of illegal, immoral, or illegitimate practices under the control of their employers, to persons or organizations that may be able to effect action” (Near and Miceli, 1985, p. 4). Whistleblowing represents an important mechanism of tackling corruption and wrongdoing. It is also socially significant because of its impact on employees, patients, students, organizations, and society in general (Culiberg and Mihelič, 2016). It is therefore unsurprising that whistleblowing has received considerable attention among scholars (Vandekerckhove and Lewis, 2012; Culiberg and Mihelič, 2016; Mannion et al., 2018), given that not everyone reports wrongdoing.

There is also a financial implication of corruption. It is estimated corruption exceeds €120 billion per year in the European Union (European Commission, 2018). Understanding more about the factors that are linked to a person's intention to blow the whistle is important, to help organizations promote this type of behavior, especially given the repercussions that some whistleblowers may encounter. The literature on whistleblowing, however, is very disparate. Studies have been conducted in many different areas (e.g., medicine, healthcare, finance, government, and sport). Further, some studies have been hypothetical, experimental, or recollections of actual whistleblowing behavior.

Scholars have argued that more information is required in explaining the process between a person observing wrongdoing and when deciding to report the wrongdoing, or remaining silent (Mesmer-Magnus and Viswesvaran, 2005; Culiberg and Mihelič, 2016). A psychological construct that predicts future behavior and is a key index of a person's readiness to take action is intentions (Sheeran, 2002). Systematically understanding intentions to blow the whistle represents an important step in understanding this behavior. This is especially true for whistleblowing, considering that it is a rather rare behavior that it is not manifested regularly in people's everyday life. Very few people have experienced whistleblowing. In this sense, intentions reflect the most proximal precursor of whistleblowing behavior (Ajzen, 2011). Due to the aforementioned factors, a systematic review that analyses intentions within hypothetical, experimental, and recollections of actual whistleblowing behavior in one article is warranted. This is because it would provide a comprehensive understanding of the most proximal determinant of whistleblowing behavior across different domains and within different contexts.

Although researchers have examined the relationship between intentions and whistleblowing, the results are relatively inconsistent (Chen and Lai, 2014). That is, deciding to blow the whistle can be a challenging decision-making process, in which the individual may consider whether it is his or her responsibility (Keil and Park, 2010), the type of wrongdoing (Miceli and Near, 1992), and also the personal consequences of blowing the whistle (Lennane, 2012). The European Barometer on corruption revealed that 81% of respondents failed to blow the whistle because of the potential of retaliation (European Commission, 2018). Further, the myth that whistleblowers are disgruntled and underperforming workers trying to harm their company, has now been dispelled. Indeed, whistleblowers are generally high-performing and highly committed workers who want to protect their company or organization from being engulfed in a crisis (Zeng et al., 2020).

Despite whistleblowing being crucial in tackling corruption and wrongdoing (European Commission, 2018), factors that may determine whether an individual will speak and report violations or remain quiet are equivocal (Chen and Lai, 2014). This might be because whistleblowing research has taken place in very diverse subject areas. Further, reviews on whistleblowing have tended to focus on one discrete area such as healthcare (e.g., Blenkinsopp et al., 2019) or financial corruption (e.g., Mehrotra et al., 2020). As such, scholars have failed to review the literature across all domains in which whistleblowing research has taken place. This would provide a more comprehensive account of the literature. To address this issue, the current study aimed to systematically review the literature regarding the factors that are associated with a person's intention to blow the whistle.

## Methods

### Information Sources and Search Strategy

In line with revised PRISMA guidelines (Page et al., 2021) and previous reviews (Nicholls et al., 2016, 2017), three distinct search strategies were utilized to obtain appropriate studies. The first strategy involved using search engines. For this review, the search engines examined were Academic Search Premier, CINAHL Complete, Education Research Complete, ERIC, Medline, PsycARTICLES, PsycINFO, Regional Business News, and SPORTDiscus were accessed in January 2020. No date limit was placed on the searches. The keywords of “whistle blowing” OR “whistleblower” OR “wrongdoing” were used in conjunction with “accounting,” AND “education,” AND “financial services,” AND “government agencies,” AND “healthcare,” AND “legal services,” AND “medicine,” AND “nursing,” AND “organizations,” AND “organizational,” AND “personal factors,” AND “personality,” AND “sport” was entered into these search engines. Pearl growing or referencing tracking was the second search strategy employed in this systematic review. This involved reviewing the references list of included studies for other appropriate articles (Hartley, 1990; Greenhalgh and Peacock, 2005). This iterative process was conducted until no new studies of relevance were identified.

The third search strategy used in this study involved searching the databases of peer-reviewed journals manually. Before commencing this strategy, it was decided that the focus of the search should be on those journals that had a history of publishing articles on whistleblowing. Using the results from the database and pearl growth search strategies, the journals that published three or more studies were deemed appropriate in this systematic review and included into this manual journal search strategy. These journals were *Journal of Business Ethics* (1982–2020), *Auditing: A Journal of Practice and Theory* (1999–2020), *International Journal of Law and Management* (2008–2020), *Behavioral Research in Accounting* (2001–2020), *Employee Responsibilities and Rights Journal* (1988–2020), *Ethics and Behavior* (1991–2020), *Journal of Nursing Care Quality* (1986–2020), *Human Relations* (1947–2020), *Accounting and the Public Interest* (2001–2020), *Journal of Advanced Nursing* (1976–2020), *Managerial Auditing Journal* (1986–2020), and *Public Personnel Management* (1972–2020). Through the database provided on each journal's website, the terms “whistleblowing” and “whistleblower” were searched with no date limits. The results of this process were reviewed in the same manner as the previous two strategies.

### Eligibility Criteria

**Figure 1:**
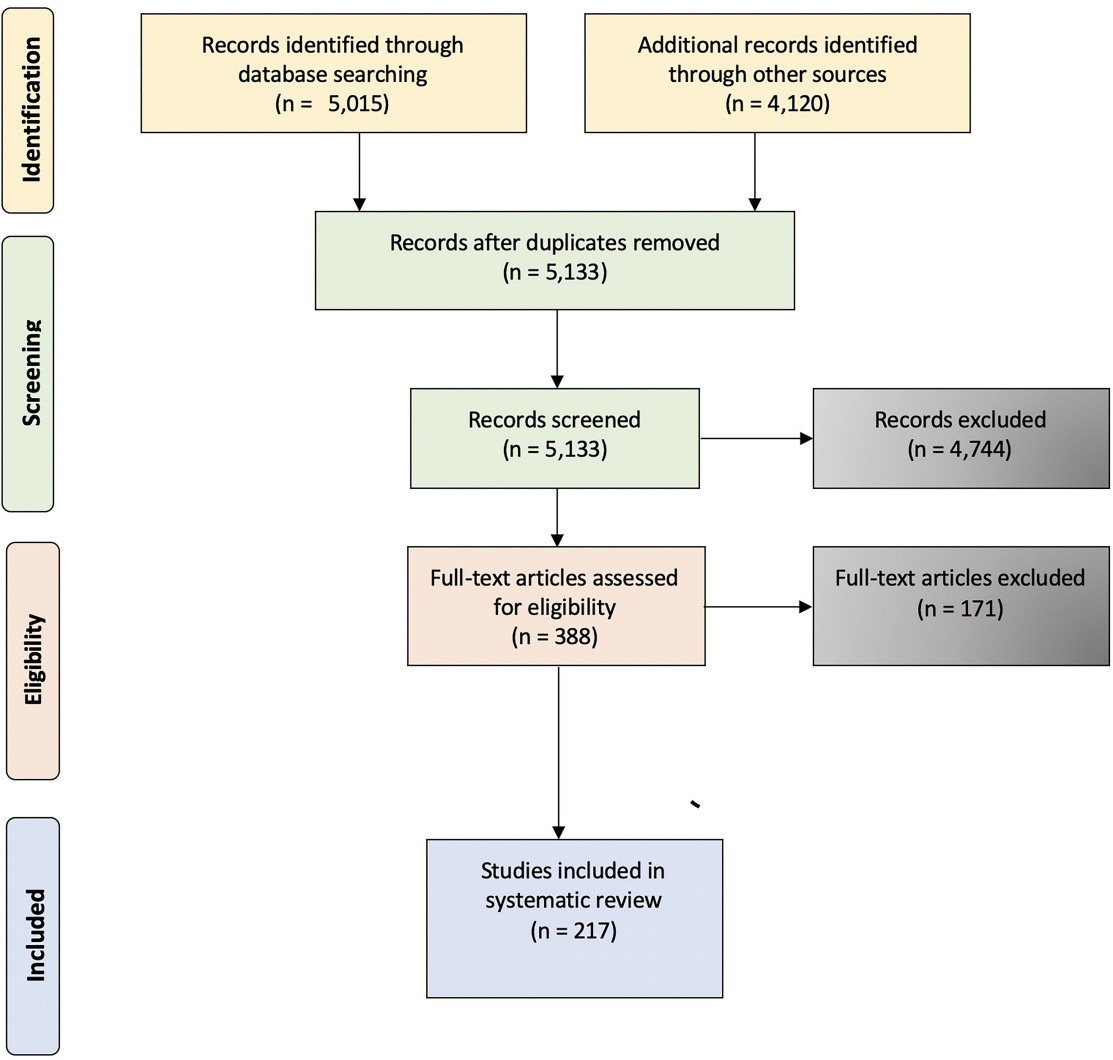
Prisma flow diagram.

The inclusion criteria used for this systematic review was that a study had to be primary research, published in peer-reviewed journals, and in English. Further, for inclusion in this systematic review, studies needed to assess factors that assessed intentions to report wrongdoing in either hypothetical or real-life situations. As such, whistleblowing behavior was not part of the inclusion criteria, but the factors linked to a person's conscious decision to either blow the whistle on wrongdoing or remain quiet. Research published in non-peer reviewed articles, systematic-reviews, book chapters, meta-analysis, and peer-review articles not in English where therefore excluded based on not meeting the inclusion criteria. As indicated in Figure 1, the total number of identified records was 9,135. Following the removal of 4,003 duplicates, the titles and abstracts of 5,133 records were screened for those entries that were deemed irrelevant (e.g., editorials; Lefebvre et al., 2019). Through this initial screening process, 4,744 studies were excluded from the systematic review. For the remaining 388 records, a second assessment was conducted on the full-text reports (Lefebvre et al., 2019). After reviewing these papers, 171 studies were excluded from the review because either they were deemed irrelevant, there was no access to the full paper, or the study did not meet the eligibility criteria. From this process, a total of 217 studies were included in this systematic review.

### Assessment of Methodological Quality and Risk of Bias

The Cochrane Collaboration's Risk of Bias tool (Higgins et al., 2011), which was adapted by Ntoumanis et al. (2014), was used to assess the risk of bias in the studies deemed eligible for the systematic review. This tool serves as a framework for assessing bias within cross-sectional, longitudinal, and experimental studies, and generates an overall score of low, unclear, or high risk of bias. A study that scored low risk across all criteria were considered low risk. A study that scored high risk on one criterion was considered high risk, and a study that scored unclear on one criterion was scored as unclear. Please see Supplementary Table 1 for criteria scores for each study and Supplementary Table 2 for overall risk bias evaluations.

In order to undertake the methodological quality and risk of bias assessment, the second author (Lucas Fairs) independently assessed all papers on the criteria recommended by (Ntoumanis et al., 2014). The lead author (Adam Nicholls) independently applied the same criteria to assess methodological quality and risk of bias within 50 papers chosen at random. There was one paper that required discussion, but after this was resolved, there was a 100% agreement between Fairs and Nicholls.

### Inductive Content Analysis

In order to group the findings of papers into dimensions, higher-order, and lower-order themes, and in accordance with the systematic review by Nicholls et al. (2017), all of the studies were subjected to an inductive and deductive content analyses procedure, as outlined by Maykut and Morehouse (2002). Similar factors that predicted whistleblowing intentions were grouped together as dimensions. Within each dimension, there were higher-order and lower-order themes. Each dimension, and higher-, and lower-order theme was given a descriptive label and a rule of inclusion was constructed for each dimension. The rule of inclusion for the dimension personal factors was “person-based constructs that are related to intentions to blow the whistle.” The rule of inclusion for morality was “principles about what is right and what is wrong,” whereas the lower-order theme moral intensity was “the strength of a person's morals.”

## Results

### Study Characteristics

From the 217 articles that investigated factors associated with whistleblowing intentions, a total of 289,458 individuals participated in the studies (*M* =1340.08, *SD* = 5627.54, *Mdn* = 224.5). The number of involved participants ranged between five (Nurhidayat and Kusumasari, 2019) and 42,020 (Caillier, 2017a). These individuals were recruited from a variety of populations and countries. In terms of populations, samples used in the analyzed studies included university students (e.g., Keil et al., 2007; Kennett et al., 2011), government employees (e.g., Miceli and Near, 1985; Lavena, 2016), accountants and auditors (e.g., Liyanarachchi and Adler, 2011; Zheng et al., 2019), healthcare professionals (nurses, obstetricians, midwives; e.g., Vincent et al., 1999; Ahern and McDonald, 2002), athletes (e.g., Whitaker et al., 2014; Erickson et al., 2017), managers and professional staff (e.g., Schultz et al., 1993), researchers (e.g., Satalkar and Shaw, 2018), police officers (e.g., Rothwell and Baldwin, 2007a,b), and teachers (e.g., Richardson et al., 2008). As for countries, participants were recruited from the U.S., Canada, Australia, Malaysia, Iran, Barbados, the U.K. (including Scotland), Turkey, India, South Africa, Norway, the Netherlands, Ireland, Taiwan, China (including Hong Kong), Israel, Malta, the Philippines, Singapore, South Korea, Indonesia, New Zealand, Mexico, Uganda, Botswana, Mauritius, Italy, Switzerland, France, Croatia, Austria, Germany, and Liechtenstein. Finally, most studies recruited male and female participants. One exception to this trend is Lee et al. (2004), who investigated females only. See Supplementary Table 2 for a description of the study characteristics.

The majority of reviewed studies used a quantitative design (*n* = 194; 89.40%). The remaining investigations implemented either a qualitative (*n* = 13; 5.99%) or a mixed-methods (*n* = 10; 4.61%) approach. Most of the studies involved participants being presented with scenarios of wrongdoing and assessed their intentions to report wrongdoing in a specific hypothetical scenario (*n* = 117; 53.9%), their general views on whistleblowing and factors that would determine whether wrongdoing is reported (*n* = 70; 32.3%), or factors that associated with intentions during past events (*n* = 30; 13.8%). A summary of the study designs is presented in Supplementary Table 2.

### Factors That Predict Whistleblowing Intentions

Eight dimensions were identified as factors that were associated with an individual's intention to blow the whistle on wrongdoing. These were personal factors, organizational factors, cost and benefits, outcome expectancies, the offense, reporting, the wrongdoer, and social factors. Across these eight dimensions, there was a total of 26 higher-order themes and 119 lower-order themes (see Table 1).

**Table 1:** Dimensions, higher order, and lower order themes.

**Dimension**	**Higher-order theme**	**Lower-order theme**
Personal	Demographics	Age
factors		Gender
		Culture
		Education
		Religion
		Experience
		Work history
		Managerial experience
		Duration with organization
	Morality	Moral intensity
		Moral identity
		Moral reasoning
		Moral competence
		Moral perception
		Moral conviction
		Moral development
		Moral obligation
		Moral courage
		Moral disengagement
	Attitudes	Whistleblowing
		Policy
		Wrongdoing
		Money
	Personality	Honesty
		Risk aversion
		Locus of control
		Traits
		Personal disposition
		Mood
		Individual propensity
	Beliefs	Self-confidence
		Self-efficacy
		Perceived behavioral control
	Emotion	Anger
		Anxiety
		Hopelessness
	Ethical	Personal ethics
		Legitimacy
		Adherence to principals
		Ethical training
		Professional ethics
	Feelings toward	Trust
	organization	CommitmentIntentions to stay/leave
		Role identity
		Value of whistleblowing within organization
	Responsibility	Personal responsibility
		Role responsibility
		Displacement of responsibility
		Need to correct wrongdoing
		Justice
	Job	Job security
		Job satisfaction
		Workload
		Role benefits
		Salary
		Performance evaluation
Organizational	Characteristics	Structure
factors		Size
		Unionization
		Type of industry
		Stability
		Reputation
		Professional standards
		Identification
		Dependence on wrongdoing
		Attachment to project
	Leadership	Ethical management
		Managerial reactions
		Communication
		Manager practices
		Dissimilarity between manager and employee
	Moral Code	Ethics
		Climate
		Regulations
	Protection	Legal protection
		Previous Incidents
		Policies/procedures
Costs and	Costs	Perceived personal costs
Benefits	Benefits	Financial
		Incentives
		Personal
		Societal
		Benefit-to-cost differential
Outcome	Organizational	Impact
expectancies		Effectiveness
		Expectancy
	Personal	Future career
		Hostility
The offense	Characteristics	Severity
		Type
		Frequency
		Intentionality
		Duration
Reporting	Mechanisms	Anonymized vs. Non-anonymized
		Reporting system
		Channel of communication
		Processes
		Opportunities
The	Demographics	Status/Rank
Wrongdoer		Fault
		Reputation
	Relationship	Status/Rank of whistleblower in relation to wrongdoer
		Wrongdoer's knowledge of potential whistleblower
		Relationship with wrongdoer
	Purposes	Punish/Hurt wrongdoer
		Help
Social factors	Group	Presence of bystanders
		Cohesion
		Interests
		Social confrontation
		Peer invalidation of wrongdoing
		Approval
	Support	Social support
		Supportive communication among peers
	Norms	Cultural norms
		Social norms
		Subjective

### Personal Factors

Eight higher-order themes were identified within the personal factors dimension. These were: demographics, morality, attitudes, state and trait constructs, ethics, feelings toward the organization, responsibility, and work-life.

#### Demographics

Demographic lower-order themes included age, gender, education, religion, experience, position within a company, and nationality. Contrasting evidence was found across the two studies that examined the relationship between age and intentions to blow the whistle. Brennan and Kelly (2007) reported that the willingness to report wrongdoing externally decreased with age, whereas Sims and Keenan (1998) reported that whistleblowing intentions were not predicted by age.

Twelve studies examined the effect of gender of whistleblowing, with five studies reporting that females were more likely to blow the whistle than males (Erkmen et al., 2014; Brown et al., 2016; Fieger and Rice, 2018), especially when there are anonymous reporting mechanisms (Kaplan et al., 2009b) or no laws have been broken (Su et al., 2010). Seven studies reported that males had stronger whistleblowing intentions than females (e.g., Miceli and Near, 1988; Miceli et al., 1991b; Sims and Keenan, 1998; Liyanarachchi and Adler, 2011; Gökçe, 2013c; Taylor and Curtis, 2013; Yu et al., 2019).

Six studies examined general education and whistleblowing education in relation to intentions to blow the whistle. Educational levels were positively associated with whistleblowing intentions (e.g., Sims and Keenan, 1998; Cho and Song, 2015), as was education in whistleblowing (e.g., McManus et al., 2012; Caillier, 2017a; Yu et al., 2019). Two studies explored how religion was associated with whistleblowing intentions, with both studies finding that the strength of one's faith was positively linked to intentions to blow the whistle (Bocchiaro et al., 2012; Gökçe, 2015). In regard to experience, two studies revealed that experience was positively linked to whistleblowing intentions (Gökçe, 2013c), whereas a lack of experience or tenure was linked to people being less likely to blow the whistle (Milliken et al., 2003). Finally, Brown et al. (2016) reported that senior level individuals had stronger whistleblowing intentions than middle or lower-level employees. In contrast to this, four studies found that lower-level employees, such as supervisors, had stronger whistleblowing intentions (Miceli and Near, 1984; Rothwell and Baldwin, 2006, 2007a,b) than senior managers.

In terms of nationality, four studies found that U.S citizens were more likely to blow the whistle than Jamaicans (Sims and Keenan, 1999), Singaporeans (Tan et al., 2003), Chinese (Keenan, 2007), and Croatians (Tavakoli et al., 2003). However, Keenan (2002) found no differences in whistleblowing intentions between U.S. and Indian citizens. Whistleblowing intentions increased for U.S. individuals if they could shift the blame, but this did not increase intentions for Korean participants (Keil et al., 2007). MacNab et al. (2007) reported that U.S. and Canadian citizens were less likely to blow the whistle on more powerful people, but this was not associated with the intentions of Mexican people.

#### Morality

The higher-order theme morality contained 9 lower-order themes, which included moral intensity, moral identity, moral reasoning, moral competence, moral perception, moral conviction, moral development, and moral courage. Of the seven studies that examined the relationship between moral intensity and whistleblowing, six studies reported a positive relationship (e.g., Taylor and Curtis, 2010, 2013; Bhal and Dadhich, 2011; Proost et al., 2013; Chen and Lai, 2014; Latan et al., 2019a), but one study only found a partial and positive relationship between these two constructs (Latan et al., 2018).

Moral reasoning (Xu and Ziegenfuss, 2008; Liyanarachchi and Newdick, 2009), moral competence (MacGregor and Stuebs, 2014), moral perception (Keenan, 2000), moral conviction (Stikeleather, 2016), moral development (Brabeck, 1984; Miceli et al., 1991a,b), and moral courage (Cheng et al., 2019) were all positively associated with whistleblowing intentions. Finally, moral disengagement was associated with individuals not intending to blow the whistle on wrongdoing (Ion et al., 2016).

#### Attitudes

The attitude higher-order theme was concerned with how attitudes toward whistleblowing, policy, wrongdoing, and money were linked to whistleblowing intentions. Twelve studies reported that favorable attitudes toward whistleblowing positively predicted whistleblowing intentions (e.g., Ellis and Arieli, 1999; Lim and See, 2001; Park and Blenkinsopp, 2009; Richardson et al., 2012; Trongmateerut and Sweeney, 2013; Kamarunzaman et al., 2014; Brown et al., 2016; Alleyne et al., 2018, 2019; Latan et al., 2018; Nurhidayat and Kusumasari, 2019). Further, teachers' who had more favorable attitudes toward a school's policy on examinations were more likely to blow the whistle than teachers with less favorable attitudes (Richardson et al., 2008). Favorable attitudes toward money were associated with stronger intentions to blow the whistle among business students, than those with less favorable attitudes toward money, when financial incentives were provided to report wrongdoing (Brink et al., 2017). Finally, Cassematis and Wortley (2013) found that public sector employees from Australia act in a manner that matches their attitude. That is, individuals who have a more favorable attitude to whistleblowing are more likely to blow the whistle than people with a less favorable attitude. It should be noted however, that situational factors such as the extent to which a person suffered as a consequence of wrongdoing and the perceived seriousness or wrongdoing were stronger predictors of whistleblowing intentions than attitudes.

#### Personality

Eight personality factors predicted intentions to blow the whistle on wrongdoing. These included honesty, risk aversion, locus-of-control, traits, personal disposition, and individual propensity. Honesty was positively associated with whistleblowing intentions within two studies (Keil et al., 2007; Radulovic and Uys, 2019), whereas risk aversion was negatively associated with whistleblowing intentions across three studies (e.g., Pillay et al., 2012, 2017; Zhou et al., 2018). Three studies (Chiu, 2002, 2003; Chiu and Erdener, 2003) found that locus-of-control was negatively associated with intentions to blow the whistle. That is, individuals with low levels of perceived control (i.e., external loci) were less likely to blow the whistle on wrongdoing. A proactive personality type was positively associated with whistleblowing intentions within two studies (Miceli et al., 2012; Liu et al., 2016), although one study did not find a link between personality type and whistleblowing intentions (McCutcheon, 2000). Conversely, Machiavellianism (i.e., an individual who manipulates others, is deceitful, and thinks only about him or herself; Hern et al., 2006) was negatively associated with whistleblowing intentions (Dalton and Radtke, 2013). One study found that those with the strongest intentions to blow the whistle had either a personal disposition (Ion et al., 2016) or a propensity to do so (Keenan, 2000; MacNab and Worthley, 2007).

#### Beliefs

Self-Confidence, self-efficacy, and perceived behavioral control were the lower-order themes for beliefs. Self-confidence (Nurhidayat and Kusumasari, 2019) and self-efficacy (MacNab and Worthley, 2007) were positively associated with intentions to blow the whistle. All six studies that examined the relationship between whistleblowing intentions and perceived behavioral control found a positive association (Park and Blenkinsopp, 2009; Brown et al., 2016; Rustiarini and Sunarsih, 2017; Surya et al., 2017; Alleyne et al., 2018, 2019). The control to benefit others (i.e., pro-social control) was also positively associated with whistleblowing intentions in one study (Stansbury and Victor, 2009).

#### Emotions

Six studies explored how emotions were related to whistleblowing intentions. Of these six studies, two studies (Whitaker et al., 2014; Latan et al., 2019b) found a relationship between emotions and whistleblowing intentions. It should be noted that neither Latan et al. (2019b) nor Whitaker et al. (2014) specified which emotion or emotions were linked to whistleblowing. More specifically, two studies found a positive relationship between anger and whistleblowing intentions (Gundlach et al., 2008; Jones et al., 2014) and one study found that anxiety about wrongdoing was linked to a higher likelihood of endorsing whistleblowing (Henningsen et al., 2013). Finally, when individuals felt hopelessness, they were less likely to blow the whistle on wrongdoing (Ion et al., 2016).

#### Ethical

The five lower-order themes personal ethics, legitimacy, adherence to principles, ethical training, and professional ethics were categorized in the ethical higher-order theme. In regard to personal ethics, there were very mixed results. Three studies found a positive relationship (King and Hermodson, 2000; Park et al., 2005; Zarefar and Zarefar, 2017), two studies found no relationship (Clements and Shawver, 2009; Gökçe, 2013d), and two studies found a negative relationship between personal ethics and whistleblowing intentions (Gökçe, 2013d; Pillay et al., 2018). Perceived legitimacy (Mbago et al., 2018) and adherence to one's principles (Radulovic and Uys, 2019) were both positively related to whistleblowing intentions. Ethics training was reported across three studies (Shawver, 2011a; McManus et al., 2012; Yu et al., 2019). Ethics training increased whistleblowing intentions. Finally, professional ethics was also positively associated with whistleblowing in two studies (Pillay et al., 2012; King and Scudder, 2013).

#### Feelings Toward Organization

Trust, commitment, intentions to stay or leave, role identity, and value of whistleblowing within organization, were identified as lower-order themes within the feeling toward organization theme. Trust in one's organization and management were positively associated with intentions to blow the whistle across 16 studies (Attree, 2007; Brennan and Kelly, 2007; Binikos, 2008; Curtis and Taylor, 2009; Keil et al., 2010; Seifert et al., 2014; Alleyne, 2016; Lavena, 2016; Arifah et al., 2017; Guthrie and Taylor, 2017; Aydan and Kaya, 2018; Fleming et al., 2018; Taylor, 2018; Taylor and Curtis, 2018; Wilson et al., 2018; Ugaddan and Park, 2019). Commitment to one's organization was positively associated with whistleblowing intentions across 10 studies (Sims and Keenan, 1998; Taylor and Curtis, 2010, 2018; Caillier, 2013; Chen and Lai, 2014; Alleyne, 2016; Surya et al., 2017; Alleyne et al., 2018, 2019; Latan et al., 2018). However, Somers and Casal (1994) found individuals with moderate levels of commitment were the most likely to blow the whistle. Intentions to stay or leave a company were not associated with whistleblowing intentions (Casal and Bogui, 2008). Possessing a strong role identity increased the chances of whistleblowing in one study (Grube et al., 2010). Individuals were more likely to blow the whistle when they perceived that their organization valued whistleblowing (Pillay et al., 2012; Cassematis and Wortley, 2013).

#### Responsibility

Personal responsibility, role responsibility, displacement of responsibility, need to correct wrongdoing, and wanting justice were identified as the lower-order themes for higher-order theme labeled responsibility. Of the 19 studies that examined the relationship between personal responsibility and whistleblowing intentions, 18 found a positive relationship between these constructs (Schultz et al., 1993; Kaplan and Whitecotton, 2001; Smith et al., 2001; Keil et al., 2004, 2010; Curtis, 2006; Gundlach et al., 2008; Park et al., 2008, 2009; Park and Keil, 2009; Dalton and Radtke, 2013; Gao et al., 2015; Lowe et al., 2015; Alleyne et al., 2017, 2018, 2019; Brink et al., 2017; Latan et al., 2018). Although Ayers and Kaplan (2005) also found a positive relationship between personal responsibility and whistleblowing intentions, this relationship was only observed when whistleblowing channels were anonymized. Role responsibility was examined in five studies, and all these studies reported that when whistleblowing was part of one's role in an organization, individuals had stronger whistleblowing intentions (Miceli and Near, 1984; Trevino and Victor, 1992; Victor et al., 1993; Keil et al., 2007; Casal and Bogui, 2008). Intentions to blow the whistle were linked to the need to correct wrongdoing in one study (Alleyne et al., 2013) and the desire to ensure justice, either social (Soni et al., 2015; Omotoye, 2017) or organizational (Victor et al., 1993; Seifert et al., 2010, 2014; Gökçe, 2013e; Pillay et al., 2017).

#### Job

This higher-order theme contained six lower-order themes: job security, job satisfaction, workload, role benefits, salary, and performance evaluation. When job security was high, individuals had stronger intentions to blow the whistle (Shawver, 2008), although when an individual feared losing their job, he or she was less likely to blow the whistle on wrongdoing (Alleyne, 2016). Four studies examined the relationship between job satisfaction and whistleblowing intentions, with contrasting findings. Three studies found a positive relationship between job satisfaction and whistleblowing intentions (Miceli and Near, 1988; Alleyne et al., 2013; Yu et al., 2019), whereas one study found no relationship between these constructs (Sims and Keenan, 1998).

Having a high workload was negatively associated with whistleblowing intentions (Vincent et al., 1999), as was being on a lower salary (Miceli et al., 1991b). However, receiving personal benefits (Alleyne et al., 2013) were positively linked to whistleblowing intentions. In regard to job performance evaluations, Miceli et al. (1991b) found that individuals who received a negative evaluation of their job performance were less inclined to blow the whistle, whereas Robertson et al. (2011) reported whistleblowing intentions were greatest for wrongdoers who were poor performers.

### Organizational Factors

Five higher-order themes were categorized within the organizational dimension, which were characteristics, leadership, support, moral code, and protection.

#### Characteristics

The lower-order themes for characteristics were structure, size, unionization, type of industry, reputation, professional standards, and attachment to a project. Four studies examined the relationship between the structure of an organization and intentions to blow the whistle on wrongdoing. A hierarchical structure was negatively associated with whistleblowing intentions in three studies (Milliken et al., 2003; Park and Keil, 2009; Satalkar and Shaw, 2018), although another study found that an organization's structure could have a positive impact on whistleblowing (Alinaghian et al., 2018). The size of an organization was negatively associated with whistleblowing (Barnett, 1992; Liu and Ren, 2017), as was unionization (Barnett, 1992). An organization's reputation was positively associated with whistleblowing intentions (Keil et al., 2007; Pillay et al., 2012) as were an organization's professional standards (Rennie and Crosby, 2002; Curtis and Taylor, 2009). Finally, if wrongdoing occurred when an organization was attached to or relied heavily on a particular project, individuals were more likely to blow the whistle (Keil et al., 2010).

#### Leadership

The higher-order leadership theme contained five lower-order themes, which included ethical management, managerial reactions, communication, leadership style, and dissimilarity between manager and employee. Eleven studies reported a positive relationship between ethical management and whistleblowing intentions (Chiasson et al., 1995; Near et al., 2004; Keil et al., 2010; Bhal and Dadhich, 2011; Kaptein, 2011; Alleyne, 2016; Wen and Chen, 2016; Zhang et al., 2016; Cheng et al., 2019; Ugaddan and Park, 2019; Hechanova and Manaois, 2020). If an individual thought that a manager may react negatively to whistleblowing or had reacted negatively in the past, people were less intent on blowing the whistle (Perry et al., 1997; Alleyne et al., 2013; Zhang et al., 2013; Scheetz and Fogarty, 2019). One study reported that aversive leaders elicited greater whistle-blowing intentions in financially unstable organizations (Thoroughgood et al., 2011). Communication between workers and management was examined across five studies and communication quality with management was positively associated with whistleblowing intentions in all of these studies (Richardson et al., 2008; Zhang, 2008; Lyndon et al., 2012; Kamarunzaman et al., 2014; Chaudhary et al., 2019). In terms of leadership style, transformational leadership (Caillier, 2013), laissez-faire style (Erturk and Donmez, 2016), and an authentic style (Liu et al., 2015) were positively associated with whistleblowing intentions. Finally, when there was a larger demographic dissimilarity (e.g., salary, education, sociogenic status) between a manager and an employer, individuals had weaker intentions to blow the whistle on wrongdoing (Park and Keil, 2009).

#### Moral Code

An organization's moral code included the lower-order themes of ethics, climate, values, and regulations. An organization's ethics was positively associated with whistleblowing intentions. That is, whistleblowing intentions were greater among organizations who were ethical in nine studies (Thoroughgood et al., 2011; Dalton and Radtke, 2013; Liu et al., 2016; Zhang et al., 2016; Aydan and Kaya, 2018; Taylor and Curtis, 2018; Tumuramye et al., 2018; Zhou et al., 2018; Scheetz and Fogarty, 2019). The climate in which employees worked in was also related to whistleblowing across seven studies. In five studies (Keil et al., 2004, 2010; Rothwell and Baldwin, 2007b; Ahmad et al., 2014; Alleyne, 2016) a highly ethical working climate was positively associated with whistleblowing intentions. However, ethical climate was stronger among US citizens than it was for Singaporeans (Tan et al., 2003), whereas when individuals were expected to remain silent, individuals were less likely to blow the whistle (Park and Keil, 2009). Finally, if an organization was highly regulated, individuals were much more likely to blow the whistle, in comparison to organizations that were not highly regulated (Miceli et al., 1991b).

#### Protection

The level of protection an organization provided an individual was categorized into the lower order themes of legal, previous incidents, and policies and procedures. If an organization had a strong legal system and would externally prosecute individuals who committed immoral or illegitimate acts (Pillay et al., 2012, 2017) or lacked protective legislation (Zipparo, 1999), individuals were less intent on blowing the whistle. The way in which an organization managed previous whistleblowing intentions was investigated across four studies. Three of these studies found that if an organization had previously dealt with a previous report of wrongdoing well, individuals were more likely to blow the whistle (Perry et al., 1997; King and Hermodson, 2000; Scheetz and Fogarty, 2019). However, if an organization had managed previous whistleblowing incidents negatively, introducing an externally administered hotline increased whistleblowing intention (Zhang et al., 2013). The extent to which organizations had policies and procedures to protect whistleblowers was also linked to intentions. That is, organizations with policies and procedures to protect individuals was linked to greater whistleblowing intentions (Xu and Ziegenfuss, 2008; Cho and Song, 2015; Wainberg and Perreault, 2016; Omotoye, 2017; Olesen et al., 2019).

### Cost and Benefits

This dimension contained two higher-order themes, which were personal costs and benefits. The benefits higher-order dimension comprised of financial, incentives, personal, societal, and benefit-to-cost differential lower-order themes.

#### Personal Costs

Fifteen studies (Schultz et al., 1993; Kaplan and Whitecotton, 2001; Ayers and Kaplan, 2005; Curtis, 2006; Kaplan et al., 2009a,b; Kennett et al., 2011; Cho and Song, 2015; Gao et al., 2015; Alleyne et al., 2017, 2018, 2019; Latan et al., 2018; Chaudhary et al., 2019) reported that personal costs such as monetary or job loss were negatively associated with whistleblowing intentions. Interestingly, Oelrich (2019) found that whistleblowing intentions were more strongly decreased by monetary losses than increased by monetary gains.

#### Benefits

Monetary benefits to blow the whistle on wrongdoing was positively associated with whistleblowing intentions in 10 studies (Miceli and Near, 1984; Xu and Ziegenfuss, 2008; Pope and Lee, 2013; Stikeleather, 2016; Berger et al., 2017; Guthrie and Taylor, 2017; Andon et al., 2018; Rose et al., 2018; Latan et al., 2019c; Teichmann, 2019). Further, Rose et al. (2018) and Stikeleather (2016) both found that the size of the financial reward was positively linked to whistleblowing intentions. Three studies assessed the impact of incentives to blow the whistle. Two of the studies found that incentives increased whistleblowing intentions (Brink et al., 2013; Berger et al., 2017), but Boo et al. (2016) found that incentives to blow the whistle do not increase whistleblowing intentions when an individual has a close relationship with the wrongdoer. Personal benefits were positively associated with whistleblowing intentions (Alleyne et al., 2013, 2017), especially for individuals who score highly on the Machiavellianism personality trait (Dalton and Radtke, 2013), as were societal benefits (Kennett et al., 2011). Finally, whistleblowing intentions were higher when individuals perceived that the benefits outweighed the costs of this behavior (Keil et al., 2010).

### Outcome Expectancies

This dimension contained expectancies regarding the organization and personal, as consequence of whistleblowing. The organizational higher-order dimension contained three lower-order themes: impact, effectiveness, and expectancy. The lower-order themes for personal were future career and hostility.

#### Organizational

Two studies found that individuals were less likely to blow the whistle on wrongdoing if they felt that this may be detrimental to the organization (Alleyne et al., 2013; Hwang et al., 2014). The perceived effectiveness of an organization's ability to handle whistleblowing was positively associated with whistleblowing intentions (Casal and Bogui, 2008). Finally, when individuals expected that an organization would act upon whistleblowing, intentions were higher than in organizations where there was a low expectancy (Tumuramye et al., 2018).

#### Personal

Two studies explored how individual's evaluation of their future career prospects, as a consequence of whistleblowing, impacted intentions. Both studies found that individuals who expected that their career development would suffer, were less intent on blowing the whistle (Liu and Ren, 2017; Fleming et al., 2018). If individuals anticipated hostility in the form of intimidation (Lyndon et al., 2012), they were less intent on blowing the whistle.

### The Offense

There was one higher-order theme for the offense, or the act of wrongdoing, which was termed characteristics. Characteristics comprised of five lower-order themes, which were severity, type, frequency, intentionality, and duration.

#### Characteristics

The relationship between severity of the wrongdoing and whistleblowing intentions was examined in 14 studies (Schultz et al., 1993; King, 1997; Ayers and Kaplan, 2005; Curtis, 2006; Richardson et al., 2012; Ahmad et al., 2013, 2014; Alleyne et al., 2013; MacGregor and Stuebs, 2014; Brink et al., 2017; Caillier, 2017b; Andon et al., 2018; Nawawi and Salin, 2018; Latan et al., 2019b). All 14 of these studies found a positive relationship between the severity of the wrongdoing and whistleblowing intentions. The type of wrongdoing was positively associated with whistleblowing intentions in four studies (Lee et al., 2004; Somers and Casal, 2011; Brink et al., 2017; Taylor, 2019), although Scheetz and Wilson (2019) found that all types of wrongdoing were positively associated with whistleblowing among not-for-profit employees. Six studies (Brooks and Perot, 1991; Lee et al., 2004; Rothwell and Baldwin, 2006; Grube et al., 2010; Lyndon et al., 2012) examined the extent to which the frequency of wrongdoing was linked to whistleblowing intentions. All six studies found that the frequency of wrongdoing was positively linked to intentions to blow the whistle. Individuals that witnessed intentional wrongdoing were more likely to blow the whistle in comparison to accidental wrongdoing (Keil et al., 2018). Finally, Lee et al. (2004) found that the duration of wrongdoing was positively associated with whistleblowing intentions.

### Reporting

The reporting dimension contained one higher-order theme, named mechanisms. Within mechanisms, there were five lower-order themes: anonymized vs. non-anonymized, reporting system, channel of communication, processes, and opportunities.

#### Mechanisms

Eleven studies examined the extent to which anonymized vs. non-anonymized whistleblowing channels were linked to intentions to blow the whistle. There were some contrasting findings as five studies indicated anonymized reporting channels were linked positively to whistleblowing intentions (Kaplan et al., 2009b; Keil et al., 2010; Atkinson et al., 2012; Alleyne et al., 2013, 2017). Five studies, however, reported that anonymized whistleblowing channels were not associated with increased whistleblowing intentions, in comparison to protected identity channels (Miceli and Near, 1984; Curtis and Taylor, 2009; Gökçe, 2013a,b; Pope and Lee, 2013). Ayers and Kaplan (2005) found that it was the perceived seriousness of the wrongdoing rather than whether the reporting channel was anonymized that was more strongly linked to whistleblowing intentions. The channel of communication and its association with whistleblowing intentions was examined within five studies. Individuals were more likely to blow the whistle via internal channels in comparison with externally run whistleblowing channels (Chen and Lai, 2014; Kamarunzaman et al., 2014; Chaudhary et al., 2019). On the contrary, Smith et al. (2001) found individuals were more reluctant to blow the whistle through internal channels, as did Gao et al. (2015) who found whistleblowing intentions were higher for third-party administered reporting channels. The process of whistleblowing across different organizations also impacted intentions. For example, a lack of confidence (Fleming et al., 2018), a complicated process (Francalanza and Buttigieg, 2016), a sub-certification process (Lowe et al., 2015), or a tedious investigative process were also associated with decreased intentions to blow the whistle on wrongdoing. Being aware and having the opportunity to blow the whistle was positively reported with intentions in the study by Latan et al. (2019c).

### The Wrongdoer

The wrongdoer dimension comprised of three higher-order themes, which were demographics, relationship, and purposes. The demographic higher-order theme contained the lower-order themes status/rank, fault, and reputation, whereas the relationship higher-order theme contained the lower-order themes status/rank of whistleblower in relation to wrongdoer, the wrongdoer's knowledge of potential whistleblower, and relationship with wrongdoer. Finally, the purposes higher-order theme contained punish/hurt the wrongdoer and help the wrongdoer.

#### Demographics

Five studies explored how the status of the wrongdoer was associated with the intentions of individuals to blow the whistle. Four studies found that individuals were less intent on blowing the whistle on wrongdoers who were superior to them in an organization (Miceli et al., 1991b; Ahmad et al., 2013; Gao et al., 2015; Chaudhary et al., 2019). Taylor and Curtis (2013) found that individuals have stronger intentions to blow the whistle on their peers, in comparison to a superior person. There were stronger intentions to blow the whistle on wrongdoers if they were perceived to be at fault (Park et al., 2008) and if they had a poorer reputation (Robertson et al., 2011).

#### Relationship

In regard to the relationship higher-order theme, one study found that whistleblowing intentions decreased if the whistleblower knew the individual (Robinson et al., 2012). Six studies examined the quality of the relationship with a wrongdoer and how it affected whistleblowing intentions. Each reported that relationship closeness with the wrongdoer was negatively associated with whistleblowing intentions (Milliken et al., 2003; Hwang et al., 2008; Alleyne et al., 2013; Erickson et al., 2017; Satalkar and Shaw, 2018; Olesen et al., 2019). That is, people had stronger intentions to blow the whistle on people they were not very close to.

#### Purpose

There were two lower-order themes for the purpose higher-order theme. One study reported that intentions to blow the whistle were higher when a person wanted to hurt the wrongdoer (Rennie and Crosby, 2002), whereas Moore and McAuliffe (2010) found that the fear of hurting a colleague was associated with lower whistleblowing intentions. The Rennie and Crosby (2002) study also found that whistleblowing intentions were associated with helping the wrongdoer.

### Social Factors

The social dimension included three higher-order themes, which were group, support, and norms. The group higher theme comprised of the presence of bystanders, cohesion, interests, social confrontation from work colleagues to whistleblower, peer invalidation of wrongdoing, and approval. Support included social support, supportive communication among peers, receptivity, and support during previous whistleblowing. The norms higher-order theme included three lower-order themes, which were cultural norms, social norms, and subjective norms.

#### Group

The presence of other bystanders, who had observed the wrongdoing, was associated with reduced intentions to blow the whistle (Gao et al., 2015), especially if there were few witnesses (Miceli et al., 1991a). Four studies examined the relationship between group cohesion and whistleblowing intentions. Three studies indicated that cohesion was positively associated with whistleblowing intentions (Rothwell and Baldwin, 2007b; Latan et al., 2018; Alleyne et al., 2019), whereas one study found that cohesion was negatively associated with whistleblowing intentions (Whitaker et al., 2014). Whistleblowing intentions increased if members of a group were negatively affected by wrongdoing (Trevino and Victor, 1992) or the group would benefit from whistleblowing (Victor et al., 1993). The threat of being confronted by team members, as a consequence of whistleblowing, was negatively associated with whistleblowing intentions (Kaplan et al., 2010; Olesen et al., 2019). When groups within an organization approved whistleblowing, intentions to blow the whistle on a wrongdoing were higher (Kennett et al., 2011), whereas when a group disagreed with whistleblowing, intentions were lower (Miceli and Near, 2002).

#### Support

Support contained the lower-order themes of social support, supportive communication among peers, receptivity, support during previous whistleblowing. Social support was examined across 11 studies (Sims and Keenan, 1998; Milliken et al., 2003; Bellefontaine, 2009; Grube et al., 2010; Kaptein, 2011; Kamarunzaman et al., 2014; Cho and Song, 2015; Chen et al., 2017; Alleyne et al., 2018; Latan et al., 2018; Tumuramye et al., 2018) and was positively linked to whistleblowing intentions. Supportive communication from peers was positively associated with whistleblowing intentions in two studies (Zhang, 2008; Lyndon et al., 2012), but had no effect on whistleblowing intentions in another study (Park et al., 2009).

#### Norms

Cultural norms were associated with whistleblowing intentions in four studies (Schultz et al., 1993; Tan et al., 2003; Alleyne et al., 2017; Pillay et al., 2018), although Gökçe (2013b) found that cultural norms were not associated with whistleblowing intentions. Descriptive norms (Chen et al., 2017), social norms (i.e., the accepted standards of behavior within social groups; Izraeli and Jaffe, 1998; Clements and Shawver, 2011; Shawver, 2011b; Chen and Lai, 2014; Latan et al., 2018), and subjective norms (i.e., the belief about whether an important person or group will approve a particular behavior; Ellis and Arieli, 1999; Park and Blenkinsopp, 2009; Richardson et al., 2012; Trongmateerut and Sweeney, 2013) were positively associated with whistleblowing intentions.

## Discussion

The purpose of this systematic review was to identify the factors that were associated with whistleblowing intentions. We identified 8 dimensions, 26 higher-order themes, and 119 lower-order themes. The whistleblowing dimensions were personal factors, organizational factors, cost and benefits, outcome expectancies, the offense, reporting, the wrongdoer, and social factors. The findings of this systematic review indicate that a person's intentions to blow this whistle is multifaceted. Some findings within the different dimensions, such as personal factors were equivocal, whereas other dimensions were unequivocal such as costs and benefits and the offense.

Personal factors appear important in whether individuals intend to blow the whistle or not. There were, however, some contrasting findings within this dimension among studies that explored the same constructs. In particular, of the 12 studies that examined gender differences five studies found that females were more likely to blow the whistle, whereas seven reported that males are more likely to blow the whistle. One factor that appears to influence this finding is the reporting mechanism, as females prefer anonymized platforms to blow the whistle (Kaplan et al., 2009a). A possible explanation for why females prefer anonymized platforms is because they perceive higher levels of retaliation than males (Rehg et al., 2008). Additionally, power or status in an organization seems to reduce the incidence of retaliation for males, but not for females (Rehg et al., 2008).

In regard to the mixed findings regarding gender, this might be due to the different environments and organizations in which whistleblowing was assessed. Evidence suggests that organizations have different practices and policies on whistleblowing, which are linked to intentions (Olesen et al., 2019). Overall, though, these mixed findings indicate that gender might not be as important as other personal factors that are associated with intentions to blow the whistle.

One explanation for these contrasting findings might relate to moral intensity (Jones, 1991), which was overlooked in the aforementioned studies. Moral intensity has been shown to impact decision making across many different situations, including whistleblowing (Fredin et al., 2018). Moral intensity is composed of six-issue related characteristics (Singer et al., 1998; Shafer et al., 2001). These are the consequences of a moral act (i.e., blowing the whistle), social consensus regarding whether blowing the whistle is ethical or unethical, probability of whether others might blow the whistle, the length of time between blowing the whistle and action occurring, how close the potential whistleblower feels to the victim, and the number of people that would be affected by whistleblowing. Another explanation for these differences may also be the extent to which some people engage in moral disengagement and thus reducing cognitive dissonance by distorting the consequences of wrongdoing or dehumanizing victims, so that their decision not to report an incident is less immoral (Bandura, 1990; Bandura et al., 1996). Future research could consider the role of moral intensity and moral disengagement in influencing whether or not individuals will blow the whistle on wrongdoing or remain silent.

A person's position in an organization was another factor that was associated with whistleblowing intentions, with the majority of studies reporting that those within lower management are more likely to blow the whistle than senior management (e.g., Miceli and Near, 1984; Rothwell and Baldwin, 2006, 2007a,b). This might be because those in lower management are typically newer to their role and those with <2 years in their role are more likely to blow the whistle (Ford, 2013). Recently, however, there has been an increase in regulatory focus among management, so that blowing the whistle on wrongdoing is increasingly being placed on the agenda for all levels of management (Jones and Chiu, 2020), which may explain why a minority of studies found that senior managers were more likely to blow the whistle than middle managers (Brown et al., 2016).

There were a number of constructs within the personal dimension that were positively associated with whistleblowing intentions, which included morality (Latan et al., 2019a,b,c), attitudes (Alleyne et al., 2019), honesty (Keil et al., 2007), and self-efficacy (MacNab and Worthley, 2007). Given that interventions have shown that attitudes (e.g., Nicholls et al., 2020a,b) and self-efficacy (Hyde et al., 2008) can be enhanced, programs that attempt to promote favorable attitudes to whistleblowing and self-efficacy, may have a positive impact on promoting whistleblowing intentions. Research is required to test this assertion.

The organization in which a person belongs to, how it is run, its moral code, and what protection it offers to individuals also appears to be linked to whistleblowing intentions. The size of an organization (Liu and Ren, 2017), unionization (Barnett, 1992), and a hierarchical structure (Satalkar and Shaw, 2018) were all negatively associated with whistleblowing intentions. Leadership is an important organizational factor that is related to whether individuals intend to blow the whistle or not. In particular, ethical leadership within an organization is positively associated with whistleblowing intentions (e.g., Alleyne, 2016; Zhang et al., 2016), along with leadership styles, such as transformational leadership (Caillier, 2013). Linked to leadership, is how leaders within an organization ensure that they offer protection to those who may blow the whistle through handling reports of whistleblowing (Scheetz and Fogarty, 2019) and ensuring that there are policies and practices in place to protect individuals (Olesen et al., 2019). The explanation of Olesen et al.'s (2019) could lie in Siegrist et al.'s (2012) effort-reward imbalance model. Siegrist (1996) proposed that decision making is based on the appraisal of effort and reward. Even though an individual may be motivated to blow the whistle on wrongdoing, if he or she appraises that the reward whistleblowing is low or indeed negative (e.g., job loss, retaliation, or impeding one's chance or promotion), he or she may remain quiet. The effort-reward model represents an interesting framework for future whistleblowing research and is something that leaders could consider when developing whistleblowing mechanisms to protect individuals.

Organizational leadership is also responsible for setting out an organization's moral code, in regard to ethics, values, and working climate. The most ethical organizations had staff with stronger whistleblowing intentions (Aydan and Kaya, 2018; Taylor and Curtis, 2018), as did those with highly ethical working climates for staff (Ahmad et al., 2014). One of the reasons why leadership may be associated with whistleblowing intentions is because it is they who are responsible for monitoring organizational governance and ensuring compliance (D'Cruz and Bjørkelo, 2016) and also creating the environment in which bullying does not take place, so that workers do not fear reprisals (Bjorkelo et al., 2020). Another possible reason is that ethical leaders create a highly ethical culture in which wrongdoing strongly deviates from the norm. In such environments, individuals may have stronger whistleblowing intentions.

With respect to other dimensions that are associated with whistleblowing intentions, the studies on cost and benefit dimensions revealed that monetary losses were negatively associated with intentions to blow the whistle (e.g., Cho and Song, 2015). Monetary gains, however, were positively associated with whistleblowing intentions (Andon et al., 2018). Larger monetary rewards were linked to stronger whistleblowing intentions than smaller amounts (Berger et al., 2017).

Somewhat linked to cost and benefit dimension, was the outcome expectancies dimension, in which individuals considered the impact of whistleblowing on their organization and themselves. If people expected their career development to suffer after blowing the whistle, they were less likely to do so (Fleming et al., 2018). Further, people were less intent on blowing the whistle if they felt this act would damage their organization (Hwang et al., 2014). The offense dimension contained studies that indicated that severity (Richardson et al., 2012), type (Brink et al., 2017), and frequency of wrongdoing (Lyndon et al., 2012) were positively associated with whistleblowing intentions.

The type of reporting mechanism available to individuals to blow the whistle impacted intentions in some studies, but the findings were mixed. Indeed, anonymized reporting channels were associated with higher whistleblowing intentions, in comparison to non-anonymized reporting (Alleyne et al., 2017). It should be noted, however, that when anonymized channels were compared with protective identity systems there was no difference (Curtis and Taylor, 2009). There is conflicting evidence between whistleblowing intentions and reporting channels that are internally managed in comparison to externally managed channels, so this might not be as important as other factors (Gao et al., 2015).

The person that has committed the wrongdoing and his or her relationship with the potential whistleblower is another antecedent of whistleblowing intention. Whistleblowing intentions were weaker when the wrongdoer was a superior within the organization (Chaudhary et al., 2019) and when the wrongdoer was closer to the person observing the wrongdoing (Olesen et al., 2019).

In the social dimension, people had stronger whistleblowing intention when there was strong group cohesion (Latan et al., 2018), or if group members were affected by wrongdoing (Trevino and Victor, 1992). Finally, cultural (Pillay et al., 2018), social (Chen and Lai, 2014), and subjective factors (Richardson et al., 2012) were positively associated with whistleblowing intentions.

It should be noted that most of the studies included in this systematic review examined participants' intentions to blow the whistle in scenarios in which wrongdoing occurred among doctors and nurses (Lyndon et al., 2012), soldiers (Ellis and Arieli, 1999), accountants (Alleyne et al., 2019), and accounting students (Trongmateerut and Sweeney, 2013). In contrast, other studies reported intentions to report wrongdoing for behaviors that had actually occurred (e.g., Moore and McAuliffe, 2010, 2012). Despite differences in the occupations of the participants recruited and whether situation had occurred or was a scenario-based study, these factors did not appear to impact on the results. That is, there were many commonalities among scenario based and studies that involved actual behavior, in addition to individuals with different occupations.

### Limitations

According to the most recent PRISMA guidelines (Page et al., 2021), it is good practice to compare the findings of a systematic review with previous systematic reviews. As previously mentioned, this is the first systematic review on whistleblowing across multiple domains such as medicine, heath, finance, and government, so it is not possible to do this in the present review. A consequence of this is that the studies included in this systematic review varied greatly, along with the type of wrongdoing, such as cheating, food tampering, stealing money, and endangering the lives of patients.

The majority of studies reported whistleblowing intentions in hypothetical scenarios, and only a few of the studies reported intentions among individuals who blew this whistle. It should be noted, however, that including studies across these different domains and in both real-life and hypothetical situations allows scholars to full grasp the antecedents of whistleblowing intentions, which may be useful for developing interventions to promote whistleblowing behaviors.

Another limitation of this study relates to the review processes used. Due to time constraints, all searches were screened by one author, Lucas Fairs. Recent evidence suggests that single screening of abstracts misses up to 13% of studies that may be deemed relevant (Gartlehner et al., 2020). It should be noted however, that all decisions were verified by the lead author, Adam Nicholls, who checked the data. As such, there is the possibility of some risk of error within this systematic review, but others such as Nussbaumer-Streit et al. (2020) suggested that single author screening of abstracts would not change the overall conclusions of a review. As such, we feel confident that the systematic review is representative of the literature and that our conclusions are valid.

Another limitation relates to comparisons made in regard to whistleblowing intentions and country of residence. Most studies compared whistleblowing intentions between U.S citizens and citizens from another country. As such, generalizations cannot be made to people who reside in other countries.

### Implication for Practice, Future Research, and Policy

Given the importance of whistleblowing in revealing wrongdoing (e.g., Whitaker et al., 2014; Blenkinsopp et al., 2019; Mehrotra et al., 2020), it is important that most, if not all, employees or members from an organization have strong intentions to report any wrongdoing they encounter. Although some of the findings from this systematic review were equivocal, there appears to be a number of steps that organizations can take to maximize the likelihood of individuals blowing the whistle on wrongdoing. Given the apparent multifaceted nature of whistleblowing, a multipronged approach may be required to maximize whistleblowing intentions, which stems from the leadership and management of an organization to empower, educate, protect, support, and reward those who blow the whistle. Indeed, the way in which an organization is managed in terms of ethics and leadership styles, is crucial for enhancing whistleblowing intentions (Nurhidayat and Kusumasari, 2019).

Providing education on whistleblowing to individuals within an organization, such as explaining what whistleblowing is, why it is important, how to do it, and place a personal responsibility on individuals to blow the whistle when they see wrongdoing, rather than waiting for someone else to act (Alleyne et al., 2017) may also be important. Indeed, a recent European Barometer revealed that 49% of citizens did not know how or where to report corruption (European Commission, 2018). Organizations should value and promote whistleblowing to employees or members, given this is also linked to intentions to blow the whistle (Pillay et al., 2012).

Organizations need to have procedures in place that offer genuine protection, in terms of reporting procedures that protect the identity of whistleblowers (Pope and Lee, 2013), prevent reprisals and intimidation (Lyndon et al., 2012), and do not harm future career development (Liu and Ren, 2017; Fleming et al., 2018). The threat of retaliation may explain why 81% of respondents failed to report corruption in a recent EU Special Barometer on corruption (European Commission, 2018). Leaders within organizations need to support individuals by positively promoting whistleblowing to employees or members, given people are less likely to blow the whistle if they fear a manager will have a negative reaction (Scheetz and Fogarty, 2019) or they have poor communication with their manager (Chaudhary et al., 2019). As such, individuals need to feel that they would be supported by the management if they blew the whistle on wrongdoing.

Another way to promote whistleblowing is to reward whistleblowers financially (Latan et al., 2019a,b,c) or offer reduced sentences, so that the benefits of whistleblowing outweigh the costs (Keil et al., 2010). It should be noted that these incentives may encourage false whistleblowing accounts, so caution might be needed (Mattiesen et al., 2011).

Future research could assess the recommendations made in this systematic review by assessing their impact on whistleblowing intentions and modify them accordingly and longitudinally tracking the extent to which individuals report any wrongdoing they encounter. It would also be interesting to examine whether interventions that promote whistleblowing intentions could be generic or whether they need to be context specific. That is, would a generic whistleblowing intervention be just as effective as an intervention designed specifically for doctors, nurses, accountants, or teachers. Our findings would indicate that key factors to promote whistleblowing intentions are unlikely to be context specific. However, this assumption would need testing.

There are a number of personal factors that are associated with whistleblowing intentions such as attitudes, self-efficacy, and confidence. It would be useful to see, for example, how promoting favorable attitudes toward blowing the whistle may shape whistleblowing intentions. If promoting favorable attitudes impacts whistleblowing intentions, this could be incorporated within educational interventions too. Given the importance of whistleblowing on revealing wrongdoing, developing interventions appears to be an important step in this area of research.

In regard to whistleblowing policy, the protection for whistleblowers varies across different countries. Within the European Union, for example, only 10 countries (e.g., France, Hungary, Ireland, Italy, Lithuania, Malta, Netherlands, Slovakia, Sweden, and the UK) provide comprehensive legal protection to whistleblowers from reprisal (European Commission, 2018). However, the European Union launched a new directive to provide comprehensive and uniform protection to all whistleblowers (Directive (EU) 2019/1937, 2019), which means that companies with more 50 employees are obliged to set up suitable internal reporting channels. A key feature of this directive is to provide protection to employees, job applicants, former employees, supporters of a whistleblower, and journalists who report wrongdoing. Currently, protection only applies to wrongdoing relating to EU law (e.g., tax fraud, money laundering, public health, product and road safety, environmental protection, consumer protection, and data protection).

Similarly, there have also been changes in the United States (US) to protect whistleblowers, such as the Dodd-Frank Act (2010), which was passed in following the financial crisis and offers protection and rewards to whistleblowers who report wrongdoing. From 2011 to 2020, the U.S. Securities and Exchange Commission (SEC) and the Commodity Futures Trading Commission (CFTC) have recovered over $3.7 billion. Further, $840 million has been awarded to whistleblowers (National Whistleblower Centre, 2021). Another example of changes in US law is the Presidential Policy Directive-19 (2019) which protects whistleblowers with classified information. In the United States there are over 20 statutes to protect whistleblowers (Occupational Safety and Health Administration, 2019) and, US citizens living in the US and outside of the US are offered protection when reporting wrongdoing (National Whistleblower Centre, 2021).

Intentions to blow the whistle are related to a multitude of factors, and one of these identified in this systematic review is outcome expectancies. If individuals do not feel threatened but feel protected, they are likely to have stronger whistleblowing intentions (Cho and Song, 2015). As such, the new directives and laws within the US and the EU are positive steps. However, whistleblowers who report wrongdoing outside EU laws, but relate to the breaking on national laws among member states are not currently protected by the EU. As such, there will be some circumstances in which whistleblowers are not offered comprehensive protection, even when individuals are reporting wrongdoing that breaks national laws.

In conclusion, whistleblowing intentions are multifaceted. They can be associated with personal factors, organizational factors, cost and benefits appraisals, outcome expectancies, the offense, reporting, the wrongdoer, and social factors. Based on the findings, it is apparent that organizations should educate their employees, protect their employees from reprisals, support those who blow the whistle on wrongdoing, and even reward whistleblowers. This should empower individuals to blow the whistle and thus increase the likelihood of individuals blowing the whistle on corruption and wrongdoing. Further, education on whistleblowing could also include psycho-social components that promote favorable attitudes toward whistleblowing, boost self-efficacy, and improve communication between employees and management. From a policy perspective, more consistent protection is required across different countries, so that organizations are required to adhere to laws and policies. Taken together, these factors could increase whistleblowing intentions, although research is required to assess these recommendations.

## Data Availability Statement

The original contributions presented in the study are included in the article/Supplementary Material, further inquiries can be directed to the corresponding author/s.

## Author Contributions

All authors listed have made a substantial, direct and intellectual contribution to the work, and approved it for publication.

## Funding

This systematic review was funded by the European Commission's Education, Audiovisual and Culture Executive Agency [Unit A.6, Erasmus +: Sport, Youth and EU Aid Volunteers]. Project title: Understanding and promoting whistleblowing on doping irregularities in the EU (Win-Dop). Project reference number: 612968-EPP-1-2019-1-UK-SPO-SCP.

## Conflict of Interest

The authors declare that the research was conducted in the absence of any commercial or financial relationships that could be construed as a potential conflict of interest.

## Publisher's Note

All claims expressed in this article are solely those of the authors and do not necessarily represent those of their affiliated organizations, or those of the publisher, the editors and the reviewers. Any product that may be evaluated in this article, or claim that may be made by its manufacturer, is not guaranteed or endorsed by the publisher.
