# Supplementary material for: Snitches Get Stitches and End Up in Ditches: A Systematic Review of the Factors Associated With Whistleblowing Intentions
Source: Front Psychol. 2021 Oct 5;12:631538. doi: 10.3389/fpsyg.2021.631538 (PMC8523783; doi:10.3389/fpsyg.2021.631538)
Supplement: Supplementary file 1 [file Data_Sheet_1.docx]

Supplementary Table 1: Risk of Bias

| **Authors and Year** | **1** | **2** | **3** | **4** | **5** | **6** | **7** | **8** | **9** | **10** | **11** | **12** | **13** | **14** | **15** |
| --- | --- | --- | --- | --- | --- | --- | --- | --- | --- | --- | --- | --- | --- | --- | --- |
| Ahern and McDonald (2002) |  |  |  |  |  |  |  |  |  |  |  |  |  |  |  |
| Ahmad et al. (2013) |  |  |  |  |  |  |  |  |  |  |  |  |  |  |  |
| Ahmad et al. (2014) |  |  |  |  |  |  |  |  |  |  |  |  |  |  |  |
| Alinaghian et al. (2018) |  |  |  |  |  |  |  |  |  |  |  |  |  |  |  |
| Alleyne (2016) |  |  |  |  |  |  |  |  |  |  |  |  |  |  |  |
| Alleyne et al. (2017) |  |  |  |  |  |  |  |  |  |  |  |  |  |  |  |
| Alleyne et al. (2019) |  |  |  |  |  |  |  |  |  |  |  |  |  |  |  |
| Alleyne et al. (2018) |  |  |  |  |  |  |  |  |  |  |  |  |  |  |  |
| Alleyne et al. (2013) |  |  |  |  |  |  |  |  |  |  |  |  |  |  |  |
| Andon et al. (2018) |  |  |  |  |  |  |  |  |  |  |  |  |  |  |  |
| Arifah et al. (2017) |  |  |  |  |  |  |  |  |  |  |  |  |  |  |  |
| Atkinson et al. (2012) |  |  |  |  |  |  |  |  |  |  |  |  |  |  |  |
| Attree (2007) |  |  |  |  |  |  |  |  |  |  |  |  |  |  |  |
| Aydan and Kaya (2018) |  |  |  |  |  |  |  |  |  |  |  |  |  |  |  |
| Ayers and Kaplan (2005) |  |  |  |  |  |  |  |  |  |  |  |  |  |  |  |
| Barnett (1992) |  |  |  |  |  |  |  |  |  |  |  |  |  |  |  |
| Barnett et al. (1996) |  |  |  |  |  |  |  |  |  |  |  |  |  |  |  |
| Barnett et al. (1993) |  |  |  |  |  |  |  |  |  |  |  |  |  |  |  |
| Bellefontaine (2009) |  |  |  |  |  |  |  |  |  |  |  |  |  |  |  |
| Berger et al. (2017) |  |  |  |  |  |  |  |  |  |  |  |  |  |  |  |
| Bhal and Dadhich (2011) |  |  |  |  |  |  |  |  |  |  |  |  |  |  |  |
| Binikos (2008) |  |  |  |  |  |  |  |  |  |  |  |  |  |  |  |
| Bjørkelo et al. (2010) |  |  |  |  |  |  |  |  |  |  |  |  |  |  |  |
| Black (2011) |  |  |  |  |  |  |  |  |  |  |  |  |  |  |  |
| Bocchiaro et al. (2012) |  |  |  |  |  |  |  |  |  |  |  |  |  |  |  |
| Boo et al. (2016) |  |  |  |  |  |  |  |  |  |  |  |  |  |  |  |
| Brabeck (1984) |  |  |  |  |  |  |  |  |  |  |  |  |  |  |  |
| Brennan and Kelly (2007) |  |  |  |  |  |  |  |  |  |  |  |  |  |  |  |
| Brink et al. (2017) |  |  |  |  |  |  |  |  |  |  |  |  |  |  |  |
| Brink et al. (2013) |  |  |  |  |  |  |  |  |  |  |  |  |  |  |  |
| Brooks and Perot (1991) |  |  |  |  |  |  |  |  |  |  |  |  |  |  |  |
| Brown et al. (2016) |  |  |  |  |  |  |  |  |  |  |  |  |  |  |  |
| Caillier (2013) |  |  |  |  |  |  |  |  |  |  |  |  |  |  |  |
| Caillier (2017a) |  |  |  |  |  |  |  |  |  |  |  |  |  |  |  |
| Caillier (2017b) |  |  |  |  |  |  |  |  |  |  |  |  |  |  |  |
| Casal and Bogui (2008) |  |  |  |  |  |  |  |  |  |  |  |  |  |  |  |
| Cassematis and Wortley (2013) |  |  |  |  |  |  |  |  |  |  |  |  |  |  |  |
| Chaudhary et al. (2019) |  |  |  |  |  |  |  |  |  |  |  |  |  |  |  |
| Chen and Lai (2014) |  |  |  |  |  |  |  |  |  |  |  |  |  |  |  |
| Chen et al. (2017) |  |  |  |  |  |  |  |  |  |  |  |  |  |  |  |
| Cheng et al. (2019) |  |  |  |  |  |  |  |  |  |  |  |  |  |  |  |
| Chiasson et al. (1995) |  |  |  |  |  |  |  |  |  |  |  |  |  |  |  |
| Chiu (2002) |  |  |  |  |  |  |  |  |  |  |  |  |  |  |  |
| Chiu (2003) |  |  |  |  |  |  |  |  |  |  |  |  |  |  |  |
| Chiu and Erdener (2003) |  |  |  |  |  |  |  |  |  |  |  |  |  |  |  |
| Cho and Song (2015) |  |  |  |  |  |  |  |  |  |  |  |  |  |  |  |
| Clements and Shawver (2011) |  |  |  |  |  |  |  |  |  |  |  |  |  |  |  |
| Clements and Shawver (2009) |  |  |  |  |  |  |  |  |  |  |  |  |  |  |  |
| Curtis (2006) |  |  |  |  |  |  |  |  |  |  |  |  |  |  |  |
| Curtis and Taylor (2009) |  |  |  |  |  |  |  |  |  |  |  |  |  |  |  |
| Dalton and Radtke (2013) |  |  |  |  |  |  |  |  |  |  |  |  |  |  |  |
| Elias (2008) |  |  |  |  |  |  |  |  |  |  |  |  |  |  |  |
| Ellis and Arieli (1999) |  |  |  |  |  |  |  |  |  |  |  |  |  |  |  |
| Erickson et al. (2017) |  |  |  |  |  |  |  |  |  |  |  |  |  |  |  |
| Erkmen et al. (2014) |  |  |  |  |  |  |  |  |  |  |  |  |  |  |  |
| Erturk and Donmez (2016) |  |  |  |  |  |  |  |  |  |  |  |  |  |  |  |
| Fieger and Rice (2018) |  |  |  |  |  |  |  |  |  |  |  |  |  |  |  |
| Fleming et al. (2018) |  |  |  |  |  |  |  |  |  |  |  |  |  |  |  |
| Francalanza and Buttigieg (2016) |  |  |  |  |  |  |  |  |  |  |  |  |  |  |  |
| Fredin et al. (2019) |  |  |  |  |  |  |  |  |  |  |  |  |  |  |  |
| Fredin (2011) |  |  |  |  |  |  |  |  |  |  |  |  |  |  |  |
| Gao et al. (2015) |  |  |  |  |  |  |  |  |  |  |  |  |  |  |  |
| Grube et al. (2010) |  |  |  |  |  |  |  |  |  |  |  |  |  |  |  |
| Gökçe (2013a) |  |  |  |  |  |  |  |  |  |  |  |  |  |  |  |
| Gökçe (2013b) |  |  |  |  |  |  |  |  |  |  |  |  |  |  |  |
| Gökçe (2013c) |  |  |  |  |  |  |  |  |  |  |  |  |  |  |  |
| Gökçe (2013d) |  |  |  |  |  |  |  |  |  |  |  |  |  |  |  |
| Gökçe (2013e) |  |  |  |  |  |  |  |  |  |  |  |  |  |  |  |
| Gökçe (2015) |  |  |  |  |  |  |  |  |  |  |  |  |  |  |  |
| Gundlach et al. (2008) |  |  |  |  |  |  |  |  |  |  |  |  |  |  |  |
| Guthrie and Taylor (2017) |  |  |  |  |  |  |  |  |  |  |  |  |  |  |  |
| Hechanova and Manaois (2020) |  |  |  |  |  |  |  |  |  |  |  |  |  |  |  |
| Henningsen et al. (2013) |  |  |  |  |  |  |  |  |  |  |  |  |  |  |  |
| Hwang et al. (2008) |  |  |  |  |  |  |  |  |  |  |  |  |  |  |  |
| Hwang et al. (2014) |  |  |  |  |  |  |  |  |  |  |  |  |  |  |  |
| Ion et al. (2015) |  |  |  |  |  |  |  |  |  |  |  |  |  |  |  |
| Ion et al. (2016) |  |  |  |  |  |  |  |  |  |  |  |  |  |  |  |
| Izraeli and Jaffe (1998) |  |  |  |  |  |  |  |  |  |  |  |  |  |  |  |
| Jackson et al. (2010) |  |  |  |  |  |  |  |  |  |  |  |  |  |  |  |
| Jenkel and Haen (2012) |  |  |  |  |  |  |  |  |  |  |  |  |  |  |  |
| Jones et al. (2014) |  |  |  |  |  |  |  |  |  |  |  |  |  |  |  |
| Kamarunzaman et al. (2014) |  |  |  |  |  |  |  |  |  |  |  |  |  |  |  |
| Kaplan et al. (2009) |  |  |  |  |  |  |  |  |  |  |  |  |  |  |  |
| Kaplan (1995) |  |  |  |  |  |  |  |  |  |  |  |  |  |  |  |
| Kaplan et al. (2009) |  |  |  |  |  |  |  |  |  |  |  |  |  |  |  |
| Kaplan et al. (2010) |  |  |  |  |  |  |  |  |  |  |  |  |  |  |  |
| Kaplan et al. (2011) |  |  |  |  |  |  |  |  |  |  |  |  |  |  |  |
| Kaplan and Schultz (2007) |  |  |  |  |  |  |  |  |  |  |  |  |  |  |  |
| Kaplan and Whitecotton (2001) |  |  |  |  |  |  |  |  |  |  |  |  |  |  |  |
| Kaptein (2011) |  |  |  |  |  |  |  |  |  |  |  |  |  |  |  |
| Keenan (1995) |  |  |  |  |  |  |  |  |  |  |  |  |  |  |  |
| Keenan (2000) |  |  |  |  |  |  |  |  |  |  |  |  |  |  |  |
| Keenan (2002a) |  |  |  |  |  |  |  |  |  |  |  |  |  |  |  |
| Keenan (2002b) |  |  |  |  |  |  |  |  |  |  |  |  |  |  |  |
| Keenan (2007) |  |  |  |  |  |  |  |  |  |  |  |  |  |  |  |
| Keil et al. (2007) |  |  |  |  |  |  |  |  |  |  |  |  |  |  |  |
| Keil et al. (2018) |  |  |  |  |  |  |  |  |  |  |  |  |  |  |  |
| Keil et al. (2004) |  |  |  |  |  |  |  |  |  |  |  |  |  |  |  |
| Keil et al. (2010) |  |  |  |  |  |  |  |  |  |  |  |  |  |  |  |
| Kennett et al. (2011) |  |  |  |  |  |  |  |  |  |  |  |  |  |  |  |
| King (1997) |  |  |  |  |  |  |  |  |  |  |  |  |  |  |  |
| King and Hermodson (2000) |  |  |  |  |  |  |  |  |  |  |  |  |  |  |  |
| King and Scudder (2013) |  |  |  |  |  |  |  |  |  |  |  |  |  |  |  |
| Latan et al. (2019a) |  |  |  |  |  |  |  |  |  |  |  |  |  |  |  |
| Latan et al. (2019b) |  |  |  |  |  |  |  |  |  |  |  |  |  |  |  |
| Latan et al. (2019c) |  |  |  |  |  |  |  |  |  |  |  |  |  |  |  |
| Latan et al. (2018) |  |  |  |  |  |  |  |  |  |  |  |  |  |  |  |
| Lavena (2016) |  |  |  |  |  |  |  |  |  |  |  |  |  |  |  |
| Lee et al. (2004) |  |  |  |  |  |  |  |  |  |  |  |  |  |  |  |
| Li and Ma (2016) |  |  |  |  |  |  |  |  |  |  |  |  |  |  |  |
| Lim and See (2001) |  |  |  |  |  |  |  |  |  |  |  |  |  |  |  |
| Liu and Ren (2017) |  |  |  |  |  |  |  |  |  |  |  |  |  |  |  |
| Liu et al. (2015) |  |  |  |  |  |  |  |  |  |  |  |  |  |  |  |
| Liu et al. (2016) |  |  |  |  |  |  |  |  |  |  |  |  |  |  |  |
| Liyanarachchi and Newdick (2009) |  |  |  |  |  |  |  |  |  |  |  |  |  |  |  |
| Liyanarachchi and Adler (2011) |  |  |  |  |  |  |  |  |  |  |  |  |  |  |  |
| Lowe et al. (2015) |  |  |  |  |  |  |  |  |  |  |  |  |  |  |  |
| Lyndon et al. (2012) |  |  |  |  |  |  |  |  |  |  |  |  |  |  |  |
| MacGregor and Stuebs (2014) |  |  |  |  |  |  |  |  |  |  |  |  |  |  |  |
| MacNab et al. (2007) |  |  |  |  |  |  |  |  |  |  |  |  |  |  |  |
| MacNab and Worthley (2008) |  |  |  |  |  |  |  |  |  |  |  |  |  |  |  |
| Mbago et al. (2018) |  |  |  |  |  |  |  |  |  |  |  |  |  |  |  |
| McCutcheon (2006) |  |  |  |  |  |  |  |  |  |  |  |  |  |  |  |
| McManus et al. (2012) |  |  |  |  |  |  |  |  |  |  |  |  |  |  |  |
| Miceli et al. (1991) |  |  |  |  |  |  |  |  |  |  |  |  |  |  |  |
| Miceli et al. (2012) |  |  |  |  |  |  |  |  |  |  |  |  |  |  |  |
| Miceli et al. (1991) |  |  |  |  |  |  |  |  |  |  |  |  |  |  |  |
| Miceli and Near (1984) |  |  |  |  |  |  |  |  |  |  |  |  |  |  |  |
| Miceli and Near (1985) |  |  |  |  |  |  |  |  |  |  |  |  |  |  |  |
| Miceli and Near (1988) |  |  |  |  |  |  |  |  |  |  |  |  |  |  |  |
| Milliken et al. (2003) |  |  |  |  |  |  |  |  |  |  |  |  |  |  |  |
| Moore and McAuliffe (2010) |  |  |  |  |  |  |  |  |  |  |  |  |  |  |  |
| Moore and McAuliffe (2012) |  |  |  |  |  |  |  |  |  |  |  |  |  |  |  |
| Nawawi and Salin (2018) |  |  |  |  |  |  |  |  |  |  |  |  |  |  |  |
| Nayir et al. (2018) |  |  |  |  |  |  |  |  |  |  |  |  |  |  |  |
| Near et al. (1993) |  |  |  |  |  |  |  |  |  |  |  |  |  |  |  |
| Near et al. (2004) |  |  |  |  |  |  |  |  |  |  |  |  |  |  |  |
| Nurhidayat and Kusumasari (2019) |  |  |  |  |  |  |  |  |  |  |  |  |  |  |  |
| Oelrich (2019) |  |  |  |  |  |  |  |  |  |  |  |  |  |  |  |
| Olesen et al. (2019) |  |  |  |  |  |  |  |  |  |  |  |  |  |  |  |
| Omotoye (2017) |  |  |  |  |  |  |  |  |  |  |  |  |  |  |  |
| Park et al. (2008) |  |  |  |  |  |  |  |  |  |  |  |  |  |  |  |
| Park and Keil (2009) |  |  |  |  |  |  |  |  |  |  |  |  |  |  |  |
| Park et al. (2009) |  |  |  |  |  |  |  |  |  |  |  |  |  |  |  |
| Park and Blenkinsopp (2009) |  |  |  |  |  |  |  |  |  |  |  |  |  |  |  |
| Park et al. (2005) |  |  |  |  |  |  |  |  |  |  |  |  |  |  |  |
| Perry et al. (1997) |  |  |  |  |  |  |  |  |  |  |  |  |  |  |  |
| Pillay et al. (2012) |  |  |  |  |  |  |  |  |  |  |  |  |  |  |  |
| Pillay et al. (2018) |  |  |  |  |  |  |  |  |  |  |  |  |  |  |  |
| Pillay et al. (2017) |  |  |  |  |  |  |  |  |  |  |  |  |  |  |  |
| Pope and Lee (2013) |  |  |  |  |  |  |  |  |  |  |  |  |  |  |  |
| Previtali and Cerchiello (2018) |  |  |  |  |  |  |  |  |  |  |  |  |  |  |  |
| Proost et al. (2013) |  |  |  |  |  |  |  |  |  |  |  |  |  |  |  |
| Radulovic and Uys (2019) |  |  |  |  |  |  |  |  |  |  |  |  |  |  |  |
| Reckers-Sauciuc and Lowe (2010) |  |  |  |  |  |  |  |  |  |  |  |  |  |  |  |
| Rennie and Crosby (2002) |  |  |  |  |  |  |  |  |  |  |  |  |  |  |  |
| Richardson et al. (2012) |  |  |  |  |  |  |  |  |  |  |  |  |  |  |  |
| Richardson et al. (2008) |  |  |  |  |  |  |  |  |  |  |  |  |  |  |  |
| Robertson et al. (2011) |  |  |  |  |  |  |  |  |  |  |  |  |  |  |  |
| Robinson et al. (2012) |  |  |  |  |  |  |  |  |  |  |  |  |  |  |  |
| Rose et al. (2018) |  |  |  |  |  |  |  |  |  |  |  |  |  |  |  |
| Rothwell and Baldwin (2006) |  |  |  |  |  |  |  |  |  |  |  |  |  |  |  |
| Rothwell and Baldwin (2007a) |  |  |  |  |  |  |  |  |  |  |  |  |  |  |  |
| Rothwell and Baldwin (2007b) |  |  |  |  |  |  |  |  |  |  |  |  |  |  |  |
| Rustiarini and Sunarsih (2017) |  |  |  |  |  |  |  |  |  |  |  |  |  |  |  |
| Satalkar and Shaw (2018) |  |  |  |  |  |  |  |  |  |  |  |  |  |  |  |
| Scheetz and Fogarty (2019) |  |  |  |  |  |  |  |  |  |  |  |  |  |  |  |
| Scheetz and Wilson (2019) |  |  |  |  |  |  |  |  |  |  |  |  |  |  |  |
| Schultz et al. (1993) |  |  |  |  |  |  |  |  |  |  |  |  |  |  |  |
| Seifert et al. (2014) |  |  |  |  |  |  |  |  |  |  |  |  |  |  |  |
| Seifert et al. (2010) |  |  |  |  |  |  |  |  |  |  |  |  |  |  |  |
| Shawver (2008) |  |  |  |  |  |  |  |  |  |  |  |  |  |  |  |
| Shawver (2011a) |  |  |  |  |  |  |  |  |  |  |  |  |  |  |  |
| Shawver (2011b) |  |  |  |  |  |  |  |  |  |  |  |  |  |  |  |
| Sims and Keenan (1999) |  |  |  |  |  |  |  |  |  |  |  |  |  |  |  |
| Sims and Keenan (1998) |  |  |  |  |  |  |  |  |  |  |  |  |  |  |  |
| Singer et al. (1998) |  |  |  |  |  |  |  |  |  |  |  |  |  |  |  |
| Smith et al. (2001) |  |  |  |  |  |  |  |  |  |  |  |  |  |  |  |
| Somers and Casal (2011) |  |  |  |  |  |  |  |  |  |  |  |  |  |  |  |
| Somers and Casal (1994) |  |  |  |  |  |  |  |  |  |  |  |  |  |  |  |
| Soni et al. (2015) |  |  |  |  |  |  |  |  |  |  |  |  |  |  |  |
| Stansbury and Victor (2009) |  |  |  |  |  |  |  |  |  |  |  |  |  |  |  |
| Stikeleather (2016) |  |  |  |  |  |  |  |  |  |  |  |  |  |  |  |
| Stöber et al. (2019) |  |  |  |  |  |  |  |  |  |  |  |  |  |  |  |
| Su et al. (2010) |  |  |  |  |  |  |  |  |  |  |  |  |  |  |  |
| Surya et al. (2017) |  |  |  |  |  |  |  |  |  |  |  |  |  |  |  |
| Tan et al. (2003) |  |  |  |  |  |  |  |  |  |  |  |  |  |  |  |
| Tavakoli et al. (2003) |  |  |  |  |  |  |  |  |  |  |  |  |  |  |  |
| Taylor and Curtis (2010) |  |  |  |  |  |  |  |  |  |  |  |  |  |  |  |
| Taylor and Curtis (2013) |  |  |  |  |  |  |  |  |  |  |  |  |  |  |  |
| Taylor and Curtis (2018) |  |  |  |  |  |  |  |  |  |  |  |  |  |  |  |
| Taylor (2018) |  |  |  |  |  |  |  |  |  |  |  |  |  |  |  |
| Taylor (2019) |  |  |  |  |  |  |  |  |  |  |  |  |  |  |  |
| Teichmann (2019) |  |  |  |  |  |  |  |  |  |  |  |  |  |  |  |
| Thoroughgood et al. (2011) |  |  |  |  |  |  |  |  |  |  |  |  |  |  |  |
| Trevino and Victor (1992) |  |  |  |  |  |  |  |  |  |  |  |  |  |  |  |
| Trongmateerut and Sweeney (2013) |  |  |  |  |  |  |  |  |  |  |  |  |  |  |  |
| Tumuramye et al. (2018) |  |  |  |  |  |  |  |  |  |  |  |  |  |  |  |
| Ugaddan and Park (2019) |  |  |  |  |  |  |  |  |  |  |  |  |  |  |  |
| Victor et al. (1993) |  |  |  |  |  |  |  |  |  |  |  |  |  |  |  |
| Vincent et al. (1999) |  |  |  |  |  |  |  |  |  |  |  |  |  |  |  |
| Wainberg and Perreault (2016) |  |  |  |  |  |  |  |  |  |  |  |  |  |  |  |
| Wen and Chen (2016) |  |  |  |  |  |  |  |  |  |  |  |  |  |  |  |
| Whitaker et al. (2014) |  |  |  |  |  |  |  |  |  |  |  |  |  |  |  |
| Wilson et al. (2018) |  |  |  |  |  |  |  |  |  |  |  |  |  |  |  |
| Xu and Ziegenfuss (2008) |  |  |  |  |  |  |  |  |  |  |  |  |  |  |  |
| Yu et al. (2019) |  |  |  |  |  |  |  |  |  |  |  |  |  |  |  |
| Zarefar and Zarefar (2017) |  |  |  |  |  |  |  |  |  |  |  |  |  |  |  |
| Zhang et al. (2016) |  |  |  |  |  |  |  |  |  |  |  |  |  |  |  |
| Zhang et al. (2013) |  |  |  |  |  |  |  |  |  |  |  |  |  |  |  |
| Zhang et al. (2009a) |  |  |  |  |  |  |  |  |  |  |  |  |  |  |  |
| Zhang et al. (2009b) |  |  |  |  |  |  |  |  |  |  |  |  |  |  |  |
| Zhang (2008) |  |  |  |  |  |  |  |  |  |  |  |  |  |  |  |
| Zheng et al. (2019) |  |  |  |  |  |  |  |  |  |  |  |  |  |  |  |
| Zhou et al. (2018) |  |  |  |  |  |  |  |  |  |  |  |  |  |  |  |
| Zhuang et al. (2005) |  |  |  |  |  |  |  |  |  |  |  |  |  |  |  |
| Zipparo (1999) |  |  |  |  |  |  |  |  |  |  |  |  |  |  |  |
| Authors and Year | 1 | 2 | 3 | 4 | 5 | 6 | 7 | 8 | 9 | 10 | 11 | 12 | 13 | 14 | 15 |
| Ahern and McDonald (2002) |  |  |  |  |  |  |  |  |  |  |  |  |  |  |  |
| Ahmad et al. (2013) |  |  |  |  |  |  |  |  |  |  |  |  |  |  |  |
| Ahmad et al. (2014) |  |  |  |  |  |  |  |  |  |  |  |  |  |  |  |
| Alinaghian et al. (2018) |  |  |  |  |  |  |  |  |  |  |  |  |  |  |  |
| Alleyne (2016) |  |  |  |  |  |  |  |  |  |  |  |  |  |  |  |
| Alleyne et al. (2017) |  |  |  |  |  |  |  |  |  |  |  |  |  |  |  |
| Alleyne et al. (2019) |  |  |  |  |  |  |  |  |  |  |  |  |  |  |  |
| Alleyne et al. (2018) |  |  |  |  |  |  |  |  |  |  |  |  |  |  |  |
| Alleyne et al. (2013) |  |  |  |  |  |  |  |  |  |  |  |  |  |  |  |
| Andon et al. (2018) |  |  |  |  |  |  |  |  |  |  |  |  |  |  |  |
| Arifah et al. (2017) |  |  |  |  |  |  |  |  |  |  |  |  |  |  |  |
| Atkinson et al. (2012) |  |  |  |  |  |  |  |  |  |  |  |  |  |  |  |
| Attree (2007) |  |  |  |  |  |  |  |  |  |  |  |  |  |  |  |
| Aydan and Kaya (2018) |  |  |  |  |  |  |  |  |  |  |  |  |  |  |  |
| Ayers and Kaplan (2005) |  |  |  |  |  |  |  |  |  |  |  |  |  |  |  |
| Barnett (1992) |  |  |  |  |  |  |  |  |  |  |  |  |  |  |  |
| Barnett et al. (1996) |  |  |  |  |  |  |  |  |  |  |  |  |  |  |  |
| Barnett et al. (1993) |  |  |  |  |  |  |  |  |  |  |  |  |  |  |  |
| Bellefontaine (2009) |  |  |  |  |  |  |  |  |  |  |  |  |  |  |  |
| Berger et al. (2017) |  |  |  |  |  |  |  |  |  |  |  |  |  |  |  |
| Bhal and Dadhich (2011) |  |  |  |  |  |  |  |  |  |  |  |  |  |  |  |
| Binikos (2008) |  |  |  |  |  |  |  |  |  |  |  |  |  |  |  |
| Bjørkelo et al. (2010) |  |  |  |  |  |  |  |  |  |  |  |  |  |  |  |
| Black (2011) |  |  |  |  |  |  |  |  |  |  |  |  |  |  |  |
| Bocchiaro et al. (2012) |  |  |  |  |  |  |  |  |  |  |  |  |  |  |  |
| Boo et al. (2016) |  |  |  |  |  |  |  |  |  |  |  |  |  |  |  |
| Brabeck (1984) |  |  |  |  |  |  |  |  |  |  |  |  |  |  |  |
| Brennan and Kelly (2007) |  |  |  |  |  |  |  |  |  |  |  |  |  |  |  |
| Brink et al. (2017) |  |  |  |  |  |  |  |  |  |  |  |  |  |  |  |
| Brink et al. (2013) |  |  |  |  |  |  |  |  |  |  |  |  |  |  |  |
| Brooks and Perot (1991) |  |  |  |  |  |  |  |  |  |  |  |  |  |  |  |
| Brown et al. (2016) |  |  |  |  |  |  |  |  |  |  |  |  |  |  |  |
| Caillier (2013) |  |  |  |  |  |  |  |  |  |  |  |  |  |  |  |
| Caillier (2017a) |  |  |  |  |  |  |  |  |  |  |  |  |  |  |  |
| Caillier (2017b) |  |  |  |  |  |  |  |  |  |  |  |  |  |  |  |
| Casal and Bogui (2008) |  |  |  |  |  |  |  |  |  |  |  |  |  |  |  |
| Cassematis and Wortley (2013) |  |  |  |  |  |  |  |  |  |  |  |  |  |  |  |
| Chaudhary et al. (2019) |  |  |  |  |  |  |  |  |  |  |  |  |  |  |  |
| Chen and Lai (2014) |  |  |  |  |  |  |  |  |  |  |  |  |  |  |  |
| Chen et al. (2017) |  |  |  |  |  |  |  |  |  |  |  |  |  |  |  |
| Cheng et al. (2019) |  |  |  |  |  |  |  |  |  |  |  |  |  |  |  |
| Chiasson et al. (1995) |  |  |  |  |  |  |  |  |  |  |  |  |  |  |  |
| Chiu (2002) |  |  |  |  |  |  |  |  |  |  |  |  |  |  |  |
| Chiu (2003) |  |  |  |  |  |  |  |  |  |  |  |  |  |  |  |
| Chiu and Erdener (2003) |  |  |  |  |  |  |  |  |  |  |  |  |  |  |  |
| Cho and Song (2015) |  |  |  |  |  |  |  |  |  |  |  |  |  |  |  |
| Clements and Shawver (2011) |  |  |  |  |  |  |  |  |  |  |  |  |  |  |  |
| Clements and Shawver (2009) |  |  |  |  |  |  |  |  |  |  |  |  |  |  |  |
| Curtis (2006) |  |  |  |  |  |  |  |  |  |  |  |  |  |  |  |
| Curtis and Taylor (2009) |  |  |  |  |  |  |  |  |  |  |  |  |  |  |  |
| Dalton and Radtke (2013) |  |  |  |  |  |  |  |  |  |  |  |  |  |  |  |
| Elias (2008) |  |  |  |  |  |  |  |  |  |  |  |  |  |  |  |
| Ellis and Arieli (1999) |  |  |  |  |  |  |  |  |  |  |  |  |  |  |  |
| Erickson et al. (2017) |  |  |  |  |  |  |  |  |  |  |  |  |  |  |  |
| Erkmen et al. (2014) |  |  |  |  |  |  |  |  |  |  |  |  |  |  |  |
| Erturk and Donmez (2016) |  |  |  |  |  |  |  |  |  |  |  |  |  |  |  |
| Fieger and Rice (2018) |  |  |  |  |  |  |  |  |  |  |  |  |  |  |  |
| Fleming et al. (2018) |  |  |  |  |  |  |  |  |  |  |  |  |  |  |  |
| Francalanza and Buttigieg (2016) |  |  |  |  |  |  |  |  |  |  |  |  |  |  |  |
| Fredin et al. (2019) |  |  |  |  |  |  |  |  |  |  |  |  |  |  |  |
| Fredin (2011) |  |  |  |  |  |  |  |  |  |  |  |  |  |  |  |
| Gao et al. (2015) |  |  |  |  |  |  |  |  |  |  |  |  |  |  |  |
| Grube et al. (2010) |  |  |  |  |  |  |  |  |  |  |  |  |  |  |  |
| Gökçe (2013a) |  |  |  |  |  |  |  |  |  |  |  |  |  |  |  |
| Gökçe (2013b) |  |  |  |  |  |  |  |  |  |  |  |  |  |  |  |
| Gökçe (2013c) |  |  |  |  |  |  |  |  |  |  |  |  |  |  |  |
| Gökçe (2013d) |  |  |  |  |  |  |  |  |  |  |  |  |  |  |  |
| Gökçe (2013e) |  |  |  |  |  |  |  |  |  |  |  |  |  |  |  |
| Gökçe (2015) |  |  |  |  |  |  |  |  |  |  |  |  |  |  |  |
| Gundlach et al. (2008) |  |  |  |  |  |  |  |  |  |  |  |  |  |  |  |
| Guthrie and Taylor (2017) |  |  |  |  |  |  |  |  |  |  |  |  |  |  |  |
| Hechanova and Manaois (2020) |  |  |  |  |  |  |  |  |  |  |  |  |  |  |  |
| Henningsen et al. (2013) |  |  |  |  |  |  |  |  |  |  |  |  |  |  |  |
| Hwang et al. (2008) |  |  |  |  |  |  |  |  |  |  |  |  |  |  |  |
| Hwang et al. (2014) |  |  |  |  |  |  |  |  |  |  |  |  |  |  |  |
| Ion et al. (2015) |  |  |  |  |  |  |  |  |  |  |  |  |  |  |  |
| Ion et al. (2016) |  |  |  |  |  |  |  |  |  |  |  |  |  |  |  |
| Izraeli and Jaffe (1998) |  |  |  |  |  |  |  |  |  |  |  |  |  |  |  |
| Jackson et al. (2010) |  |  |  |  |  |  |  |  |  |  |  |  |  |  |  |
| Jenkel and Haen (2012) |  |  |  |  |  |  |  |  |  |  |  |  |  |  |  |
| Jones et al. (2014) |  |  |  |  |  |  |  |  |  |  |  |  |  |  |  |
| Kamarunzaman et al. (2014) |  |  |  |  |  |  |  |  |  |  |  |  |  |  |  |
| Kaplan et al. (2009) |  |  |  |  |  |  |  |  |  |  |  |  |  |  |  |
| Kaplan (1995) |  |  |  |  |  |  |  |  |  |  |  |  |  |  |  |
| Kaplan et al. (2009) |  |  |  |  |  |  |  |  |  |  |  |  |  |  |  |
| Kaplan et al. (2010) |  |  |  |  |  |  |  |  |  |  |  |  |  |  |  |
| Kaplan et al. (2011) |  |  |  |  |  |  |  |  |  |  |  |  |  |  |  |
| Kaplan and Schultz (2007) |  |  |  |  |  |  |  |  |  |  |  |  |  |  |  |
| Kaplan and Whitecotton (2001) |  |  |  |  |  |  |  |  |  |  |  |  |  |  |  |
| Kaptein (2011) |  |  |  |  |  |  |  |  |  |  |  |  |  |  |  |
| Keenan (1995) |  |  |  |  |  |  |  |  |  |  |  |  |  |  |  |
| Keenan (2000) |  |  |  |  |  |  |  |  |  |  |  |  |  |  |  |
| Keenan (2002a) |  |  |  |  |  |  |  |  |  |  |  |  |  |  |  |
| Keenan (2002b) |  |  |  |  |  |  |  |  |  |  |  |  |  |  |  |
| Keenan (2007) |  |  |  |  |  |  |  |  |  |  |  |  |  |  |  |
| Keil et al. (2007) |  |  |  |  |  |  |  |  |  |  |  |  |  |  |  |
| Keil et al. (2018) |  |  |  |  |  |  |  |  |  |  |  |  |  |  |  |
| Keil et al. (2004) |  |  |  |  |  |  |  |  |  |  |  |  |  |  |  |
| Keil et al. (2010) |  |  |  |  |  |  |  |  |  |  |  |  |  |  |  |
| Kennett et al. (2011) |  |  |  |  |  |  |  |  |  |  |  |  |  |  |  |
| King (1997) |  |  |  |  |  |  |  |  |  |  |  |  |  |  |  |
| King and Hermodson (2000) |  |  |  |  |  |  |  |  |  |  |  |  |  |  |  |
| King and Scudder (2013) |  |  |  |  |  |  |  |  |  |  |  |  |  |  |  |
| Latan et al. (2019a) |  |  |  |  |  |  |  |  |  |  |  |  |  |  |  |
| Latan et al. (2019b) |  |  |  |  |  |  |  |  |  |  |  |  |  |  |  |
| Latan et al. (2019c) |  |  |  |  |  |  |  |  |  |  |  |  |  |  |  |
| Latan et al. (2018) |  |  |  |  |  |  |  |  |  |  |  |  |  |  |  |
| Lavena (2016) |  |  |  |  |  |  |  |  |  |  |  |  |  |  |  |
| Lee et al. (2004) |  |  |  |  |  |  |  |  |  |  |  |  |  |  |  |
| Li and Ma (2016) |  |  |  |  |  |  |  |  |  |  |  |  |  |  |  |
| Lim and See (2001) |  |  |  |  |  |  |  |  |  |  |  |  |  |  |  |
| Liu and Ren (2017) |  |  |  |  |  |  |  |  |  |  |  |  |  |  |  |
| Liu et al. (2015) |  |  |  |  |  |  |  |  |  |  |  |  |  |  |  |
| Liu et al. (2016) |  |  |  |  |  |  |  |  |  |  |  |  |  |  |  |
| Liyanarachchi and Newdick (2009) |  |  |  |  |  |  |  |  |  |  |  |  |  |  |  |
| Liyanarachchi and Adler (2011) |  |  |  |  |  |  |  |  |  |  |  |  |  |  |  |
| Lowe et al. (2015) |  |  |  |  |  |  |  |  |  |  |  |  |  |  |  |
| Lyndon et al. (2012) |  |  |  |  |  |  |  |  |  |  |  |  |  |  |  |
| MacGregor and Stuebs (2014) |  |  |  |  |  |  |  |  |  |  |  |  |  |  |  |
| MacNab et al. (2007) |  |  |  |  |  |  |  |  |  |  |  |  |  |  |  |
| MacNab and Worthley (2008) |  |  |  |  |  |  |  |  |  |  |  |  |  |  |  |
| Mbago et al. (2018) |  |  |  |  |  |  |  |  |  |  |  |  |  |  |  |
| McCutcheon (2006) |  |  |  |  |  |  |  |  |  |  |  |  |  |  |  |
| McManus et al. (2012) |  |  |  |  |  |  |  |  |  |  |  |  |  |  |  |
| Miceli et al. (1991) |  |  |  |  |  |  |  |  |  |  |  |  |  |  |  |
| Miceli et al. (2012) |  |  |  |  |  |  |  |  |  |  |  |  |  |  |  |
| Miceli et al. (1991) |  |  |  |  |  |  |  |  |  |  |  |  |  |  |  |
| Miceli and Near (1984) |  |  |  |  |  |  |  |  |  |  |  |  |  |  |  |
| Miceli and Near (1985) |  |  |  |  |  |  |  |  |  |  |  |  |  |  |  |
| Miceli and Near (1988) |  |  |  |  |  |  |  |  |  |  |  |  |  |  |  |
| Milliken et al. (2003) |  |  |  |  |  |  |  |  |  |  |  |  |  |  |  |
| Moore and McAuliffe (2010) |  |  |  |  |  |  |  |  |  |  |  |  |  |  |  |
| Moore and McAuliffe (2012) |  |  |  |  |  |  |  |  |  |  |  |  |  |  |  |
| Nawawi and Salin (2018) |  |  |  |  |  |  |  |  |  |  |  |  |  |  |  |
| Nayir et al. (2018) |  |  |  |  |  |  |  |  |  |  |  |  |  |  |  |
| Near et al. (1993) |  |  |  |  |  |  |  |  |  |  |  |  |  |  |  |
| Near et al. (2004) |  |  |  |  |  |  |  |  |  |  |  |  |  |  |  |
| Nurhidayat and Kusumasari (2019) |  |  |  |  |  |  |  |  |  |  |  |  |  |  |  |
| Oelrich (2019) |  |  |  |  |  |  |  |  |  |  |  |  |  |  |  |
| Olesen et al. (2019) |  |  |  |  |  |  |  |  |  |  |  |  |  |  |  |
| Omotoye (2017) |  |  |  |  |  |  |  |  |  |  |  |  |  |  |  |
| Park et al. (2008) |  |  |  |  |  |  |  |  |  |  |  |  |  |  |  |
| Park and Keil (2009) |  |  |  |  |  |  |  |  |  |  |  |  |  |  |  |
| Park et al. (2009) |  |  |  |  |  |  |  |  |  |  |  |  |  |  |  |
| Park and Blenkinsopp (2009) |  |  |  |  |  |  |  |  |  |  |  |  |  |  |  |
| Park et al. (2005) |  |  |  |  |  |  |  |  |  |  |  |  |  |  |  |
| Perry et al. (1997) |  |  |  |  |  |  |  |  |  |  |  |  |  |  |  |
| Pillay et al. (2012) |  |  |  |  |  |  |  |  |  |  |  |  |  |  |  |
| Pillay et al. (2018) |  |  |  |  |  |  |  |  |  |  |  |  |  |  |  |
| Pillay et al. (2017) |  |  |  |  |  |  |  |  |  |  |  |  |  |  |  |
| Pope and Lee (2013) |  |  |  |  |  |  |  |  |  |  |  |  |  |  |  |
| Previtali and Cerchiello (2018) |  |  |  |  |  |  |  |  |  |  |  |  |  |  |  |
| Proost et al. (2013) |  |  |  |  |  |  |  |  |  |  |  |  |  |  |  |
| Radulovic and Uys (2019) |  |  |  |  |  |  |  |  |  |  |  |  |  |  |  |
| Reckers-Sauciuc and Lowe (2010) |  |  |  |  |  |  |  |  |  |  |  |  |  |  |  |
| Rennie and Crosby (2002) |  |  |  |  |  |  |  |  |  |  |  |  |  |  |  |
| Richardson et al. (2012) |  |  |  |  |  |  |  |  |  |  |  |  |  |  |  |
| Richardson et al. (2008) |  |  |  |  |  |  |  |  |  |  |  |  |  |  |  |
| Robertson et al. (2011) |  |  |  |  |  |  |  |  |  |  |  |  |  |  |  |
| Robinson et al. (2012) |  |  |  |  |  |  |  |  |  |  |  |  |  |  |  |
| Rose et al. (2018) |  |  |  |  |  |  |  |  |  |  |  |  |  |  |  |
| Rothwell and Baldwin (2006) |  |  |  |  |  |  |  |  |  |  |  |  |  |  |  |
| Rothwell and Baldwin (2007a) |  |  |  |  |  |  |  |  |  |  |  |  |  |  |  |
| Rothwell and Baldwin (2007b) |  |  |  |  |  |  |  |  |  |  |  |  |  |  |  |
| Rustiarini and Sunarsih (2017) |  |  |  |  |  |  |  |  |  |  |  |  |  |  |  |
| Satalkar and Shaw (2018) |  |  |  |  |  |  |  |  |  |  |  |  |  |  |  |
| Scheetz and Fogarty (2019) |  |  |  |  |  |  |  |  |  |  |  |  |  |  |  |
| Scheetz and Wilson (2019) |  |  |  |  |  |  |  |  |  |  |  |  |  |  |  |
| Schultz et al. (1993) |  |  |  |  |  |  |  |  |  |  |  |  |  |  |  |
| Seifert et al. (2014) |  |  |  |  |  |  |  |  |  |  |  |  |  |  |  |
| Seifert et al. (2010) |  |  |  |  |  |  |  |  |  |  |  |  |  |  |  |
| Shawver (2008) |  |  |  |  |  |  |  |  |  |  |  |  |  |  |  |
| Shawver (2011a) |  |  |  |  |  |  |  |  |  |  |  |  |  |  |  |
| Shawver (2011b) |  |  |  |  |  |  |  |  |  |  |  |  |  |  |  |
| Sims and Keenan (1999) |  |  |  |  |  |  |  |  |  |  |  |  |  |  |  |
| Sims and Keenan (1998) |  |  |  |  |  |  |  |  |  |  |  |  |  |  |  |
| Singer et al. (1998) |  |  |  |  |  |  |  |  |  |  |  |  |  |  |  |
| Smith et al. (2001) |  |  |  |  |  |  |  |  |  |  |  |  |  |  |  |
| Somers and Casal (2011) |  |  |  |  |  |  |  |  |  |  |  |  |  |  |  |
| Somers and Casal (1994) |  |  |  |  |  |  |  |  |  |  |  |  |  |  |  |
| Soni et al. (2015) |  |  |  |  |  |  |  |  |  |  |  |  |  |  |  |
| Stansbury and Victor (2009) |  |  |  |  |  |  |  |  |  |  |  |  |  |  |  |
| Stikeleather (2016) |  |  |  |  |  |  |  |  |  |  |  |  |  |  |  |
| Stöber et al. (2019) |  |  |  |  |  |  |  |  |  |  |  |  |  |  |  |
| Su et al. (2010) |  |  |  |  |  |  |  |  |  |  |  |  |  |  |  |
| Surya et al. (2017) |  |  |  |  |  |  |  |  |  |  |  |  |  |  |  |
| Tan et al. (2003) |  |  |  |  |  |  |  |  |  |  |  |  |  |  |  |
| Tavakoli et al. (2003) |  |  |  |  |  |  |  |  |  |  |  |  |  |  |  |
| Taylor and Curtis (2010) |  |  |  |  |  |  |  |  |  |  |  |  |  |  |  |
| Taylor and Curtis (2013) |  |  |  |  |  |  |  |  |  |  |  |  |  |  |  |
| Taylor and Curtis (2018) |  |  |  |  |  |  |  |  |  |  |  |  |  |  |  |
| Taylor (2018) |  |  |  |  |  |  |  |  |  |  |  |  |  |  |  |
| Taylor (2019) |  |  |  |  |  |  |  |  |  |  |  |  |  |  |  |
| Teichmann (2019) |  |  |  |  |  |  |  |  |  |  |  |  |  |  |  |
| Thoroughgood et al. (2011) |  |  |  |  |  |  |  |  |  |  |  |  |  |  |  |
| Trevino and Victor (1992) |  |  |  |  |  |  |  |  |  |  |  |  |  |  |  |
| Trongmateerut and Sweeney (2013) |  |  |  |  |  |  |  |  |  |  |  |  |  |  |  |
| Tumuramye et al. (2018) |  |  |  |  |  |  |  |  |  |  |  |  |  |  |  |
| Ugaddan and Park (2019) |  |  |  |  |  |  |  |  |  |  |  |  |  |  |  |
| Victor et al. (1993) |  |  |  |  |  |  |  |  |  |  |  |  |  |  |  |
| Vincent et al. (1999) |  |  |  |  |  |  |  |  |  |  |  |  |  |  |  |
| Wainberg and Perreault (2016) |  |  |  |  |  |  |  |  |  |  |  |  |  |  |  |
| Wen and Chen (2016) |  |  |  |  |  |  |  |  |  |  |  |  |  |  |  |
| Whitaker et al. (2014) |  |  |  |  |  |  |  |  |  |  |  |  |  |  |  |
| Wilson et al. (2018) |  |  |  |  |  |  |  |  |  |  |  |  |  |  |  |
| Xu and Ziegenfuss (2008) |  |  |  |  |  |  |  |  |  |  |  |  |  |  |  |
| Yu et al. (2019) |  |  |  |  |  |  |  |  |  |  |  |  |  |  |  |
| Zarefar and Zarefar (2017) |  |  |  |  |  |  |  |  |  |  |  |  |  |  |  |
| Zhang et al. (2016) |  |  |  |  |  |  |  |  |  |  |  |  |  |  |  |
| Zhang et al. (2013) |  |  |  |  |  |  |  |  |  |  |  |  |  |  |  |
| Zhang et al. (2009a) |  |  |  |  |  |  |  |  |  |  |  |  |  |  |  |
| Zhang et al. (2009b) |  |  |  |  |  |  |  |  |  |  |  |  |  |  |  |
| Zhang (2008) |  |  |  |  |  |  |  |  |  |  |  |  |  |  |  |
| Zheng et al. (2019) |  |  |  |  |  |  |  |  |  |  |  |  |  |  |  |
| Zhou et al. (2018) |  |  |  |  |  |  |  |  |  |  |  |  |  |  |  |
| Zhuang et al. (2005) |  |  |  |  |  |  |  |  |  |  |  |  |  |  |  |
| Zipparo (1999) |  |  |  |  |  |  |  |  |  |  |  |  |  |  |  |

Risk of Bias Key: α = Low,  = Unclear,  = High

Table Note: The risks of bias of studies included were assessed using the criteria below. Studies were assessed as having a) no or low risk of bias, or b) potential risk of bias. Criterion for all studies involved: Sampling (1. Participants are randomly selected, 2. Sample sizes are adequate, 3. Participants are representative of various demographic groups, 4. If some participants were excluded from the analyses, the exclusion is justified, 5. When group comparisons were made, participants were matched on other meaningful demographics, and 15. Other risks of bias), and measures (i.e., 6. Validated measures are used, or the authors have provided sufficient supportive information of the psychometric properties of the measures they devised and 7. Measures used were clearly defined and were appropriate). The criterion for studies that adopted a longitudinal or prospective design included: 8. Authors examined whether dropout is random. 9. Missing data were treated appropriately. Finally, the following criterion was used for experimental designs: 10. Allocation sequence generated to produce comparable groups. 11. Allocation was concealed, 12. Whether blinding was done and the effectiveness of it, 13. Outcome data for all outcomes were reported. Incomplete outcomes due to attrition and exclusions were addressed, and 14. No selective outcome reporting

Supplementary Table 2. Study Characteristics

| **Authors and Year** | **Participant information** | **Instrumentation** | **Design** | **Risk of bias assessment** | **Main findings** |
| --- | --- | --- | --- | --- | --- |
| **Ahern and McDonald (2002)** | 95 registered general and mental health nurses in Western Australia (73% female). | A questionnaire that consisted of belief statements to whistleblowing, patient advocacy, and traditional roles of nursing. | Quantitative observational | High | ◦ Whistleblowers agreed more strongly with the advocacy statements while non-whistleblowers agreed more strongly with the traditional statements. |
| **Ahmad et al.(2013)** | 180 internal auditors from Malaysia (98 males and 82 females). | A questionnaire that consisted of scenarios and questions assessing internal whistleblowing intentions, seriousness of wrongdoing, and status of the wrongdoer. | Quantitative observational | High | ◦ Seriousness of wrongdoing was the strongest predictor of internal whistleblowing intentions.  ◦ Whistleblowing intentions was greater for less powerful wrongdoers. |
| **Ahmad et al. (2014)** | 180 members of an internal auditorsorganization in Malaysia. | A questionnaire presented scenarios and questions that assessed ethical climate, participants contextual details (e.g., organization size), and whistleblowing intentions. | Quantitative observational | Unclear | ◦ Perception of principle climate was a significant predictor of internal whistleblowing intentions among internal auditors. ◦ Seriousness of wrongdoing is the most consistent predictor for internal whistleblowing intentions. |
| **Alinaghian et al. (2018)** | 247 staff members of an Iranian hospital (149 nurses, 101 administrative staff, and 24 interns and specialists). | A questionnaire that measured factors that influence whistleblowing (i.e., organizational cultural, individual, organizational structure, and materiality of wrongdoing). | Quantitative observational | High | ◦ Organizational culture, organization structure, and the materiality of wrongdoing had a significant positive relationship with whistleblowing. |
| **Alleyne (2016)** | 236 non-public accountants from corporate organizations in Barbados (104 males and 132 females). | A questionnaire comprised of scales that assess participants' whistleblowing intentions, organizational commitment, and corporate ethical values. | Quantitative observational | Low | ◦ Factors that encourage whistleblowing are ethical management, confidence in the reporting system, ethical culture in the organization, trust in management, and commitment to the organization.  ◦ Factors that discourage whistleblowing include distrust in the organization's systems, unethical senior management, poor work climate, dismissal of Whistleblower, possible job loss, and fear of reprisal from management. |
| **Alleyne et al. (2017)** | 282 accounting employees in Barbados (161 females and 121 males). | A questionnaire that assessed whistleblowing perceptions, awareness of organizational wrongdoings, whistleblowing intentions, perceived seriousness, and perceived personal cost and perceived personal responsibility. | Mixed Methods observational | High | ◦ Personal responsibility was a predictor of internal whistleblowing. ◦ Personal cost influenced both internal and external whistleblowing intentions. ◦ Factors that influenced whistleblowing were perceived benefits of whistleblowing, actual whistleblowing experiences, personal costs, perceived lack of anonymity, and cultural norms. |
| **Alleyne et al. (2019)** | 226 accountants in Barbados (124 females and 102 males). | A questionnaire consisting of scenarios with questions related to their attitude toward whistleblowing, social desirability, group cohesion, independence commitment, moral approbation, reporting intentions, perceived responsibility and cost, and perceived behavioral control. | Quantitative observational | Low | ◦ Attitude towards whistleblowing, perceived behavioral control, independence commitment, personal responsibility to report, and personal cost of reporting were all significantly related to internal whistleblowing intentions.  ◦ External whistleblowing intentions were significantly related to perceived behavioral control and personal responsibility for reporting.  ◦ Group cohesion was found to have a moderating role. |
| **Alleyne et al. (2018)** | 226 public accountants in Barbados (124 females and 102 males). | A questionnaire that presented a scenario and questions related to whistleblowing intentions, attitudes, perceived behavior control, perceived personal responsibility and cost of reporting, and perceived organizational support. | Quantitative observational | Low | ◦ Internal whistleblowing intentions were positively associated with attitude, perceived behavioral control, independence commitment, personal responsibility for reporting.  ◦ Internal whistleblowing intentions were negatively associated with personal cost of reporting.  ◦ External whistleblowing intentions were positively associated with perceived behavioral control.  ◦ Organizational support influenced the preferred channel of reporting wrongdoing (internal vs external). |
| **Alleyne et al. (2013)** | 236 non-public accountants from corporate organizations in Barbados (104 males and 132 females). | A questionnaire comprised of scales measuring level of awareness of whistleblowing, perception of whistleblowing, whistleblowing intentions, organizational commitment, and corporate ethical values. | Quantitative observational | Low | ◦ Factors that encourage whistleblowing were job satisfaction, severity of the incident, anonymity, personal benefits, and the need to correct wrongdoing that may harm the organization.  ◦ Factors that discourage participants from whistleblowing include the close relationship to the wrongdoer, publicity that may negatively impact the organization, and fear of retaliation and victimization from both management and other employees. |
| **Andon et al. (2018)** | 80 U.S.-based professional accountants (52 females and 28 males). | Participants were presented with a scenario that varied according to the availability of financial incentives for whistleblowing (yes or no). The participants were asked to indicate their whistleblowing intentions, and seriousness of the wrongdoing. | Quantitative experimental | High | ◦ Providing a financial incentive increased external whistleblowing intentions.  ◦ Perceptions of the wrongdoing seriousness are positively associated with external whistleblowing intentions. |
| **Arifah et al. (2017)** | 346 employees of an enforcement agency in Malaysia (272 males and 74 females). | A questionnaire that assessed organizational trust and whistleblowing intentions. | Quantitative observational | High | ◦ Organizational trust had a positive relationship to whistleblowing intentions (internal and external). |
| **Atkinson et al. (2012)** | 47 college students. | A questionnaire that consisted of scenarios with questions assessing whistleblowing intentions. | Quantitative experimental | Unclear | ◦ The availability of an anonymous reporting system (e.g., hotline or website) increased whistleblowing intentions. |
| **Attree (2007)** | 142 practicing nurses from three Acute NHS Trusts in England (132 females and 10 males). | A semi-structured interview exploring Registered Nurses’ perceptions of standards of nursing practice, discover whether nurses have concerns about practice standards, and, if so, identify how they deal with them. | Qualitative observational | High | ◦ Fear of negative consequences (e.g., retaliation, repercussions) and a lack of confidence in organizational reporting systems discouraged whistleblowing. |
| **Aydan and Kaya (2018)** | 369 nurses (n=167) and secretaries (n=202) from a Turkish university hospital (336 females and 33 males). | A questionnaire consisting of scales assessing ethical climate, organizational trust, and whistleblowing intentions. | Quantitative observational | Low | ◦ Ethical climate positively influenced whistleblowing intentions. ◦ Ethical climate moderated the interaction between organizational trust and whistleblowing intentions. |
| **Ayers and Kaplan (2005)** | 74 postgraduate business students at a U.S.-based university (53% were male). | Participants were presented with one of two scenarios that varied on the committed wrongdoing. The participants were asked to indicate their whistleblowing intentions, the perceived seriousness, perceived personal cost, perceived personal responsibility, moral equity, relativism, and contractualism. | Quantitative experimental | High | ◦ The perceived seriousness of wrongdoing and personal costs were associated with whistleblowing intentions under both non-anonymous and anonymous reporting channel. ◦ Personal responsibility perceptions and moral equity judgments were associated with whistleblowing intentions through non-anonymous channel only. |
| **Barnett (1992)** | 240 executives from U.S.-based manufacturing, financial, and services industries. | A questionnaire that assessed characteristics of a participant's organization and whistleblowing. | Quantitative observational | Low | ◦ Organizational size, unionization, and type of industry may affect the level of external whistleblowing about sensitive legal or moral issues. |
| **Barnett et al. (1996)** | 267 business students (55% were male). | A questionnaire comprised of a scenario with questions assessing religiosity, ethical ideology, ethical judgment, whistleblowing intentions. | Quantitative observational | High | ◦ The relationship between ethical ideologies and whistleblowing intentions is mediated perceived ethicality of whistleblowing. |
| **Barnett et al. (1993)** | 295 human resource executives of private-sector organizations (74% were male). | A questionnaire that assessed the management's responsiveness to sensitive issues, the existence of internal disclosure policy/procedure, and the perceptions of the occurrence of internal disclosure and external whistleblowing. | Quantitative observational | Low | ◦ Internal disclosure policies/procedures (IDPP) are associated with a higher level of internal disclosures by employees.  ◦ Implementation of IDPP resulted in a significant increase in the level of internal disclosures after the implementation of IDPP's.  ◦ The more internal disclosures occurred among companies with formal IDPP than those without such policies. |
| **Bellefontaine (2009)** | 6 student nurses in the U.K. | A semi-structured interview that focused on the participant's ability to report potentially unsafe practice witnessed in clinical placements. | Qualitative observational | High | ◦ Factors that influenced reporting were perceptions of support from mentors and peers, confidence and knowledge base, and fear of consequences. |
| **Berger et al. (2017)** | 166 accounting master's students at two North American universities (55% were female). | Participants were presented with one of three scenarios that varied on availability on monetary reward for reporting fraud (eligible, ineligible, or no incentive). The participants were asked to indicate the likelihood to blow the whistle. | Quantitative experimental | High | ◦ Individuals were less likely to blow the whistle immediately if they do not qualify for an organization's fraud reporting incentives versus when they do or when financial rewards are not mentioned at all. ◦ Individuals were more likely of delayed reporting of wrongdoing when the Whistleblower does not qualify for an incentive versus when they do or when financial rewards are not mentioned at all. |
| **Bhal and Dadhich (2011)** | 248 postgraduate students of an engineering institute in India.  Study 1 comprised of 81 students (16 females and 81 males), 80 students participated in study 2 (23 females and 57 males), and study 3 consisted of 87 students (23 females and 64 males). | Participants were presented with scenarios and questions measuring their willingness to report. For study 1, the scenarios varied according to the leader's behavior (ethical or unethical) and the quality of leader-member exchange (high or low). In study 2, the scenarios varied according to the leader's behavior (ethical or unethical) and the magnitude of the consequences (high or low). For study 3, the scenarios varied according the quality of the leader-member exchange (high or low) and the magnitude of the consequences (high or low). | Quantitative experimental | High | ◦ Both ethical leadership and leader-member exchange predicted whistleblowing intentions.  ◦ The relationships between whistleblowing intentions and both ethical leadership and leader-member exchange were moderated by the moral intensity. |
| **Binikos (2008)** | 129 employees of an ICT company in South Africa (48.1% were female) | A questionnaire that assessed organizational trust, whistleblowing, and knowledge and behaviors of reporting at their employer. | Quantitative observational | High | ◦ Organizational trust was positively related to internal whistleblowing intentions. |
| **Bjørkelo et al. (2010)** | 3042 municipal (n=503; 70% were female) and postal service (n=2539; 52% were female) employees in Norway. | Study 1: A questionnaire that contained NEO-FFI and questions about whistleblowing history. Study 2: A questionnaire that contained the Inventory of Interpersonal Problems - Circumplex and questions about whistleblowing history. | Quantitative observational | Low | ◦ High extraversion, low agreeableness, and high domineering in interpersonal interaction predicted actual whistleblowing behavior. |
| **Black (2011)** | 564 registered nurses in the United States (513 females and 32 males) | A questionnaire that assessed work setting, reporting unsafe patient care practices or conditions, experiences with prior reporting activities, and attitudes toward reporting concerns about patient safety. | Quantitative observational | Low | ◦ The most common reasons for not reporting wrongdoing were fears of workplace retaliation and the belief that nothing would come of the made reports. |
| **Bocchiaro et al. (2012)** | 149 undergraduate students at a Dutch university (96 females and 53 males). | Participants completed a questionnaire that included the HEXACO-PI-R and Social Value Orientation scale along with engaging in a task where they had to deal with an unethical request by the experimenter with options of (dis)obeying or ‘‘blowing the whistle’. | Quantitative experimental | Low | ◦ Individuals with higher religious faith were more likely to blow the whistle than obedient and disobedient participants. |
| **Boo et al. (2016)** | 90 audit seniors and managers (57% were female). | Participants were presented with one of six scenarios that varied according to the incentive scheme (reward or penalty or none) and relationship level wrongdoer (close or not close). The participants were asked to indicate the likelihood of whistleblowing. | Quantitative experimental | High | ◦ A reward-based incentive scheme was less likely to increase whistleblowing intentions when there is a close working relationship between the Whistleblower and the wrongdoer. ◦ Both reward- and penalty-based incentive schemes increased whistleblowing intentions when a close working relationship between the Whistleblower and wrongdoer is absent. |
| **Brabeck (1984)** | 32 undergraduate students at U.S.-based university (26 females and 6 males). | Participants completed a questionnaire that contained the Defining Issues Test and a task involving them being tested on an article that has an error in it. | Quantitative experimental | High | ◦ Whistleblowers demonstrated a higher level of moral development than non-whistleblowers. |
| **Brennan and Kelly (2007)** | 100 accounting students at an institute in Ireland (59 females and 41 males). | A questionnaire that consisted of four scenarios with questions measuring the participant's organizations structures, firm size, seriousness of wrongdoing, whistleblowing behaviors, and the influence of legislation of whistleblowing decision. | Quantitative observational | High | ◦ Adequate formal reporting structures promoted greater confidence and increased the likelihood of reporting wrongdoings internally ◦ The willingness to report wrongdoing externally decreased with age. |
| **Brink et al. (2017)** | 82 M.B.A. students from a university in the U.S. (56 males and 30 females). | Participants were presented with one of two scenarios that varied according to the type of wrongdoing (insider trading or fraudulent financial reporting). The participants were asked to indicate their monetary attitudes, whistleblowing likelihood, perceived responsibility to report wrongdoing, and perceived seriousness of wrongdoing. | Quantitative experimental | Unclear | ◦ Perceived responsibility to report wrongdoing increased intentions to blow the whistle internally.  ◦ Perceived responsibility to report wrongdoing mediated the relationship between wrongdoing type and internal whistleblowing. ◦ Whistleblowing intentions were influenced by attitudes toward money. ◦ Perceived seriousness of the wrongdoing increased intentions to blow the whistle externally. |
| **Brink et al. (2013)** | 72 M.B.A. students from two major universities in the U.S. (34.7% were female). | Participants were presented with a scenario that varied according to the existence of internal whistleblowing incentives (present or absent) and strength of evidence (strong or weak). The participants were asked to indicate their whistleblowing intentions. | Quantitative experimental | Unclear | ◦ The interaction between evidence strength and internal incentive provided by their employer influenced whistleblowing behavior.  ◦ For weak evidence, an internal incentive decreased the likelihood of external reporting.  ◦ For strong evidence, the presence of an internal incentive increased the likelihood of external reporting. |
| **Brooks and Perot (1991)** | 490 faculty (n = 214) and postgraduate students (n = 276) at a university in the U.S. (100% were female). | A questionnaire that assessed sexual harassment experiences, perceived offensiveness of the experiences, and reporting behaviors. | Quantitative observational | Low | ◦ Perceived offensiveness of sexual harassment experience was positively related with the likelihood of reporting. ◦ The relationship between the likelihood of reporting and both feminist ideology and frequency of behavior was mediated by perceived offensiveness. |
| **Brown et al. (2016)** | 284 professional organizational accountants based in the U.S. (146 females and 138 males). | A questionnaire assessing whistleblowing intentions in hypothetical scenario, attitudes, subjective norms, and perceived behavioral control. | Quantitative observational | Low | ◦ Those individuals with higher perceived control, more influential referent groups, and a more positive attitude toward whistleblowing were more likely to report fraudulent activity. ◦ Females were more likely to report fraudulent accounting activity than males. ◦ Senior-level accountants were more likely to report fraudulent activity than accountants at middle- or lower-management levels. |
| **Caillier (2013)** | 964 government employees | A questionnaire consisting of the Global Transformation Leadership and scale and the Multifactor Leadership Questionnaire, along with questions pertaining to affective commitment, job satisfaction, and power-dependency. | Quantitative observational | Low | ◦ Immediate supervisors that practice transformational leadership had a positive influence on employees' comfort level with whistleblowing. ◦ Employees with high levels of affective commitment were more comfortable blowing the whistle than those with lower levels. ◦ Employees were more comfortable blowing the whistle when they were satisfied with their jobs. |
| **Caillier (2017a)** | 42020 U.S. federal employees. | A questionnaire that assessed whistleblowing education, organizational protection, and wrongdoing action severity. | Quantitative observational | Low | ◦ Whistleblowing education was positively associated with internal whistleblowing  ◦ Whistleblowing was negatively associated with retaliation. |
| **Caillier (2017b)** | 42020 U.S. federal government employees | The Merit Principles Survey (MPS) 2010 questionnaire | Quantitative observational | Low | ◦ Whistleblowers had higher levels of public service motivation than non-whistleblowers. ◦ Seriousness of wrongdoing had a positive effect on whistleblowing. ◦ Seriousness of wrongdoing moderated the relationship between public service motivation and internal whistleblowing only. |
| **Casal and Bogui (2008)** | 330 members of a professional accountant organization. | A questionnaire that assessed intentions to leave, stay and report, and leave and report in response to organizational wrongdoing, seriousness of wrongdoing, expected effectiveness of whistleblowing, and expected retaliation to whistleblowing, and role responsibility. | Quantitative observational | Unclear | ◦ Whistleblowing intentions increased with expected effectiveness of whistleblowing and role responsibility for reporting, regardless of the intentions to stay or leave the role. ◦ Whistleblowing intentions decreased with expected retaliation for whistleblowing when there is an intention to stay in the role. |
| **Cassematis and Wortley (2013)** | 3232 employees from the Australian public sector organizations (1692 females and 1541 males). | The Workplace Experiences and Relationships questionnaire, which assessed whistleblowing propensity, job satisfaction, organizational citizenship behavior, fear of reprisal, perceived wrongdoing seriousness, and personal victimization. | Quantitative observational | Low | ◦ Whistleblowers had a more positive attitudes towards the act.  ◦ Whistleblowers have a stronger belief that the act is valued by the organization. ◦ Fear of reprisals was a factor that most concerning for non-Whistleblower. ◦ Being personally affected by the wrongdoing was the most influential predictor of whistleblowing. |
| **Chaudhary et al. (2019)** | 298 teachers from higher education institutions in India. | A questionnaire that assessed perceived cost of reporting, status of wrongdoer, seriousness of wrongdoing, whistleblowing intentions, the availability of communication channel, and perceived organizational support. | Quantitative observational | Low | ◦ Availability of a proper communication channel in the organization increased the likelihood of reporting the wrongdoing internally. ◦ The high status of the wrongdoer and high costs of reporting discouraged internal whistleblowing and encouraged reporting externally. ◦ Factor discouraging internal whistleblowing is the potential negative repercussions.  ◦ There is a greater intention to blow the whistle internally (vs externally for serious wrongdoings. |
| **Chen and Lai (2014)** | 533 individuals in Taiwan (51.4% were female) | A questionnaire that consisted of a scenario with questions assessing moral intensity, organizational commitment, and whistleblowing intentions and behaviors. | Quantitative observational | Low | ◦ The moral intensity dimension of potential harm was positively correlated with whistleblowing intention. ◦ Potential harm and social pressure affect Whistleblower's choice of communication channel (i.e., internal or external) differently. ◦ Organizational commitment has a moderated mediation effect on moral intensity, whistleblowing intention, and whistleblowing behavior. |
| **Chen et al. (2017)** | 147 undergraduate students. | Following the random assignment into one of four groups, the participants completed a series of tasks. At the conclusion of each task, the participants were asked to report their score and whether a group member had overstated their score to the experimenter. | Quantitative experimental | Unclear | ◦ When an organization presents strong descriptive norms supporting whistleblowing, penalties lead to a greater increase in internal whistleblowing. |
| **Cheng et al. (2019)** | 230 employees of retail in China (140 females and 90 males). | A questionnaire that assessed ethical leadership, perceived organizational politics, moral courage, and internal whistleblowing. | Quantitative observational | Low | ◦ Ethical leadership had positive relationship with internal whistleblowing. ◦ Perceived organizational politics was negatively associated with internal whistleblowing ◦ The relationship between ethical leadership and internal whistleblowing was mediated by perceived organizational politics.  ◦ Moral courage moderated the relationship between perceived organizational politics and internal whistleblowing. |
| **Chiasson et al. (1995)** | 244 certified public accountants (75% were male). | A questionnaire that assessed the internal and external whistleblowing intentions. | Quantitative observational | High | ◦ The probability of blow the whistle internally were highest for theft of organization funds, followed by accepting bribes and wrongdoings that could possibly cause injury to employees or the public. ◦ The probability of whistleblowing was lowest for the making on overly optimistic and false projections of future performance by management. |
| **Chiu (2002)** | 254 M.B.A. students in China. | A questionnaire consisting of a scenario with questions assessing ethical judgment, locus of control, and whistleblowing intentions. | Quantitative observational | Unclear | ◦ Locus of control moderated the relationship between ethical judgment and whistleblowing intention. |
| **Chiu (2003)** | 306 managers and professionals from China. | A questionnaire consisting of a scenario with questions assessing ethical judgment, locus of control, and whistleblowing intentions. | Quantitative observational | Low | ◦ Ethical judgment was positively related to whistleblowing intention. ◦ Locus of control was negatively related to whistleblowing intention. ◦ Locus of control moderated the relationship between ethical judgment and whistleblowing intention. |
| **Chiu and Erdener (2003)** | 362 managers and executives in China (161 females and 201 males). | A questionnaire consisting of a scenario and questions assessing ethical judgement, ethical ideology, locus of control, and whistleblowing intentions. | Quantitative observational | Low | Locus of control moderated the relationship between ethical judgement and whistleblowing intention. Ethical judgement was positively related to whistleblowing intention. |
| **Cho and Song (2015)** | 42020 U.S. federal government employees | The Merit Principles Survey (MPS) 2010 questionnaire | Quantitative observational | High | ◦ Whistleblowing intentions is decreased by perceived personal costs of the act.  ◦ The effect of perceived personal cost on whistleblowing intentions is alleviated by organizational support and organizational protection. ◦ Public service motivation and education increased whistleblowing intentions. |
| **Clements and Shawver (2011)** | 171 public accountants (109 males and 62 females) | A questionnaire that consisted of a scenario and questions assessing moral intensity, ethicality of situation, and whistleblowing likelihood. | Quantitative observational | High | ◦ Social consensus is a factor considered by accountants when deciding whether to blow the whistle internally. |
| **Clements and Shawver (2009)** | 36 accounting professionals in the U.S. | A questionnaire that consisted of scenarios and questions assessing ethics and whistleblowing intentions. | Quantitative observational | High | ◦ No relationship was found between ethics and whistleblowing intentions. |
| **Curtis (2006)** | 220 accounting students from a U.S.-based university (142 females and 78 males) | Immediately, after the completion of an examination, the participants completed a questionnaire consisting of a scenario with questions related to whistleblowing intentions and the participant's affect. | Quantitative observational | High | ◦ Negative mood was associated with lower intentions to report wrongdoings.  ◦ Seriousness and responsibility moderated the relationship between affect and whistleblowing intentions. ◦ Personal cost is negatively influenced the intentions to report wrongdoings. |
| **Curtis and Taylor (2009)** | 122 in-charge auditors. | A questionnaire that consisted of three scenarios that varied according to moral intensity and reporting formats. The participants were asked to indicate their likelihood of reporting, locus of control, ethical style, and trust in the organization responding to wrongdoing. | Quantitative experimental | Unclear | ◦ A disclosed identity format decreased the likelihood of whistleblowing, while there was no significant difference in likelihood between anonymous and protected identity formats.  ◦ Audit standards violations were more likely to be reported that than a professional code violation.  ◦ Trust that the firm would investigate and act on the reported wrongdoing increased the likelihood of whistleblowing ◦ Locus of control and ethical style were significant antecedents to whistleblowing intentions. |
| **Dalton and Radtke (2013)** | 116 MBA students at two U.S.-based universities (74 males and 42 females). | A questionnaire that consisted of a scenario varying according to the ethical environment (weak or strong) with questions assessing Machiavellianism, whistleblowing intentions, perceived seriousness, perceived costs/benefits and perceived responsibility. | Quantitative experimental | Unclear | ◦ Machiavellianism was negatively related to whistle-blowing intentions. ◦ Machiavellianism has an indirect effect on whistleblowing through perceived benefits and perceived responsibility.  ◦ A strong ethical environment increases whistleblowing intentions of those individuals who are higher in Machiavellianism. |
| **Elias (2008)** | 128 auditing students from two U.S.-based universities. | A questionnaire contained scenarios with questions assessing whistleblowing perceptions and intentions, professional commitment, and perceptions of financial reporting. | Quantitative observational | Unclear | ◦ Those individuals who exhibit greater professional committed to auditing students were more likely to blow the whistle.  ◦ A higher perception of financial reporting was associated with higher whistleblowing intentions. |
| **Ellis and Arieli (1999)** | 275 officers in the Israeli Defense Forces. | A questionnaire presenting scenarios with questions that measured general reporting impressions, whistleblowing attitudes, beliefs, and subjective norms. | Quantitative observational | High | ◦ Whistleblowing attitudes and subjective norms predicted whistleblowing intentions. ◦ The effect of subjective norms was stronger than the effect of attitudes. |
| **Erickson et al. (2017)** | 28 track and field university student-athletes from the UK (n = 14) and US (n = 14; 15 females and 13 males). | A semi-structured interview focused on general attitudes to PEDs and whistleblowing. | Qualitative observational | high | ◦ The willingness to report a wrongdoing was dependent on the level of relationship.  ◦ An individual was less likely to blow the whistle on someone they have a personal relationship with (e.g., friend) then those who are relationally distant. |
| **Erkmen et al. (2014)** | 116 professional accountants from Turkey (100 males and 16 females). | A questionnaire that contained scenarios and questions measuring whistleblowing intentions. | Quantitative observational | Low | ◦ Females were more likely to blow the whistle than males. ◦ Older accounting professionals will be more likely to blow the whistle. |
| **Erturk and Donmez (2016)** | 393 schoolteachers from Turkey (56% were female). | A questionnaire that consisted of scales assessing whistleblowing behaviors and school principal's leadership style. | Quantitative observational | High | ◦ Laissez-faire leadership style positively associated with anonymous whistleblowing behaviors. ◦ Transformational leadership has a positive relationship with internal whistleblowing. |
| **Fieger and Rice (2018)** | 2375 Australian Public Service employees who observed a potential corrupt act (46.3% were female). | 2014 Australian Public Service Census | Quantitative observational | High | ◦ Females more likely to report wrongdoings than males.  ◦ Individuals in smaller organizational units are less likely to blow the whistle than those larger ones. ◦ The longer someone is with an organization, the less likely they will blow the whistle. |
| **Fleming et al. (2018)** | 288 surgical trainees. | A questionnaire that explored the experiences of surgical trainees in raising concerns over patient safety and their attitudes towards whistleblowing in general. | Quantitative observational | High | ◦ Factors that discourage whistleblowing were the fear of personal vilification or reprisal, fear of impact on career, and a lack of confidence in the process. ◦ More senior trainees were significantly more likely to blow the whistle than their more junior peers. |
| **Francalanza and Buttigieg (2016)** | 330 certified public accountants from Malta (36.7% were female). | A two questionnaires that assessed the personality traits, general whistleblowing intentions, and the perception of situational factors. | Quantitative observational | Low | ◦ Whistleblowers showed significantly higher scores on extraversion, conscientiousness, and openness to experiences.  ◦ Non-whistleblowers showed significantly higher score on neuroticism. ◦ Situational factors that discouraged whistleblowing were retaliation, expected media attention, and complications in reporting process. ◦ Situational factors that encouraged whistleblowing were ethical considerations and professional requirements. |
| **Fredin et al. (2019)** | 263 U.S.-based business professionals (52.5% were female, 43.3% were male, 4.2% did not respond). | Participants were randomly assigned to one of the eight conditions that varied according to scenario type (financial or nonfinancial), moral intensity, and the action of the protagonist (blow the whistle or not). The participants were asked to predict the amount of regret experienced by the protagonist feel if he or she blew the whistle or remained silent. Finally, participants completed questions related to moral intensity. | Quantitative experimental | Unclear | ◦ The regret experienced for whistleblowing is different from the regret for remaining silent. |
| **Fredin (2011)** | 543 business students from a U.S.-based university and 27 business professionals (334 males and 229 females). | In the first block, the participants were presented with a financial scenario that randomly varied in moral intensity. For their scenario, they were asked to predict regret associated with either whistleblowing or remaining silent. The second block repeated the procedure of the first one but with a nonfinancial scenario. The participants were asked to predict the amount of regret experienced by the protagonist feel if he or she blew the whistle or remained silent. After making their prediction, the participants were asked to describe the source(s) of their predicted regret. | Mixed Methods experimental | Unclear | ◦ Individuals are less likely to blow the whistle on financial wrongdoing than they are to blow the whistle on nonfinancial wrongdoing.  ◦ The regret felt by not whistleblowing was different from the regret associated with blowing the whistle on wrongdoing |
| **Gao et al. (2015)** | 369 business students at a university in the U.S. (57% were male). | Participants were presented with two independent case scenarios that varied according to reporting channel available (internal or external), the presence of bystanders (present or absent) and power status of wrongdoer (high or low). The participants were asked to indicate the intentions to blow the whistle. | Quantitative experimental | Unclear | ◦ Whistleblowing intention was higher for third-party administered reporting channel (versus internally administered). ◦ Intentions to blow the whistle using an internal channel decreased when there are other bystanders.  ◦ Whistleblowing intention was lower when the wrongdoer is a superior than when they are peer. ◦ Perceived personal responsibility to report mediated the relationship between whistleblowing intentions and the presence of bystander. ◦ Perceived personal cost moderated the relationship between whistleblowing intentions and power status of wrongdoer. |
| **Grube et al. (2010)** | 330 nurse practitioners (97% were female). | A questionnaire that assessed role identity, frequency of unsafe patient practices, risk-taking propensity, supervisory support for reporting, value dissimilarity, and reporting behavior. | Quantitative observational | Low | ◦ The likelihood of reporting unsafe practices increased as the frequency of unsafe practices increased. ◦ The relationship between frequency of unsafe practices and whistleblowing is moderated by nurse role identity and supervisory support for reporting.  ◦ Strong role identity and strong organizational role identity increased the likelihood of whistleblowing about unsafe practices. |
| **Gundlach et al. (2008)** | 244 non-managerial employees at a U.S.-based company (64% were male). | A questionnaire that consisted of scales that measured perception of intentions, judgment of responsibility, feelings of anger, and whistleblowing decisions. | Quantitative observational | Unclear | ◦ Cognitive responses to wrongdoing can have a significant impact on individuals' decisions to blow the whistle.  ◦ Feelings of anger mediated the relationship between both perceptions of intention and whistle-blowing decisions and judgments of responsibility. |
| **Guthrie and Taylor (2017)** | 295 individuals from the U.S. (50.2% were female). | Participants were presented with a randomly assigned vignette that varied according the presence of retaliation threat (high or low) and the availability of monetary incentive (present or absent). The participants were asked to indicate the likelihood of their reporting the misconduct to the company’s ethics hotline, followed by questions measuring of perceived severity of the wrongdoing, fear of various retaliation types, and organizational trust. | Quantitative experimental | Unclear | ◦ Retaliation threat is negatively associated with whistleblowing intention.  ◦ The relationship between retaliation threat and whistleblowing intentions was mediated by organizational trust.  ◦ Monetary incentives moderated the influence organizational trust. |
| **Gökçe (2013a)** | 180 student teachers at a Turkish university (144 females and 31 males). | A questionnaire that contained a series of scenarios with questions assessing ethical awareness, whistleblowing intentions, and whistleblowing attitudes. | Quantitative observational | Low | ◦ Guaranteed employment increased the likelihood of internal and external whistleblowing. |
| **Gökçe (2013b)** | 107 student teachers at a Turkish university (74 females and 33 males) | A questionnaire that assessed whistleblowing intentions and cultural values | Quantitative observational | High | ◦ No relationship was observed between cultural values and the intentions to blow the whistle externally and anonymously. |
| **Gökçe (2013c)** | 67 Turkish teachers (35 females and 32 males). | A questionnaire that assessed wrongdoings observed, whistleblowing intentions, job satisfaction, and employment commitment. | Quantitative observational | Low | ◦ Males reported wrongdoings significantly more often than females.  ◦ Those individuals with higher seniority (measured in terms of years of experience) reported wrongdoings more often than those with lower seniority. |
| **Gökçe (2013d)** | 291 teachers from Turkey (64% were female). | A questionnaire measuring whistleblowing intentions, ethical value orientation, and cultural orientation. | Quantitative observational | High | ◦ Collectivists preferred to blow the whistle both externally and anonymously. ◦ Individualists preferred report wrongdoings anonymously but not externally. ◦ No relationship was observed between values and the intentions to blow the whistle externally and anonymously. |
| **Gökçe (2013e)** | 163 student teachers at a Turkish university (99 females and 31 males). | A questionnaire that consisted of scenarios with parts of a scale focused on ethical evaluation and whistleblowing intentions. | Quantitative observational | High | ◦ Justice was the strongest influence on whistle-blowing intention. |
| **Gökçe (2015)** | 271 Turkish teachers (62% were female). | A questionnaire that consisted of scales measuring modes of whistleblowing, religiosity, Machiavellianism, and utilitarianism. | Quantitative observational | High | ◦ Religiosity has a positive effect on internal whistleblowing. |
| **Hechanova and Manaois (2020)** | 515 employed at hospitals in Philippines. Phase 1: 12 hospital employees. Phase 2: 503 hospital employees (73% were females). | Phase 1: Interview focused on identified corrupt acts. Phase 2: A questionnaire that measured social desirability, ethical leadership, norms, controls for corruption, attitudes towards corrupt practices, and whistleblowing intentions. | Mixed Methods observational | High | ◦ Policies and systems to control corruption and their implementation were positively related to whistleblowing intentions. ◦ Ethical leadership indirectly influenced whistleblowing intentions through corruption control policies and systems. |
| **Henningsen et al. (2013)** | 174 undergraduate students from a U.S.-based university (53.4% were male). | Participants were presented with a scenario that varied according to wrongdoer's sex (male or female) and whether they are a member of the participant's group for an assignment (yes or no). The participant completed measures related to explicit confrontation, whistleblowing, goals, actions, and planning. | Quantitative experimental | High | ◦ The prevalence of morality and anxiety was positively associated with a greater likelihood to endorse whistleblowing. |
| **Hwang et al. (2008)** | 485 certified public accountants in Taiwan (267 males and 172 females). | A questionnaire that consisted of questions about factors affecting whistleblowing motivations and intentions. | Quantitative observational | Low | ◦ Factors that encouraged whistleblowing were morality and abiding by the policy. ◦ Factors that discouraged whistleblowing were guanxi (personal relationships), fear of retaliation, and fear of media coverage. |
| **Hwang et al. (2014)** | 1095 individuals from certified public accounting firms, corporations, professional associations, and universities in U.S., Taiwan, and China (565 females, 510 males, 20 undisclosed). | A questionnaire that measured whistleblowing intentions, factors that encourage and discourage whistleblowing, and the influence of personal relationships on whistleblowing intentions. | Quantitative experimental | High | ◦ Americans had a higher willingness to engage in whistleblowing than Chinese and Taiwanese. ◦ Whistleblowing intentions of Chinese and Taiwanese were more likely to be influenced by both the dollar amounts involved and the wrongdoing's impact on companies and societies than the Americans. ◦ Personal relationships had a greater effect on the whistleblowing intentions of Chinese and Taiwanese than Americans. |
| **Ion et al. (2015)** | 13 nursing students (9 females and 4 males). | A semi-structured interview. | Qualitative observational | High | ◦ A reason for whistleblowing was that the individual had no choice morally and/professionally.  ◦ Reasons that discourage whistleblowing included the consequences of reporting and situation ambiguity.  ◦ Being adequately prepared for the reality of reporting was a factor that both encouraged and discouraged whistleblowing, |
| **Ion et al. (2016)** | 13 undergraduate students at a university in the UK. | A series of semi-structured interviews focused on how nursing students accounted for their decisions to report or not report poor care witnessed on placement and to propose the potential functions of these accounts. | Qualitative observational | High | ◦ Whistleblowers attributed their behavior to factors such as moral and professional duty and personal disposition. ◦ Non-whistleblowers attributed their behavior to factors such as the hopelessness of the situation, negative personal impact, theory-reality incongruency, and displacement of responsibility. |
| **Izraeli and Jaffe (1998)** | 185 accounting students and professional accountants. | A questionnaire measuring whistleblowing intentions, beliefs and importance of outcomes associated with whistleblowing, subjective norms, locus of control, and professional socialization. | Quantitative observational | Unclear | ◦ Whistleblowing intention was influenced directly by social norms, locus of control, and professional socialization. |
| **Jackson et al. (2010)** | 11 nurse practitioners in Australia who have experiences being a whistleblower. | A semi-structured interview that explored nurses' experiences with whistleblowing. | Qualitative observational | High | ◦ A factor that discouraged whistleblowing was fear of retaliation. ◦ Whistleblowers believed they were acting in accordance with a duty of care |
| **Jenkel and Haen (2012)** | 44 undergraduate business and accounting students (28 males and 16 females). | Participants completed a math computation test without the reliance of calculators under a time constraint. During the task, a confederate used calculator near the end. After the test, the participants completed a survey which includes the opportunity to report the wrongdoing either anonymously or non-anonymously (depending on the condition). | Quantitative experimental | High | ◦ Personal impact of wrongdoing increases the likelihood of whistleblowing. |
| **Jones et al. (2014)** | 412 undergraduate accounting students (232 males and 182 females). | A questionnaire that presented four versions of a scenario with questions regarding perceived unfairness, anger, and self-interest in the situation. | Quantitative experimental | Low | ◦ Both anger and perceived unfairness were positively associated with likelihood of whistleblowing. |
| **Kamarunzaman et al. (2014)** | 511 public office employees from Malaysia (296 females and 215 males). | A questionnaire that assessed perceived organizational support, channels of communication, attitudes of whistleblowing, and whistleblowing intentions. | Quantitative observational | High | ◦ Whistleblowing intention was positively associated with perceived organizational support, attitudes towards whistleblowing, and channel of communication. ◦ An influential factor on whistleblowing intentions was channel of communication. |
| **Kaplan et al. (2009)** | 113 MBA students (70% were male) | A questionnaire comprised of a scenario that varied according to the wrongdoer's sex (male or female). The participants were asked about their whistleblowing intentions, perceptions of the fraudulent act, personal costs, and responsibilities of reporting. | Quantitative experimental | Unclear | ◦ Females' whistleblowing intentions through an anonymous reporting channel are higher than for males; but there is no difference in terms of non-anonymous reporting channels. ◦ Relative decreases in the perceived personal costs resulted in stronger whistleblowing intentions. ◦ The availability of an anonymous reporting channel increased whistleblowing intentions. |
| **Kaplan (1995)** | 57 audit seniors of an internationally certified public accountant firm (42% were female). | Participants was presented with one of four versions of a scenario that varied according to the necessity of the missed step and quality of previous work. The participants were asked to indicate their whistleblowing intentions, perception of overall performance rating, and team support. | Quantitative experimental | Unclear | ◦ Whistleblowing intentions were higher when a necessary audit step was missed. ◦ Poor staff audit work history increased whistleblowing intentions. |
| **Kaplan et al. (2009)** | 120 M.B.A. students from a university.  Main study: 91 M.B.A. students from a university (38% females).  Ancillary study: 29 M.B.A. students from a university. | Main Study: Participants were presented with a scenario that was manipulated on the presented procedural safeguards (strong or weak) and type of fraudulent act (misappropriation of assets or fraudulent financial reporting). The participants were asked to indicate the likelihood of whistleblowing, perceptions of the fraudulent act, and potential consequences of whistleblowing. Ancillary study: Participants were presented with a scenario that was manipulated on the whistleblowing hotline administrator (internal or external), while indicating whistleblowing intentions. | Quantitative experimental | Unclear | ◦ Anonymous whistleblowing intentions were higher for fraudulent act involving misappropriation of assets than one involving fraudulent financial reporting. ◦ Anonymous whistleblowing intentions were higher under the weak condition of procedural safeguards. ◦ Whistleblowing intentions were higher for an internally administered hotline. |
| **Kaplan et al. (2010)** | 77 M.B.A. students at a university (33% were female) | Participants were presented with a scenario that varied according to the type of fraudulent act (misappropriation of assets or fraudulent financial reporting) and social confrontation (present or absent). The participants were asked to indicate whistleblowing intentions. | Quantitative experimental | Unclear | ◦ When social confrontation with wrongdoing superior was unsuccessful, whistleblowing intention to the supervisor's supervisor was higher than to an internal auditor. ◦ When social confrontation with superior did not occurred, whistleblowing intention to the internal auditor was higher than to the supervisor’s supervisor. |
| **Kaplan et al. (2011)** | 207 M.B.A. students from a university (40% were female). | Participants were presented with one of eight scenarios that varied according to type of auditor (internal or external), existence of an inquiry (present or absent), and type of fraudulent act (misappropriation of assets and fraudulent financial reporting). The participants were asked to indicate the likelihood of whistleblowing. | Quantitative experimental | Unclear | ◦ Intentions to blow the whistle to an inquiring auditor was higher than the reporting intentions to a non-inquiring auditor. ◦ Intentions to blow the whistle to an internal auditor was higher than to an external auditor. |
| **Kaplan and Schultz (2007)** | 73 M.B.A. students from a university. | Participants were presented with three different scenarios while being asked to indicate their whistleblowing intentions. | Quantitative experimental | Unclear | ◦ The availability of an anonymous channel reduced the likelihood of whistleblowing to non-anonymous channels. ◦ The setting of potential wrongdoing influenced the channel used by the whistleblower. |
| **Kaplan and Whitecotton (2001)** | 73 audit seniors from a large CPA firm. | Participants were presented with one of four scenarios that varied according to inherent risk (high or low) and professional integrity (high or low). The participants were asked to indicate the seriousness of the wrongdoing, personal responsibility of reporting, personal cost of reporting, and whistleblowing intentions. | Quantitative experimental | Unclear | ◦ Whistleblowing intentions were negatively association with perceived personal costs of reporting. ◦ Whistleblowing intentions were positively associated with perceived personal responsibility for reporting. |
| **Kaptein (2011)** | 5065 U.S.-based employees (44% were female). | A questionnaire that assessed responses to wrongdoings and the ethical culture of their employers. | Quantitative observational | Low | ◦ Internal whistleblowing (in the form of calling an ethics hotline) was positively associated with clarity, congruency of senior management, feasibility, supportability, and sanctionability, but negatively related to congruency of local management, and discussability. ◦ External whistleblowing was positively related to feasibility and transparency but negatively associated with clarity, congruency of local and senior management, discussability, and sanctionability. |
| **Keenan (1995)** | 406 first-level managers (89% were male). | A revised version of the 1980 U.S. Merit Systems Protection Board survey. | Quantitative observational | Low | ◦ Being obliged to blow the whistle was positively related to moral perceptions of minor fraud, harm to other, and serious fraud, along with knowledge or information of where to report, and managerial experience. ◦ Being obliged to blow the whistle was negatively related to fear of retaliation low and reprisal. ◦ Females were less likely to feel obliged to blow the whistle. |
| **Keenan (2000)** | 725 upper- (n = 131; 88% were male), middle- (n = 188; 91% were male), and lower-level managers (n = 406; 89% were male). | A questionnaire that measured moral perception, whistleblowing intentions, personal and organization propensity of whistleblowing behaviors, interpersonal communication climate, and the fear of retaliation. | Quantitative observational | Low | ◦ Organizational and individual propensity positively influenced the likelihood of blowing the whistle on less serious forms of fraud by all managerial levels. ◦ Moral perceptions positively influenced the likelihood of blowing the whistle on less serious forms of fraud of upper- and lower-level managers but negatively influenced middle-level managers. ◦ Upper- and middle-level managers were more likely to blow the whistle on less serious fraud. |
| **Keenan (2002a)** | 76 managers from large manufacturing firms in the U.S. (n = 45; 100% were male) and India (n = 31; 100% were male) | A questionnaire that measured moral perception, whistleblowing intentions, personal and organization propensity of whistleblowing behaviors, and the fear of retaliation. | Quantitative observational | Low | ◦ No difference between American and Indian managers in terms of whistleblowing likelihood. |
| **Keenan (2002b)** | 725 upper- (n = 131), middle- (n = 188), and lower-level managers (n = 408). | A questionnaire that measured moral perception, whistleblowing intentions, personal and organizational propensity of whistleblowing behaviors, interpersonal communication climate, and the fear of retaliation. | Quantitative observational | Low | ◦ Upper-level managers were more likely to blow the whistle on serious, minor, and harm to others forms of wrongdoing, followed by middle-level and then lower-level managers. |
| **Keenan (2007)** | 145 mid-level managers from large companies in the U.S. (n=70) and People’s Republic of China (n=75). | A revised version of the U.S. Merit Systems Protection Board survey questionnaire. | Quantitative observational | High | ◦ Chinese managers are less likely to blow the whistle than their American counterparts. |
| **Keil et al. (2007)** | 146 students at universities in the U.S. (n = 68; 37 females and 31 males) South Korea (n = 78; 31 females and 47 males) | Participants were presented with one of two scenarios that varied according to the opportunity to shift blame (present or absent). The participants were asked to indicate their likelihood to report bad news and the reason underlying the decision. | Mixed Methods experimental | Unclear | ◦ The presence of a blame-shifting opportunity increased the willingness to report bad news among U.S. participants, but not South Korean participants. ◦ Reasons that influenced willingness to report include a small chance of success, severity of consequences, role responsibility, reputation (personal and company), desire to fix the issue, honesty, and desire to avoid surprises. |
| **Keil et al. (2018)** | 414 individuals. Study 1: 136 undergraduate business school students from a U.S.-based university (91 males and 45 females). Study 2: 135 undergraduate business school students from a U.S.-based university (103 males and 32 females). Study 3: 143 professionals in the health and pharmaceuticals (117 females and 26 males). | Study 1: A questionnaire comprised of a scenario that varied according to the intentionality of the wrongdoing and the stability of wrongdoing. Study 2 and 3: A questionnaire comprised of a different scenario, but also varies on intentionality and stability. Participants in all studies were asked are questions assessing seriousness of the wrongdoing, anticipated regret about remaining silent, and whistle‐blowing intention. | Quantitative experimental | Unclear | ◦ The effect of the seriousness of a wrongdoing on whistle‐blowing intentions was mediated by anticipated regret about remaining silent. ◦ Committing a wrongdoing intentionally increased the likelihood of both experiencing of anticipated regret about remaining silent and whistleblowing intention.  ◦ Committing a wrongdoing unintentionally engage in wrongdoings decreased the likelihood of experiencing anticipated regret about remaining silent and whistleblowing intentions. |
| **Keil et al. (2004)** | 122 students from two U.S.-based universities (26.4% were female). | Participants were presented with one of four scenarios that varied according to information symmetry (high or low) and organizational climate (conducive or non-conducive to reporting). The participants were asked to indicate their level of reluctance to report bad news, whether something ought to be reported, perceived personal responsibility for reporting, perceived information asymmetry, and perceived organizational climate. | Quantitative experimental | Unclear | ◦ The assessment of personal responsibility to report had a direct positive effect on the willingness to report bad news. ◦ The assessment of whether the status ought to be reported had an indirect positive influence on reporting bad news through the assessment of personal responsibility to report. ◦ Information asymmetry and organizational climate exerted their influence on the reluctance to report through perceptions of whether something ought to be reported and personal responsibility, respectively. |
| **Keil et al. (2010)** | 132 individuals (44% were female). | A questionnaire consisting of scenarios that varied on personal reporting responsibility, trust in supervisor, ability to hide information, reporting anonymity, management responsiveness, organizational climate conduciveness, and senior management attachment to project. The participants were asked to indicate their whistleblowing intentions for each scenario. | Quantitative observational | High | ◦ Whistleblowing intentions were positively associated with trust in supervisor, management responsiveness, organizational climate conduciveness, and senior management attachment to project.  ◦ Perceived benefit-to-cost differential associated with whistleblowing positively mediated the relationship between whistleblowing intentions and trust in supervisor, management responsiveness, organizational climate conduciveness, personal reporting responsibility, and reporting anonymity. |
| **Kennett et al. (2011)** | 81 accounting students from a university in the U.S. (65.4% were female). | Participants were presented with a scenario with questions that assessed peer approval, moral obligation, personal financial costs, whistleblowing likelihood. | Quantitative observational | High | ◦ Peer approval and societal benefits were positively associated with whistleblowing intentions. ◦ Personal financial cost was negatively related to whistleblowing intentions. |
| **King (1997)** | 261 registered nurses (96% were female). | A questionnaire comprised of scenarios that varied according to the severity of the incident and interpersonal closeness with the wrongdoer. Participants were asked about their likelihood to report a wrongdoing. | Quantitative experimental | Unclear | ◦ Interpersonal closeness between an observer and wrongdoer influenced the likelihood of report a wrongdoing. ◦ Severity of incident influenced the likelihood of whistleblowing. ◦ Severity of the wrongdoing influenced the relationship between interpersonal closeness and likelihood of whistleblowing. |
| **King and Hermodson (2000)** | 197 registered nurses in the U.S. (94% were female). | A questionnaire containing open-ended questions asking participants to detail an incident of wrongdoing and the reasons for reporting or not reporting the incident. | Qualitative observational | High | ◦ Reasons for whistleblowing included personal ethics and the belief that reporting was the "right thing to do", patient rights violation, harm to patient (potential and actual), patient negligence, legal issues, policies and procedures, and personnel issues.  ◦ Reasons for not whistleblowing included personal fear, incidents were not observed personally, commonly accepted wrongdoing, wrongdoing was handled through group efforts, issue was minor, perceived or actual retaliation, and reception problems on the part of authority figures or administration. |
| **King and Scudder (2013)** | 68 nurses (66 females and 2 unidentified). | A survey that consisted of questions addressing the reasons to report and not report wrongdoings | Quantitative observational | High | ◦ Patients’ well-being and professional ethics were the main reasons for deciding whether or not to report a wrongdoing. |
| **Latan et al. (2019a)** | 162 external auditors and 173 internal auditors in Indonesia (63.28% were male). | A questionnaire comprised of scenarios and questions related to whistleblowing intentions, ethical awareness, ethical judgment, perceived moral intensity, and emotion. | Quantitative observational | Low | ◦ Ethical judgment had a positive direct effect on whistleblowing. ◦ Emotion and perceived moral intensity improved the relationship between ethical judgments and whistleblowing intentions.  ◦ Internal auditors were more likely to blow the whistle than external auditors. |
| **Latan et al. (2019)b)** | 157 tax service employees in Indonesia (123 males and 34 females). | A questionnaire comprised of a scenario and questions assessing perceived seriousness of threat, perceived seriousness of wrongdoing, rationalization, and whistleblowing intentions. | Quantitative observational | Low | ◦ Perceived seriousness of threat reduced intention to blow the whistle. ◦ Perceived seriousness of wrongdoing increased whistleblowing intentions. ◦ Rationalization moderated the relationships between perceived seriousness of threat, perceived seriousness of wrongdoing, and whistleblowing intention. |
| **Latan et al. (2019)c)** | 223 professional accountants from auditing firms in Indonesia (159 males and 64 females). | A questionnaire comprised of a scenario and questions assessing whistleblowing intentions along with the pressures, financial incentives, opportunities, and rationalization experienced when discovering misconducts. | Quantitative observational | Low | ◦ The higher the pressure experienced, the greater the chances that a person will not blow the whistle.  ◦ Financial incentives, opportunities, and rationalization had a positive effect on whistleblowing intentions. |
| **Latan et al. (2018)** | 256 Indonesian public accountants (61.6% were male) | A questionnaire comprised of a scenario and questions measuring perceived behavioral control, attitudes towards whistleblowing, independence commitment, personal responsibility of reporting, personal costs of reporting, perceived organizational support, team norms, perceived moral intensity, and whistleblowing intentions. | Quantitative observational | Low | ◦ Whistleblowing intentions were influenced positively by attitudes toward whistleblowing, perceived behavioral control, independence commitment, and personal responsibility for reporting ◦ Personal cost of reporting had a negative influence on whistleblowing intentions.  ◦ Perceived organizational support, team norms, and perceived moral intensity can partially improve the relationship between the individual-level low antecedents and whistleblowing intentions. |
| **Lavena (2016)** | 36,926 U.S. federal government employees. | 2005 Merit Systems Protection Board Merit Principles Survey | Quantitative observational | Low | ◦ Whistleblowing likelihood was positively associated with norm-based and affective work motives. ◦ Whistleblowing likelihood was negatively related perceptions of respect and openness, cooperativeness and flexibility in the work setting, and fair treatment and trust in superiors. |
| **Lee et al. (2004)** | 1952 U.S. federal government employees (100% were female). | A questionnaire on sexual harassment. | Quantitative observational | High | ◦ Whistleblowing was positively to number of types of both felonious and non-felonious sexual harassment, frequency and length of sexual harassment, and multiple harassers  ◦ Whistleblowing was negatively associated with organizational level of the harasser. |
| **Li and Ma (2016)** | 120 accountancy students at a university in Hong Kong (82 females and 38 males). | Initially, participants completed a questionnaire containing scenarios and questions related to ethical judgment and whistleblowing intentions. This was followed by small group discussions with other participants (3-4 individuals) about the same vignettes and questions. After the group discussion, the participants completed the questionnaire independently. | Quantitative experimental | Unclear | ◦ The group whistleblowing responses were more likely to endorse stronger actions than individual responses. |
| **Lim and See (2001)** | 518 students from educational institutions in Singapore (383 females and 135 males). | A questionnaire that measured self-reported cheating, perceived seriousness of cheating, perceived prevalence of cheating, and willingness to report cheating. | Mixed Methods observational | High | ◦ Reasons for not blowing the whistle included the observer having an indifferent attitude of the wrongdoing and a self-interested motivation of preventing future reporting if the observer commits a wrongdoing. |
| **Liu and Ren (2017)** | 150 trainee auditors from a certified public accounting firm in China. | A questionnaire comprised of scales measuring whistleblowing intentions, perceptions of team leader's ethicality, likelihood of reporting, firm size, and client's irregularities. | Quantitative observational | Unclear | ◦ Perceived ethicality of team leader was positively related to the likelihood of whistleblowing on client’s financial frauds. ◦ Higher evaluation of the stable firm–client relationship reduced the likelihood of reporting client’s irregularities. ◦ Concerns regarding future career development increased the likelihood of whistleblowing.  ◦ Negative association between team size and the likelihood of reporting client’s financial frauds. |
| **Liu et al. (2015)** | 718 supervisors (n = 121; 90.1% were male) and employees (n = 597; 81.4% were male) of a telecommunications company in China. | A two-part questionnaire that measured authentic leadership, psychological safety, personal identification, and whistleblowing behavior. | Quantitative observational | High | ◦ Authentic leadership is positively related to internal whistleblowing.  ◦ Both team psychological safety and personal identification partially mediated the relationship between authentic leadership and internal whistleblowing. |
| **Liu et al. (2016)** | 559 employees in China (49.5% were female). | A questionnaire comprised of scales assessing proactive personality, instrumental ethical climate, moral identity, and whistleblowing intentions. | Quantitative observational | Unclear | ◦ Proactive personality was positively related to internal whistleblowing intention.  ◦ Both Instrumental ethical climate and moral identity positively influence the relationship between proactive personality and external whistle-blowing intention. |
| **Liyanarachchi and Newdick (2009)** | 51 undergraduate accounting students from a university in New Zealand (28 males and 23 females). | A questionnaire comprised of a task that varied according retaliation consequences (strong or weak) and questions assessing whistleblowing likelihood and moral reasoning scale. | Quantitative experimental | Unclear | ◦ Individuals with higher levels of moral reasoning are more likely to blow the whistle than those with lower levels.  ◦ Whistleblower's sex influences the relationship moral reasoning and whistleblowing intentions. Specifically, the effect of moral reasoning on whistleblowing intentions was greater for female participants.  ◦ Experiencing weaker levels of retaliation for whistleblowing increased the likelihood of whistleblowing. |
| **Liyanarachchi and Adler (2011)** | 98 accountants in Australia (39 females, 50 males, 9 unidentified). | Participants were randomly assigned to one of two conditions that varied retaliation level (weak or strong). The participants were presented with hypothetical scenarios with questions focused on whistleblowing likelihood. | Quantitative experimental | Unclear | ◦ For accountants aged 25 to 34 years, males were more likely to blow the whistle.  ◦ For accountants between 35 and 44 years of age, they were not only more likely to blow the whistle when there was retaliation but tend to be more willing to do so when the retaliation involved a direct personal loss.  ◦ For accountants that were 45 years or older, females' whistleblowing intentions declined as the retaliation threat increased. As for males, changes in retaliation threat had little impact on their whistleblowing intentions. |
| **Lowe et al. (2015)** | 54 M.B.A. students from a university in the U.S. (31% were female). | Participants were presented with a scenario that varied according to sub-certification of financial results (present or absent) and fraud discovery timing (before filing or after filing). The participants were asked to indicate the whistleblowing intentions, personal responsibility, management responsiveness, seriousness, general perceptions of the wrongdoing, accountability, and personal cost. | Quantitative experimental | Unclear | ◦ The presence of a sub-certification process reduced whistleblowing intentions.  ◦ Personal responsibility partially mediated the relationship between sub-certification and whistleblowing intentions. |
| **Lyndon et al. (2012)** | 120 obstetricians and registered nurses at two U.S.-based hospitals (108 females and 6 males). | A questionnaire comprised of scenarios and questions assessing potential harm and likelihood of speaking up, along with measures of safety climate, teamwork climate, disruptive behavior, work stress, and personality traits of bravery and assertiveness. | Quantitative observational | Low | ◦ Communication quality was positively related to the likelihood to speaking up about potential harm to patients. ◦ Exposure to disruptive behavior, frequent exposure to rudeness and intimidation, and feeling intimidated by physicians were negatively related to the likelihood of speaking up. ◦ After controlling for bravery and assertiveness, a higher perception of harm, respondent role, specialty experience, and location predicted the likelihood of speaking up. |
| **MacGregor and Stuebs (2014)** | 79 postgraduate accounting students from a U.S.-based university (47% were male). | A questionnaire comprised of scenarios with questions focused on assessing whistleblowing attitudes. | Quantitative observational | Unclear | ◦ Awareness, community, and moral competence dimensions most influence the willingness to remain silent. ◦ The importance of these factors is associated with the severity of the rule-violation. Awareness and moral competence are more important factors with clear, severe rule violations. Community influences and personal concerns become more important for less severe and more ambiguous rule violations. |
| **MacNab et al. (2007)** | 1187 participants from Canada (n = 476; 40% were female), Mexico (n = 248; 40% were female) and the U.S. (n = 463; 43% were female). | A questionnaire that measured culture and whistleblowing intentions. | Quantitative observational | Low | ◦ Uncertainty avoidance was positively related to both internal reporting and whistleblowing. ◦ High power distance discouraged internal reporting among U.S. and Canadian individuals, but not Mexican individuals. |
| **MacNab and Worthley (2008)** | 939 individuals from Canada (n = 476; 40% were female) and the U.S. (n = 463; 43% were female). | A questionnaire that measured self-efficacy and internal whistleblowing propensity. | Quantitative observational | Low | ◦ Self-efficacy influenced the propensity to blow the whistle internally. |
| **Mbago et al. (2018)** | 127 staff of procuring and disposing entities in Uganda (33% were female). | The quantitative data was collected using a questionnaire assessing legitimacy and whistleblowing intentions. The qualitative data was collected through an in-depth interview. | Mixed Methods observational | Unclear | ◦ Legitimacy is a significant predictor of whistleblowing intentions and behavior. |
| **McCutcheon (2006)** | 77 employed adults from the U.S. (35 females, 42 males, and 1 unidentified). | This study was divided into two parts. First, the participants completed a survey containing scales assessing compliance towards supervisor directives, submissiveness to authority, self-righteousness, social desirability, and whistleblowing. Second, the participants watched three videos and answered questions about the ethicality of the observed behaviors. | Quantitative observational | Unclear | ◦ None of the personality scales predicted whistleblowing intentions. |
| **McManus et al. (2012)** | 156 accounting students from two Australian universities (80 females, 61 males, and 15 unidentified) | The participants completed either an internet-based or in-class textbook ethics instruction. At the end, they completed a questionnaire containing whistleblowing-related ethical dilemmas and questions measuring decision-making, ethical judgement, moral equity, relativism, and contractualism. | Quantitative experimental | High | ◦ Exposure to a web-based ethics instruction module increased the likelihood of whistleblowing when compared the traditional in-class textbook ethics instruction approach. |
| **Miceli et al. (1991)** | 653 Directors of Internal Auditing in North America (86.3% were male). | A questionnaire that assessed the details pertaining to an observed wrongdoing, along with pay satisfaction, personal job performance, moral reasoning, and organizational commitment, and bureaucratic climate. | Quantitative observational | High | ◦ Potential whistleblowers may not report wrongdoings if they had negative evaluation of their own job performance, received a lower salary, or perceived their employer as highly bureaucratic.  ◦ The likelihood of whistleblowing externally increased when there was perceived harm to the public or a peer by the wrongdoing, the wrongdoing involved theft by relatively low-level employee, there were few other observers, or the organization was highly regulated. |
| **Miceli et al. (2012)** | 3288 military and civilian employees from a US military base. | A questionnaire that assessed perceived organizational support, perceptions of the distributive and procedural justice of whistle-blowing channels, and prosocial personality. | Quantitative observational | Low | ◦ Whistleblowing is positively associated with strength of evidence, observer leverage, and proactive personality ◦ Whistleblowing is negatively associated with co-worker invalidation of wrongdoing for those employees who observe uncorrected or unreported wrongdoing. |
| **Miceli et al. (1991)** | 295 students at a U.S.-based university (140 females and 155 males). | This experiment consisted of two parts. During the first part, the participants completed a questionnaire that measured their locus of control and moral judgment. For the second part, the participants were asked to complete a task. However, prior to its administration, the participants were asked informally by a confederate to adjust their responses to fit the hypothesis. The content of this message varied according to the threatened retaliation (high or low). After the completion of the task, a questionnaire was administered by another confederate asking the participants to indicate their whistleblowing intentions and conformity. | Quantitative experimental | Unclear | ◦ Males were more likely than women to blow the whistle.  ◦ Individuals with lower moral development scores were more likely to blow the whistle than those with higher scores.  ◦ Whistleblowing behavior occurred when there were more observers of wrongdoing, versus few observers. |
| **Miceli and Near (1984)** | 8587 U.S. federal government officials. | The 1980 US Merit Systems Protection Board survey. | Quantitative observational | Low | ◦ Those individuals who blow the whistle internally were more likely to be highly educated supervisors and/or employees in positions where whistleblowing was role-prescribed, had a strong approval of whistleblowing, more likely to report that knowing others would not think badly of them would encourage them to blow the whistle, and were less convinced that anonymity would encourage them to blow the whistle than were other employees. ◦ External whistleblowers were less likely to be in supervisory positions, less likely to believe that anonymity, approval of other organization members, and financial incentives would encourage them to blow the whistle, believed that they had more knowledge of where to report wrongdoing, were more likely to believe that knowing the organization would take action on their complaint would motivate them, and were more approving of whistleblowing than were other organization members. |
| **Miceli and Near (1985)** | 8587 U.S. federal government employees. | The 1980 US Merit Systems Protection Board survey, plus archival data and aggregate measures of organizational climate. | Quantitative observational | Low | ◦ Strength of evidence, serious wrongdoings, being personally affected by the wrongdoing increased the likelihood of whistleblowing. ◦ For wrongdoings that were serious, the organization depended upon, and threatened with retaliation, observers were more likely to blow the whistle externally. |
| **Miceli and Near (1988)** | 4897 U.S. federal government employees. | The 1983 US Merit Systems Protection Board survey. | Quantitative observational | Low | ◦ Higher level professional staff were more likely to blow the whistle. ◦ An observer with a more positive job response was more likely to blow the whistle. ◦ Males were more likely to blow the whistle. ◦ Whistleblowers were more likely to members of larger work groups. ◦ Organizational responsiveness was positively associated with whistleblowing. |
| **Milliken et al. (2003)** | 40 M.B.A. students from a university in the U.S. | An in-depth interview exploring the level of comfort speaking with a superior and others about problems or issues in their current employer. | Qualitative observational | High | ◦ Reasons that discouraged whistleblowing were the fear of being labelled or viewed negatively, fear of damaging a relationship, feelings of futility, fear of retaliation or punishment, concerns about the negative impact on others, lack of experience and/or tenure, organizational characteristics (hierarchical structure and unsupportive culture), and a poor relationship with superior. |
| **Moore and McAuliffe (2010)** | 152 nurses in Ireland. | A questionnaire that assessed experiences and attitudes to reporting poor care. | Quantitative observational | Unclear | ◦ The major factors discouraging whistleblowing fears of retribution, not wanting to cause trouble, not being sure if reporting an incident is the right thing to do, and hurting a colleague. |
| **Moore and McAuliffe (2012)** | 152 nurses in Ireland. | A questionnaire that assessed experiences and attitudes to reporting poor care. | Quantitative observational | Unclear | ◦ The major factors discouraging whistleblowing fears of retribution, not wanting to cause trouble, and not being sure if reporting an incident is the right thing to do. |
| **Nawawi and Salin (2018)** | 63 employees of a large company (35 females and 28 males). | A questionnaire that consisted of scenarios and questions that measured whistleblowing intentions. | Quantitative observational | High | ◦ Executive-level employees were more likely to blow the whistle than those a non-executive position. ◦ Seriousness of the wrongdoing was positively related to whistleblowing intentions. |
| **Nayir et al. (2018)** | 732 private (n = 327; 64.2% were male) and public sector (n = 405; 79.5% were male) employees in Turkey. | A questionnaire that measured ethical values and whistleblowing intentions. | Quantitative observational | Low | ◦ Idealism was positively related to internal whistleblowing and negatively associated with anonymous whistleblowing intentions. ◦ Relativism was positively related to external and anonymous whistleblowing intentions. ◦ More relativistic private sector employees were more likely to prefer external whistleblowing and less likely to prefer internal whistleblowing. |
| **Near et al. (1993)** | 8587 U.S. federal government employees | 1980 U.S. Merit Systems Protection Board Survey | Quantitative observational | Unclear | ◦ Knowledge about whistleblowing channels was positively related to the incidence of whistleblowing. |
| **Near et al. (2004)** | 3288 employees of a military based in the U.S. | A questionnaire that assessed types and characteristics of wrongdoings observed, whistleblowing behaviors, reasons for not reporting, and retaliation experienced. | Quantitative observational | High | ◦ Observed wrongdoings involving mismanagement, sexual harassment, or unspecified legal violations were more likely to blow the whistle than those that involved stealing, waste, safety problems, or discrimination.  ◦ Primary reason for discouraging whistleblowing was that the observers thought nothing could be done to rectify the situation. |
| **Nurhidayat and Kusumasari (2019)** | 5 whistleblowers along with document and regulations pertaining to whistleblowing in Indonesia. | In-depth interviews and a document analysis | Qualitative observational | High | ◦ The attitude of whistleblowers is an important factor influenced whistleblowing intention or action. ◦ Self-integrity, self-confidence, professionalism, and passion for one’s occupation were factors that influenced whistleblowing |
| **Oelrich (2019)** | 39 university students (48.7% were female). | A multistage experiment where the participants had to decide on the monetary value (i.e., payoff) that would make the two presented choices indifferent. At one stage of the experiment, the participants were asked for their whistleblowing intentions with respects to answer provided previously. | Quantitative experimental | Unclear | ◦ Whistleblowing intentions is more strongly decreased by monetary losses than increased by monetary gains. |
| **Olesen et al. (2019)** | 21 researchers from universities in Malaysia. | A semi-structured interview focused on experiences with research misconduct. | Qualitative observational | High | ◦ Factors discouraging whistleblowing were the lack of protection of whistleblower’s identity, fear of retaliation, close relationship with wrongdoer, the tedious investigative process, and the notion of avoiding confrontation. |
| **Omotoye (2017)** | 22 employees from a district council in Botswana. | A multi-methods questionnaire that assessed whistleblowing perceptions and reporting of corrupt activities. | Mixed Methods observational | High | ◦ Factors encouraging whistleblowing were identity protection, protected from retaliation, if the wrongdoing posed a threat to people’s lives or suppresses social justice. ◦ Factors discouraging whistleblowing were absence of a whistleblowing policy that protects whistle-blowers, fear of losing job, and fear of punishment. |
| **Park et al. (2008)** | 159 students from a U.S.-based university (55% were female) | Participants were presented with one of four scenarios that varied according to which party was responsible for the fault (external or internal) and time urgency (high or low). The participants were asked questions that willingness to blow the whistle, perceived fault responsibility, and perceived time urgency. | Quantitative experimental | Unclear | ◦ The assessment of personal responsibility to report had a direct positive effect on the willingness to report bad news. ◦ The assessment of whether the status ought to be reported had an indirect positive influence on reporting bad news through the assessment of personal responsibility to report. ◦ Fault responsibility exerted both direct and indirect influence on willingness to report bad news,  ◦ Time urgency exerted an indirect influence on willingness to report bad news. |
| **Park and Keil (2009)** | 250 students at a university in the U.S. (45% were female). | Participants were presented with one of eight scenarios that varied according to the organizational structures/policies (conducive or non-conducive), managerial communication practices (open or closed), and the degree of demographic dissimilarity between employees and top managers (similar or dissimilar). The participants were asked to indicate their perceptions of the climate of silence, whistleblowing intentions, whether negative information should be reported, and personal responsibility to reporting information. | Quantitative experimental | Unclear | ◦ Climate of silence influenced whistleblowing intentions directly and indirectly (through the assessment of whether wrongdoing should be reported and personal responsibility to report). ◦ Organizational structures/policies, managerial practices, and degree of demographic dissimilarity between employees and top managers influenced whistleblowing intentions through climate of silence. |
| **Park et al. (2009)** | 155 undergraduate students at a U.S.-based university (55% were female). | In one of the four conditions, the participants were presented with a scenario that varied on the scope of impact (high vs low) and type of impact (bodily harm vs financial loss), then asked about their willingness to report bad new, perception of whistleblowing impact, whether the information ought to be reported, personal responsibility of reporting, personal morality, and willingness to communicate. | Quantitative experimental | Unclear | ◦ The perceived impact of wrongdoing indirectly influenced willingness to blow the whistle through the assessment of whether the status ought to be reported ◦ Both morality and personal responsibility to report directly affected whistleblowing intentions.  ◦ Willingness to communicate had no effect on whistleblowing willingness. |
| **Park and Blenkinsopp (2009)** | 296 police officers in South Korea (217 males and 79 females). | A questionnaire that assessed whistleblowing intentions, attitudes, subjective norms, and perceived behavioral control. | Quantitative observational | High | ◦ Attitudes towards whistleblowing, subjective norms, and perceived behavioral control affected internal whistleblowing intentions. ◦ Subjective norms affected external whistleblowing intentions. |
| **Park et al. (2005)** | 343 public officials in South Korea (67% were male). | A questionnaire that measured whistleblowing attitudes and intentions, Confucian ethical beliefs, individualism, and collectivism. | Quantitative observational | Low | ◦ Confucian ethics had an influence on internal and external whistleblowing intentions. Specifically, father-son affection had a negative effect on intentions, while the distinction between husband and wife roles had a positive effect on intentions. ◦ Horizontal collectivism had positive effects on internal and external whistleblowing. |
| **Perry et al. (1997)** | 434 members of an organization focused on furthering education in Women's Studies (426 females and 8 males). 8523 employees of U.S. federal government. | One instrument was a questionnaire presenting a sexual assault scenario with questions assessing power position, personal power, and organizational efficacy. Another instrument in this study was the 1988 U.S. Merit Systems Protection Board survey. | Quantitative observational | Low | ◦ Position power and personal power of the victim and the organization's efficacy at handling with previous incidents were positively associated with the whistleblowing intentions. |
| **Pillay et al. (2012)** | 250 senior, middle and lower-level management/administration personnel from government agencies in South Africa (73 females and 177 males). | A questionnaire that measured the influence of factors on whistleblowing, perception of value to organization, and personal relationships in an organization. | Quantitative observational | Low | ◦ Factors encouraging whistleblowing were professional ethics, personal morality and obedience to organizational policy. ◦ Factors that discouraged Factors that discourage whistleblowing were a strong legal, a strong internal control system, family priorities, and risk aversion. ◦ The societal impact of the wrongdoing increased the likelihood of whistleblowing. ◦ Perceptions of positive organizationalvalues positively affected whistleblowing intention. |
| **Pillay et al. (2018)** | 387 national level of government services employees in South Africa (n=250; 177 males and 72 females) and Mauritius (n=137; 93 males and 44 females). | A questionnaire that assessed cultural values, ethics and morality, cultural reluctance, fear of retaliation, confidence in organizational systems, and whistleblowing intentions. | Quantitative observational | Low | ◦ Factors discouraging whistleblowing include morality and ethics, fear of retaliation, levels of faith in organizational reporting systems, and an uncertainty avoidance cultural reluctance. ◦ Barriers to whistleblowing were stronger in Mauritius than South Africa. |
| **Pillay et al. (2017)** | 200 employees of government organizations in South Africa (103 females and 97 males). | A questionnaire that measured whistleblowing intentions, perceptions of personal relationships with the organization, and the relationship between culture and whistleblowing. | Quantitative observational | High | ◦ Factors that encouraged whistleblowing were that the wrongdoing violated personal morality or social justice, and that whistleblowing behavior was consistent with organizational policy. ◦ Factors that discouraged whistleblowing were a limited amount of available time, existence of a strong legal system, fear of retaliation, fear of media attention, loyalty, risk aversion, high power distance, uncertainty avoidance, and value success over quality of life. |
| **Pope and Lee (2013)** | 97 MBA students at U.S.-based university (51% were male). | In one of the four conditions, the participants were presented with a scenario that varied on the reporting channel (anonymous vs non-anonymous) and financial reward (yes vs no). The participants were asked to indicate their whistleblowing intentions. | Quantitative experimental | Unclear | ◦ The existence of a financial reward increased whistleblowing intentions. ◦ The availability of an anonymous reporting channel does not affect whistleblowing intentions. |
| **Previtali and Cerchiello (2018)** | 365 reports of public administrations from hospitals, health agencies, universities, and municipalities in Italy. | Annual reports submitted to the National Anti-Corruption Agency. | Quantitative observational | Unclear | ◦ The presence of a proceduralized system increased the likelihood of whistleblowing. |
| **Proost et al. (2013)** | 278 employees in organizations in the Slovak Republic (67% were female). | A questionnaire comprised of a scenario with questions assessing whistleblowing intentions, supervisor procedural fairness, and moral identity. | Quantitative observational | Low | ◦ Moral intensity was positively associated with whistleblowing intentions. ◦ High supervisor procedural fairness influenced the relationship between moral intensity and whistleblowing intentions. |
| **Radulovic and Uys (2019)** | 405 undergraduate students at a university in South Africa. | A questionnaire that assessed whistleblowing intentions for academic dishonesty, general honesty, academic honesty, justification for dishonest behavior, and justification for reporting/non-reporting. | Quantitative observational | Unclear | ◦ Fear of retaliation from wrongdoers decreased the whistleblowing likelihood for severe incidences. ◦ Whistleblowing intentions were positively related to general honesty, academic honesty, and adherence to principles. |
| **Reckers-Sauciuc and Lowe (2010)** | 65 M.B.A. students at two universities in the U.S. | A questionnaire that consisted of ten scenarios with questions assessing whistleblowing intentions, tolerance to ambiguity, and dispositional affect. | Quantitative experimental | Unclear | ◦ Negative (specifically sadness and fear) and positive affect (specifically happiness and arousal) influenced whistleblowing. |
| **Rennie and Crosby (2002)** | 500 medical students at a university in Scotland. Study 1: 461 medical students at a university in Scotland. Study 2: Between 25 and 40 medical students at a university in Scotland. | Study 1: A questionnaire that consisted of scenarios and questions assessing whistleblowing attitudes and intentions. Study 2: Focus groups that addressed the reasons for and against whistleblowing. | Mixed Methods observational | Unclear | ◦ Reasons for not whistleblowing were camaraderie, retaliation from peers, behaviorsis accepted despite being wrong, self-preservation, not responsible, no clear guidelines, futility, requirement of proof, and uncertain about what constitutes misconduct. ◦ Reasons for whistleblowing were consequences for patients, maintenance of standards, wrongdoer needing help, personal morality, being vindictive to wrongdoer, and to punish the wrongdoer. |
| **Richardson et al. (2012)** | 259 members of social Greek organizations (e.g., fraternity, sorority) in the U.S. (49.8% were female). | Participants were presented with one of three scenarios that varied on the severity level of hazing (not severe, moderately severe, or most severe). The participants were asked to indicate behavioral beliefs, outcome evaluations, attitude toward the behavior, normative beliefs, motivation to comply, subjective norms, and whistleblowing intentions. | Quantitative experimental | Unclear | ◦ Attitudes towards whistleblowing and subjective norms was positively related to whistleblowing intentions. ◦ Level of severity influenced whistleblowing intentions. |
| **Richardson et al. (2008)** | 330 public school teachers in the U.S. (88% were female). | A questionnaire comprised of scenarios and questions measuring communication confidence, policy attitudes, supervisor receptivity, and participatory culture. | Quantitative observational | High | ◦ Together, communication competence, policy attitude, supervisor receptivity, and participatory culture were positively related to whistleblowing intentions. ◦ Participatory culture was the primary contributor to the composite, while communication competence and policy attitude were moderate contributors. |
| **Robertson et al. (2011)** | 190 individuals with auditing experience (109 males and 81 females). | A questionnaire comprised of a scenario that varied on the likeability of the wrongdoer (more or less likeable) and performance reputation (high vs low) along with questions measuring whistleblowing intentions. | Quantitative experimental | Unclear | ◦ Likeability reputation is a significant determinant of whistleblowing intention. ◦ Performance reputation was marginally significant effect on whistleblowing intentions.  ◦ The whistleblowing intentions were greatest whenwrongdoers were less likeable and a poor performer. |
| **Robinson et al. (2012)** | 138 members of an internal auditor's association in the U.S. (59% were male). | Participants were presented with six scenarios that involved either financial statement fraud or theft of assets. The financial statement varied on the materiality of the statement. The theft scenarios varied whether the wrongdoer is aware that the potential whistleblower has knowledge of the theft (yes or no) and if others were aware (yes or no). For each scenario, the participants were asked to the likelihood that they would blow the whistle using an anonymous hotline and of other whistleblowing through anonymous hotline. | Quantitative experimental | Unclear | ◦ Whistleblowing was less likely to occur for financial statement fraud (vs theft), for immaterial financial statement fraud (vs material), when the wrongdoer is aware that thepotential whistleblower has knowledge of the wrongdoing, and when others are not aware of the wrongdoing. |
| **Rose et al. (2018)** | 115 MBA students from a U.S.-based university (43% were female). | All participants were randomly assigned to one of the four treatment conditions and presented with a scenario that varied on the form of compensation (restricted vs unrestricted stocks) and reward size (small vs large). The participants were asked to indicate their intentions to blow the whistle externally. | Quantitative experimental | Unclear | ◦ Whistleblowing intentions were influenced by the interaction between size of financial rewards and the type of stock in compensation plans.  ◦ Specifically, the reward size significantly influenced reporting intentions when they were paid with restricted stock but had little effect for unrestricted stock. |
| **Rothwell and Baldwin (2006)** | 382 government employees and police officers from the U.S. | A questionnaire that assessed ethical climate, and whistleblowing willingness and frequency. | Quantitative observational | Unclear | ◦ Supervisory status was positively related to whistleblowing intentions and whistleblowing frequency for minor wrongdoings.  ◦ A mandatory reporting policy was positively related to blowing the whistle for minor, major and misdemeanor violations. |
| **Rothwell and Baldwin (2007a)** | 382 police officers and state government employees from the U.S. | A questionnaire that assessed ethical climate, contextual independence, whistleblowing willingness, and frequency of whistleblowing. | Quantitative observational | High | ◦ Supervisory status is the most consistent predictor of whistleblowing. ◦ A mandatory reporting policy predicted the willingness to report several violations. |
| **Rothwell and Baldwin (2007b)** | 382 police officers and state government employees from the U.S. | A questionnaire that assessed ethical climate, whistleblowing willingness, and frequency of whistleblowing. | Quantitative observational | High | ◦ Supervisory status is the most consistent predictor of whistle-blowing intentions and behavior. ◦ A friendship or team climate demonstrates a capacity to predict willingness to blow the whistle. |
| **Rustiarini and Sunarsih (2017)** | 125 auditors from Indonesia (56.8% were male). | A questionnaire comprised of scales assessing attitudes towards whistleblowing, subjective norms, perceived behavioral control, and whistleblowing intentions. | Quantitative observational | High | ◦ Perceived behavioral control was positively associated with whistleblowing intentions. |
| **Satalkar and Shaw (2018)** | 33 researchers working in life science and medicine in Switzerland. | A semi-structured interview that explored on the importance of whistleblowing about scientific misconduct in their work environment and the reasons or circumstances that might deter them from doing so. | Qualitative observational | Unclear | ◦ Factors that might discourage whistleblowing were hierarchical research structures, fear of retaliation and damaging professional relationships, not possessing evidence to support one’s concerns/suspicions about misconduct, and prior negative whistleblowing experiences. |
| **Scheetz and Fogarty (2019)** | 99 individuals with bookkeeping or accounting experience (51.5% were female). | First, participants completed a questionnaire about their preconceived beliefs about how reports of unethical conduct would be managed along with their reactions if these beliefs were broken. Second, participants were presented with a scenario that varied on the ethical climate (positive or negative) and outcomes of previous reports (strong or weak). The participants were asked to indicate their whistleblowing intentions. | Quantitative experimental | Unclear | ◦ The failure to indicate a positive ethical environment increased whistleblowing intentions.  ◦ A weak managerial reacts to previous reports of unethical activity increased whistleblowing intentions.  ◦ Psychological contract violation mediates the relationship between previous reporting outcomes and whistleblowing intentions. |
| **Scheetz and Wilson (2019)** | 107 individuals with bookkeeping or accounting experience (61.7% were male). | Participants were presented with a scenario that varied on the type of organization (for-profit vs not-for-profit) and wrongdoing (asset misappropriation and financial statement fraud). The participants completed a questionnaire measuring public service motivation, affective and normative commitment and whistleblowing intentions. | Quantitative experimental | Unclear | ◦ Not-for-profit employees are more likely to blow the whistle regardless of the type of wrongdoing. |
| **Schultz et al. (1993)** | 145 managers and professional staff from France, Norway, and the U.S. | A questionnaire comprised of scenarios with questions assessing seriousness, responsibility, personal cost of reporting, and likelihood of whistleblowing. | Quantitative experimental | Unclear | ◦ National culture influenced whistleblowing intentions.  ◦ Perceptions of responsibility and seriousness was positively related to whistleblowing likelihood. ◦ Personal cost was negatively related to whistleblowing intentions. |
| **Seifert et al. (2014)** | 226 internal auditors and management accountants (52% were female). | Participants completed a survey containing of scenarios that varied on whistleblowing procedure fairness (fair vs unfair), whistleblowing process outcome fairness (fair or unfair), and management interaction fairness (fair or unfair). The participants were asked to indicate the likelihood of internal whistleblowing and trust of supervisor and organization. | Quantitative experimental | Unclear | ◦ Whistleblowing intentions is significantly correlated with organizational justice (procedural, distributive, and interactive). ◦ Whistleblowing intentions is significantly correlated with organizational and supervisor trust. ◦ Trust in both the supervisor and organization mediated the process between organizational justice and the likelihood of internal whistleblowing. |
| **Seifert et al. (2010)** | 447 internal auditors and management accountants (230 females and 216 males). | Participants completed a survey containing of scenarios that varied on procedural justice (fair vs unfair), distributive justice (fair or unfair), and interaction justice (fair or unfair). The participants were asked to indicate the likelihood of internal whistleblowing. | Quantitative experimental | Unclear | ◦ High levels of each form of organizational justice (i.e., procedural, interactional, and justice) increased the perceived likelihood of whistleblowing. |
| **Shawver (2008)** | 57 accounting students from a U.S.-based university (26 females and 31 males). | A questionnaire comprised of scenarios and questions assessing ethics and moral sensitivity, and whistleblowing intentions. | Quantitative observational | High | ◦ Whistleblowing intention had a positive relationship with materiality levels. ◦ The guarantee of a job increased whistleblowing intentions. |
| **Shawver (2011a)** | 40 accounting students from a U.S.-based university. | The participants completed a questionnaire at the start and the end of a twelve-week accounting ethics module/course. The questionnaire consisted of a scenario with questions assessing its ethicality and likelihood of whistleblowing to different organizations. | Quantitative experimental | Unclear | ◦ Ethics training increased whistleblowing intentions. |
| **Shawver (2011b)** | 157 attendees of a management accounting association meeting (91 males, 63 females, and 3 undisclosed). | A questionnaire consisted of scenarios and questions that measured moral intensity, moral judgment, and whistleblowing behavior. | Quantitative observational | High | ◦ The perceived overall harm and social pressure negatively influenced whistleblowing intentions. |
| **Sims and Keenan (1999)** | 86 managers from the U.S. (n = 44; 86% were male) and Jamaica (n = 42; 34% were female). | A questionnaire that measured moral perceptions for different types of wrongdoings, whistleblowing intentions, organizational and individual propensity for whistleblowing, and degree of fear of retaliation for whistleblowing | Quantitative observational | Low | ◦ Whistleblowing likelihood for major fraud was greater among U.S.-based managers than Jamaican managers. |
| **Sims and Keenan (1998)** | 248 business students (55% were male). | A questionnaire that consisted of scenarios with questions measuring whistleblowing responses, the impact of formal and informal policies, ideal values, supervisor expectations, job satisfaction, and organization commitment. | Quantitative observational | Low | ◦ Supervisor support and informal policies were predictors of external whistleblowing. ◦ Females were less likely to blow the whistle externally. ◦ Those individuals who believe a particular ethical situation should be resolved externally were more likely blow the whistle externally when they encounter that particular situation.  ◦ External whistleblowing was not predicted by formal policies, organizational tenure, age, education, satisfaction, or commitment. |
| **Singer et al. (1998)** | 133 individuals. Study 1: 53 employees at a large international baking firm based in New Zealand (30 females and 20 males). Study 2: 80 undergraduate students at a university in New Zealand (34 males and 46 females). | Study 1: Participant were presented with one of two sets of scenarios that varied according to proximity (high or low). The participants were asked to assess their moral intensity, empathy for the potential victim, and whistleblowing intentions. Study 2: A questionnaire that consisted of scenarios and questions assessing moral intensity, likelihood of action, perceived fairness, overall ethicality of the decision, and the need-for-cognition. | Quantitative experimental and observational | Unclear | ◦ Whistleblowing intentions had a positive relationship with empathy for the potential victim and magnitude of consequences. ◦ Whistleblowing intentions had a negative relationship with likelihood of consequences. |
| **Smith et al. (2001)** | 163 students from a U.S.-based university (55% were female). | Participants were presented with one of four scenarios that varied according to the level of impact (high or low) and level of wrongdoing (high or low). The participants were asked questions that assessed personal responsibility to report, perceived impact of reporting, perception of wrongdoing, perceived risk propensity, perceived project risk, and whistleblowing intentions. | Quantitative experimental | Unclear | ◦ Personal responsibility to report was negatively associated with the reluctance to blow the whistle through various internal channels. ◦ The assessment of whether the status ought to be reported had an indirect influence on the reluctance to blow the whistle through the assessment of personal responsibility to report. ◦ Perceived risk of negative consequences was negatively related to reluctance to blow the whistle through various internal and external channels. |
| **Somers and Casal (2011)** | 613 management accountants in the U.S. (81% were male). | A questionnaire that measured the participants' awareness and reporting of organizational wrongdoings. | Quantitative observational | High | ◦ The type of observed wrongdoing influenced the likelihood of whistleblowing. |
| **Somers and Casal (1994)** | 613 management accountants (81% males). | A questionnaire that assessed awareness of organizational wrongdoing, whistleblowing intentions, and organizational commitment. | Quantitative observational | High | ◦ Moderate levels of commitment were most likely to blow the whistle. |
| **Soni et al. (2015)** | 157 trainee accountants at a large audit firm in South Africa (80 males and 77 females). | A questionnaire that consisted of three scenarios with questions that assessed the whistleblowing likelihood. Each scenario varied according to the state of one form of organizational justice (procedural, distributive and interactional; high or low) | Quantitative experimental | Unclear | ◦ High levels of distributive, procedural and interactional justice were associated with an increased likelihood of whistleblowing. |
| **Stansbury and Victor (2009)** | 1417 individuals | 2003 National Business Ethics Survey | Quantitative observational | High | ◦ Perceptions of informal prosocial control had a relationship with whistleblowing. |
| **Stikeleather (2016)** | 118 individuals in the U.S. | Participants were assigned randomly to a fixed role of either an employer or a worker in one of three conditions (no whistleblowing, rewarded whistleblowing, and unrewarded whistleblowing). In all conditions, the employers are tasked with offering a wage to the worker, who can either could either accept or reject it. In the rewarded and unrewarded whistleblowing conditions and when the wage is accepted, the worker could observe a theft of revenue and then offered the opportunity to report it. | Quantitative experimental | Unclear | ◦ Offering a financial reward for internal whistleblowing increased the rate of reporting wrongdoings. ◦ The amount of compensation paid and the strength of moral convictions about whistleblowing influenced whistleblowing intentions. |
| **Stöber et al. (2019)** | 1005 managers from a multinational European company. | After randomly assignment to one of two conditions (code and training vs no code and no training), the participants were presented with a series of ethical dilemmas, where they had to decide how to respond. | Quantitative experimental | Unclear | ◦ A compliance program code increased whistleblowing intentions. |
| **Su et al. (2010)** | 549 accounting students from universities in the U.S. (n=286; 60.3% were female) and Taiwan (n=263; 39.7% were female). | A questionnaire that consisted of scenarios with questions related to whistleblowing intentions and ethical attitudes. | Quantitative observational | High | ◦ Members of a society described as collectivist, with high power distance, and strong uncertainty avoidance values are less likely to blow the whistle. ◦ Male are less willing to blow the whistle when there are no legal violations. |
| **Surya et al. (2017)** | 90 police officers in Indonesia. | A questionnaire measuring whistleblowing intentions, perceived behavioral control, and professional commitment. | Quantitative observational | Unclear | ◦ Perceived behavioral control was positively associated with whistleblowing intentions.  ◦ Professional commitment was positively related to whistleblowing intentions. |
| **Tan et al. (2003)** | 354 working professionals from the U.S. (n = 162; 44% were female) and Singapore (n = 192; 40% were female). | The participants were presented with one of four scenarios that varied according to organizational climate (conducive or non-conducive) and information asymmetry (sustainable or unsustainable). The participants were asked to indicate their whistleblowing intentions. | Quantitative experimental | Unclear | ◦ The relationship between organizational climate and whistleblowing intentions were stronger in an individualistic (i.e., U.S.) than in a collectivistic culture (i.e., Singapore).  ◦ The relationship between information asymmetry and whistleblowing intentions were stronger in a collectivistic (i.e., Singapore) than in an individualistic culture (i.e., U.S.). |
| **Tavakoli et al. (2003)** | 292 managers from firms in Croatia (n = 106; 51% were male) the U.S. (n = 186; 73% were male) | A questionnaire that measured moral perceptions, likelihood of whistleblowing, organizational and personal propensity for whistleblowing, and the degree of fear of retaliation. | Quantitative observational | Low | ◦ U.S. managers had stronger individual and organizational tendencies to blow the whistle, less fear of retaliation for whistleblowing, and more likely to blowing the whistle on major fraud than Croatian managers |
| **Taylor and Curtis (2010)** | 120 senior auditors (50% were female). | A questionnaire comprised of scenarios with questions assessing whistleblowing intentions, perseverance of reporting intent, professional identity, locus of commitment, and moral intensity. | Quantitative observational | High | ◦ Moral intensity was related to whistleblowing intentions and reporting perseverance.  ◦ High levels of professional identity increased the likelihood of initially reporting a wrongdoing. ◦ Commitment to the organization influenced a whistleblower's perseverance in reporting a wrongdoing. |
| **Taylor and Curtis (2013)** | 106 senior auditors based in the U.S. (57% were male). | Participants were asked to complete one of four questionnaires consisting of a scenario that varied on the dimensions of prior organizational response (responsive or unresponsive) and relative position wrongdoer (peer or superior). The participants assessed on their whistleblowing intentions and moral intensity | Quantitative experimental | Unclear | ◦ Individuals are more likely to blow the whistle on their peers than on their superiors. ◦ The reporting of a superior was more likely to occur when prior organizational response was strong (vs a weak response). ◦ Males were less sensitive to variations in power distance or prior organization response than women in terms of whistleblowing intentions.  ◦ Moral intensity was related to whistleblowing intentions. ◦ Power distance (peer vs superior) moderated the effect of moral intensity on whistleblowing intentions. Specifically, those individuals with a lower moral intensity are more influenced by power distance. |
| **Taylor and Curtis (2018)** | 120 public accountants from the U.S. (45% were female). | A questionnaire comprised of a scenario with questions measuring whistleblowing intentions, satisfaction with mentor relationships, ethical climate, trust in whistleblowing process, and organizational commitment. | Quantitative observational | Low | ◦ High quality mentoring relationship increased whistleblowing intentions. ◦ Trust in organizational response to reporting and affective organizational commitment mediated the relationship between mentor relationship quality and whistleblowing intention. ◦ The caring dimension of ethical climate was related to whistleblowing intentions. ◦ Trust in organizational response to reporting and affective commitment mediated in the relationship between caring dimension of ethical climate and whistleblowing intentions. |
| **Taylor (2018)** | 10685 employees in the Australian Public Service in 2013 (n = 7639) and 2016 (n = 3046) | Australian Public Service Employee Census from 2013 and 2016. | Quantitative observational | Low | ◦ Reasons for not blowing the whistle internally included the organization might ignore their reporting, the organization failed to take corrective action against ethical violations, and a fear of retaliation/reprisal. ◦ Perception of trustworthiness of senior managers was positively related to internal whistleblowing. |
| **Taylor (2019)** | 3495 respondents who witnessed corruption in the past 12 months (57.6% were female). | 2016 Australian Public Service Employee Census. | Quantitative observational | Low | ◦ Most types of corruption were found to be positively related to internal whistleblowing, with exception of theft of official assets, cronyism, and nepotism. ◦ Whistleblowing was found to be less prevalent in a hierarchy organizational culture (i.e., rules and procedures govern what people do). ◦ Individuals who have blown the whistle (active observers) after witnessing someone else being bullied were three times more likely to internally report corruption than an inactive observers of bullying. |
| **Teichmann (2019)** | 35 public officials who committed corruption and 35 anti-bribery experts from Austria, Germany, Liechtenstein, and Switzerland. | Informal and formal interviews. | Qualitative observational | High | ◦ Significant financial reward increased whistleblowing. |
| **Thoroughgood et al. (2011)** | 302 undergraduate students from a U.S.-based university (218 females and 84 males). | Participants were randomly assigned to one of 12 conditions that varied on the dimensions of organizational climate (tolerant or intolerant), leader sex (male or female), and organizational performance (negative, average, and positive). The participants were assessed on their perception of aversive leadership, whistleblowing intentions, moral intensity, trait cynicism, need for leadership, locus of control, and ambivalent sexism. | Quantitative experimental | Unclear | ◦ Aversive leaders elicited greater whistle-blowing intentions in financially unstable organizations that had ethical climates that were intolerant to negative behaviors of leaders. |
| **Trevino and Victor (1992)** | 721 individuals.  Study 1: 478 business students from a college (270 males and 208 females). Study 2: 115 students with fast-food experience (61 males and 54 females). Study 3: 128 fast-food restaurant employees (58% were female). | Study 1 and 2: A questionnaire that consisted of a scenario with questions related to perceptions of whistleblowing behavior, the ethicality and likability of the whistleblower, emotional reactions to whistleblowing, and whistleblowing intentions. A different scenario was used in study 1 and 2. Study 3: A questionnaire that measured group interests, role responsibility, evaluation of peer reporting, and whistleblowing intentions. | Quantitative observational | Unclear | ◦ Whistleblowing intentions increased with the establishment of a code of conduct that makes whistleblowing the responsibility of group members. ◦ Whistleblowing intentions increased when other group members suffered negative consequences from the wrongdoing. |
| **Trongmateerut and Sweeney (2013)** | 302 accounting students from universities in the U.S. (n=78; 45 males and 33 females) and Thailand (n=224; 206 females and 17 males). | A questionnaire that consisted of a scenario with questions that measured whistleblowing subjective norms, attitudes, and intentions. | Quantitative experimental | High | ◦ Subjective norms for whistleblowing not only had a direct effect on whistleblowing attitudes but also a direct and indirect (via attitudes) effect on whistleblowing intentions.  ◦ The whistleblowing intentions of participants from a collectivist culture were more strongly influenced by subjective norms for whistleblowing. |
| **Tumuramye et al. (2018)** | 222 employees of procuring and disposing entities in Uganda (56.3% were female). | A questionnaire that assessed ethical climate, whistleblowing expectancies, and whistleblowing support institutions. | Quantitative observational | High | ◦ The whistleblowing supporting institutions and ethical climate were significant predictors of whistleblowing intentions and behavior. ◦ Whistleblowing expectancy was a significant predictor to whistleblowing intentions and behavior. |
| **Ugaddan and Park (2019)** | 8000 respondents who observed wrongdoings. | The Merit Principles Survey (MPS) 2010 questionnaire. | Quantitative observational | High | ◦ Trustful administrative leadership was associated with whistleblowing intentions. ◦ Procedural justice was positively associated with whistleblowing intentions. ◦ Organizational fairness may increase whistleblowing likelihood. ◦ A positive relationship between public service motivation and whistleblowing intentions. ◦ The relationship between whistleblowing intentions and both trust in leadership and organizational justice was partially mediated by public service motivation and extrinsic motivation. |
| **Victor et al. (1993)** | 169 fast food restaurant employees (68% were female). | A questionnaire that measured whistleblowing intentions and behavior, perceptions of justice, role responsibility, and interests of group members. | Quantitative observational | High | ◦ Whistleblowing intentions were positively associated with procedural justice evaluations, role responsibility, and interests of group members.  ◦ Whistleblowing behavior was associated with whistleblowing intentions and retributive justice evaluations. |
| **Vincent et al. (1999)** | 198 obstetricians (n = 42) and midwives (n = 156). | A questionnaire that assessed knowledge of incident-reporting system, staff estimates of whistleblowing likelihood, and the potential reasons for not reporting. | Quantitative observational | High | ◦ Reasons for not whistleblowing were fears that junior staff would be blamed, high workload, and the belief that the circumstances or outcome of a particular case did not warrant a report despite it being designated as reportable. |
| **Wainberg and Perreault (2016)** | 68 university students with significant auditing experience. | Participants were presented with one of four scenarios that varied on the dimensions of explicitness of mentioning protection from retaliation (explicit or non-explicit) and job security (high or low). The participants were asked to indicate the likelihood of whistleblowing and the perception of risk. | Quantitative experimental | Unclear | ◦ The likelihood of whistleblowing when the retaliation protection policy was explicitly stated was lower than the whistleblowing likelihood in the non-explicit condition. |
| **Wen and Chen (2016)** | 172 managers enrolled in business courses a university in China (89 males and 83 females). | Two questionnaires separated by 4 months. The first questionnaire assessed ethical leadership of direct supervisor and power distance. The second questionnaire measured moral intensity and whistleblowing intentions. | Quantitative observational | Low | ◦ Ethical leadership was positively related to whistleblowing intention. ◦ Moral identity mediated the relationship between ethical leadership and whistleblowing intentions ◦ Power distance orientation positively moderated the relationship between ethical leadership and whistleblowing intentions. |
| **Whitaker et al. (2014)** | 9 national level athletes from track and field (n=4) and rugby league (n=5; 2 females and 7 males). | A semi-structured interview that focused on the athletes’ perceptions about reporting doping. | Qualitative observational | Unclear | ◦ Negative emotions had a positive influence on their willingness to blow the whistle.  ◦ Factors discouraging willingness to report doping were lack of knowledge, the potential repercussions, feelings of helplessness to stop others, and loyalty to teammates and their sport. |
| **Wilson et al. (2018)** | 235 individuals (54% were female). | Participants were presented with a scenario that varied on the dimensions of firm tenure (new, 2, 7, and 15 years) and auditor familiarity (met or never met). The participants were asked to indicate whistleblowing intentions and level of trust in the firm and the auditor. | Quantitative experimental | Unclear | ◦ Trust in an individual and trust in a firm were positively associated with whistleblowing intentions. ◦ The relationship between of auditor familiarity and whistleblowing intentions was mediated by auditor trust. |
| **Xu and Ziegenfuss (2008)** | 201 internal auditors (77 females and 108 males). | A questionnaire comprised of a scenario in which an internal auditor discovered the occurrence of wrongdoing and a scale that measure of an individual’s ethical reasoning and judgement. For the scenario, the participants were asked to indicate if the individual should report the wrongdoing and predict whether they would report. The situations in the scenario were modified to include details about the employer's reward systems. | Quantitative experimental | Unclear | ◦ The availability of monetary incentives and employment protections encouraged whistleblowing. ◦ Whistleblowers with relatively low levels of moral reasoning are more likely to be influenced by the monetary incentives than those with relatively high levels of moral reasoning. |
| **Yu et al. (2019)** | 1040 food processing and food service employees in the U.S. and Canada (42% were female). | A questionnaire that measured job satisfaction, food safety self-efficacy, perceived food safety training effectiveness, and food safety whistleblowing. | Quantitative observational | Low | ◦ Perceived effectiveness of training increased food processing employees’ whistleblowing by increasing food safety self-efficacy. ◦ Perceived effectiveness of training increased food service employees’ whistleblowing by increasing job satisfaction.  ◦ Males were more likely to blow the whistle than females with the same level of food safety self-efficacy and job satisfaction. |
| **Zarefar and Zarefar (2017)** | 123 professionals (52 auditors and 71 non auditor). | A questionnaire that measured the participants' ethics and locus of control. | Quantitative observational | High | ◦ Locus of control was negatively associated with whistleblowing intentions ◦ Ethics was positively related to whistleblowing intentions. ◦ An individual's profession moderated the relationship between whistleblowing intentions and both ethics and locus of control. |
| **Zhang et al. (2016)** | 83 supervisors (74.2% were male) and 412 (52.2% were male) subordinates from a bank in China. | For leader–member dyads, a questionnaire was distributed that assessed ethical leadership, collective moral potency, ethical climate, personal identification, and whistleblowing. | Quantitative observational | High | ◦ Ethical leadership was positively associated to internal whistleblowing. ◦ After controlling for ethical climate, collective moral potency and personal identification with a superior mediated the relationship between ethical leadership and internal whistleblowing. |
| **Zhang et al. (2013)** | 130 M.B.A. students (87 males and 43 females). | Participants were presented with a scenario that varied on the dimensions of the anonymous hotline administrator (internal or external) and previous whistleblowing outcomes (negative or positive). The participants were asked to indicate their proactivity trait and whistleblowing intentions. | Quantitative experimental | Unclear | Externally administered hotline resulted in higher whistleblowing intentions when previous whistleblowing outcomes were negative. Individuals that were more passive and less proactive had a greater likelihood of whistleblowing to an externally administered hotline. High proactive individuals were more likely to blow the whistle than low proactivity individuals. |
| **Zhang et al. (2009a)** | 364 bank employees from China (48.6% were female). | A questionnaire that measured whistleblowing judgment, whistleblowing intentions, organizational ethical culture, and mood. | Quantitative observational | Low | ◦ Whistleblowing judgment was positively related to whistleblowing intentions. ◦ Organizational ethical culture positively moderated the relationship between whistleblowing judgment and intentions. |
| **Zhang et al. (2009b)** | 364 bank employees from China (48.6% were female). | A questionnaire that measured whistleblowing judgment, whistleblowing intentions, organizational ethical culture, and mood. | Quantitative observational | Low | ◦ Whistleblowing judgment was positively related to whistleblowing intentions. ◦ Organizational ethical culture positively moderated the relationship between whistleblowing judgment and intentions. ◦ Interaction between organizational ethical culture and positive mood had a moderating effect on the whistleblowing judgment and intention relationship. |
| **Zhang (2008)** | 60 undergraduate business students. | After randomly assigning the participants to the role of principal and agent, the latter individuals were tasked with reporting their 'production costs' and whether a peer has overstated their costs to the former. The principal decided on the issued wages. Under one condition, the agents were able to communicate with each other before their reports, whereas in the other condition, there was no communication between agents. | Quantitative experimental | Unclear | ◦ Perceived fairness of the principal and communication between peers influenced whistleblowing behavior. |
| **Zheng et al. (2019)** | 371 internal auditors in China (188 males and 183 females). | A questionnaire that contained a scenario with questions assessing whistleblowing intentions, ethics, and self-construal. | Quantitative observational | High | ◦ Those individuals with interdependent self-construals were less likely to engage in whistleblowing. |
| **Zhou et al. (2018)** | 667 employees of organizations in China (51.1% were male). | Two questionnaires separated by 2 months. The first questionnaire assessed ethical climate, organizational identification, moral identity, and risk aversion. The second questionnaire measured whistleblowing intentions. | Quantitative observational | low | ◦ Perceived ethical climate was positively related to whistleblowing intention. ◦ The relationship between perceived ethical climate and whistleblowing intentions was mediated by organizational identification and moral identity. ◦ Risk aversion negatively affected on the influence of organizationalidentification on the perceived ethical climate-whistleblowing intention relationship. |
| **Zhuang et al. (2005)** | 159 accounting students from Canada (n = 77; 42 females and 34 males) and China (n = 82; 40 females and 42 males). | Participants were presented with one of two sets of three cases. Two of the cases varied according to the perpetrator of the wrongdoing (peer or supervisor). The participants were asked to indicate their whistleblowing intentions, seriousness of the wrongdoings, personal responsibility to report, and personal cost of reporting. | Quantitative experimental | Unclear | ◦ The likelihood of blowing the whistle on a peer was higher among Chinese respondents than Canadians.  ◦ Chinese respondents were more likely to blow the whistle on their peers than supervisors. |
| **Zipparo (1999)** | 800 public officials from Australia. | A questionnaire that measured knowledge of whistleblowing channels, attitudes towards reporting, and factors that deter whistleblowing. | Quantitative observational | High | ◦ Factors that discouraged whistleblowing were the absence of 'sufficient' proof and the absence of protective legislation. ◦ Different groups were deterred from blowing the whistle by different circumstances. |

**References Cited in Supplementary Tables Only**

Barnett, T., Bass, K., and Brown, G. (1996). Religiosity, ethical ideology, and intentions to report a peer’s wrongdoing. J. Bus. Ethics 15, 1161–1174. doi:10.1007/BF00412815.

report a peer’s wrongdoing. J. Bus. Ethics 15, 1161–1174. doi:10.1007/BF00412815.

Barnett, T., Cochran, D. S., and Taylor, G. S. (1993). The internal disclosure policies of private-sector employers: An initial look at their relationship to employee whistleblowing. J. Bus. Ethics 12, 127–136. doi:10.1007/BF00871932.

Bjorkelo, B., Thorsen, C., D’Cruz, P., and Mikkelsen, E.G. (2020). Whistleblowing and bullying at work: The role of leaders. In P. D’Cruz, E. Noronha, L. Keashly& S. Tye-Williams (Eds). Handbooks of workplace bullying, emotional abuse and harassment: Special topics and particular occupations, professions and sectors. Singapore: Springer.

Black, L. M. (2011). Tragedy in policy: A quantitative study of nurses’ attitudes toward patient advocacy activities. Am. J. Nurs. 111, 26–35. doi:10.1097/01.NAJ.0000398537.06542.c0.

Fredin, A. J. (2011). The effects of anticipated regret on the whistleblowing decision. Ethics Behav. 21, 404–427. doi:10.1080/10508422.2011.604296.

Jackson, D., Peters, K., Andrew, S., Edenborough, M., Halcomb, E., Luck, L., et al. (2010). Understanding whistleblowing: Qualitative insights from nurse whistleblowers. J. Adv. Nurs. 66, 2194–2201. doi:10.1111/j.1365-2648.2010.05365.x.

Ion, R., Smith, K., Nimmo, S., Rice, A. M., and McMillan, L. (2015). Factors influencing student nurse decisions to report poor practice witnessed while on placement. Nurse Educ. Today 35, 900–905. doi:10.1016/j.nedt.2015.02.006.

Jenkel, I., and Haen, J. J. (2012). Influences on students’ decisions to report cheating: A laboratory experiment. J. Acad. Ethics 10, 123–136. doi:10.1007/s10805-012-9154-7.

Kaplan, S. E. (1995). An examination of auditors’ reporting intentions upon discovery of procedures prematurely signed-off. Auditing 14, 90–114.

Kaplan, S. E., Pope, K. R., and Samuels, J. A. (2011). An examination of the effect of inquiry and auditor type on reporting intentions for fraud. Auditing-J. Pract. Th. 30, 29–49. doi:10.2308/ajpt-10174.

Kaplan, S. E., and Schultz, J. J. (2007). Intentions to report questionable acts: An examination of the influence of anonymous reporting channel, internal audit quality, and setting. J. Bus. Ethics 71, 109–124. doi:10.1007/s10551-006-0021-6.

Keenan, J. P. (1995). Whistleblowing and the first-level manager: Determinants of feeling obliged to blow the whistle. J. Soc. Behav. Pers. 10, 571–584.

Keenan, J. P. (2002b). Whistleblowing: A study of managerial differences. Empl. Responsib. Rights J. 14, 17–32. doi:10.1023/A:1015796528233.

Li, S. M., and Ma, W. W. K. (2016). Exploring group effects on individual ethical judgements and whistleblowing decisions of accountancy students. JCAR 5. doi:10.21863/jcar/2016.5.1.021.

MacNab, B. R., Brislin, R., Worthley, R., Galperin, B. L., Jenner, S., Lituchy, T. R., et al. (2007). Culture and ethics management: Whistle-blowing and internal reporting within a NAFTA country context. Int. J. Cross Cult. Manag. 7, 5–28. doi:10.1177/1470595807075167.

Nayir, D. Z., Rehg, M. T., and Asa, Y. (2018). Influence of ethical position on whistleblowing behaviour: Do preferred channels in private and public sectors differ? J. Bus. Ethics 149, 147–167. doi:10.1007/s10551-016-3035-8.

Near, J. P., Baucus, M. S., and Miceli, M. P. (1993). The relationship between values and practice: Organizational climates for wrongdoing. Adm. Soc. 25, 204–226.

Previtali, P., and Cerchiello, P. (2018). The determinants of whistleblowing in public administrations: An analysis conducted in Italian health organizations, universities, and municipalities. Public Manag. Rev. 20, 1683–1701. doi:10.1080/14719037.2017.1417468.

Reckers-Sauciuc, A. K., and Lowe, D. J. (2010). The influence of dispositional affect on whistle-blowing. Adv. Account. 26, 259–269. doi:10.1016/j.adiac.2010.05.005.

Stöber, T., Kotzian, P., and Weißenberger, B. E. (2019). Design matters: on the impact of compliance program design on corporate ethics. Bus. Res. 12, 383–424. doi:10.1007/s40685-018-0075-1.

Zhang, J., Chiu, R., and Wei, L. (2009a). Decision-making process of internal whistleblowing behavior in China: Empirical evidence and implications. J. Bus. Ethics 88, 25–41. doi:10.1007/s10551-008-9831-z.

Zhang, J., Chiu, R., and Wei, L. (2009b). On whistleblowing judgment and intention: The roles of positive mood and organizational ethical culture. J. Manag. Psychol. 24, 627–649. doi:10.1108/02683940910989020.

Zhuang, J., Thomas, S., and Miller, D. L. (2005). Examining culture’s effect on whistle-blowing and peer reporting. Bus. Soc. 44, 462–486. doi:10.1177/0007650305281848.
